# The complete plastomes of thirteen *Libanotis* (Apiaceae, Apioideae) plants: comparative and phylogenetic analyses provide insights into the plastome evolution and taxonomy of *Libanotis*

**DOI:** 10.1186/s12870-024-04784-4

**Published:** 2024-02-12

**Authors:** Li-Jia Liu, Chang-Kun Liu, Jing Cai, Jiao-Jiao Deng, Xing‑Jin He, Song‑Dong Zhou

**Affiliations:** 1 Key Laboratory of Bio‑Resources and Eco‑Environment of Ministry of Education, College of Life Sciences, Sichuan University, Chengdu, 610065 China; 2 College of Resources Environment and Chemistry, Chuxiong Normal University, Chuxiong, 675000 China

**Keywords:** Apiaceae, *Libanotis*, Plastome, Phylogeny, Taxonomy

## Abstract

**Background:**

The genus *Libanotis* Haller ex Zinn, nom. cons., a contentious member of Apiaceae, encompasses numerous economically and medicinally significant plants, comprising approximately 30 species distributed across Eurasia. Despite many previous taxonomic insights into it, phylogenetic studies of the genus are still lacking. And the establishment of a robust phylogenetic framework remains elusive, impeding advancements and revisions in the taxonomic system for this genus. Plastomes with greater variability in their genetic characteristics hold promise for building a more robust *Libanotis* phylogeny.

**Results:**

During our research, we sequenced, assembled, and annotated complete plastomes for twelve *Libanotis* species belong to three sections and two closely related taxa. We conducted a comprehensive comparative analysis through totally thirteen *Libanotis* plastomes for the genus, including an additional plastome that had been published. Our results suggested that *Libanotis* plastome was highly conserved between different subclades, while the coding regions were more conserved than the non-coding regions, and the IR regions were more conserved than the single copy regions. Nevertheless, eight mutation hotspot regions were identified among plastomes, which can be considered as candidate DNA barcodes for accurate species identification in *Libanotis*. The phylogenetic analyses generated a robustly framework for *Libanotis* and revealed that *Libanotis* was not a monophyletic group and their all three sections were polygenetic. *Libanotis schrenkiana* was sister to *L. sibirica*, type species of this genus, but the remainders scattered within Selineae.

**Conclusion:**

The plastomes of *Libanotis* exhibited a high degree of conservation and was effective in enhancing the support and resolution of phylogenetic analyses within this genus. Based on evidence from both phylogeny and morphology, we propose the recognition of "*Libanotis* sensu stricto" and provide taxonomic recommendations for other taxa that previously belonged to *Libanotis*. In conclusion, our study not only revealed the phylogenetic position and plastid evolution of *Libanotis*, but also provided new insights into the phylogeny of the family Apiaceae and phylogenetic relationships within the tribe Selineae.

**Supplementary Information:**

The online version contains supplementary material available at 10.1186/s12870-024-04784-4.

## Background

*Libanotis* Haller ex Zinn, nom. cons., belonging to the tribe Selineae of the family Apiaceae, includes approximately 30 species distributed throughout Eurasia, with 19 species found in China [1–12]. *Libanotis* as an independent genus was supported by de Candolle [13], Schischkin [14], Korovin [15], Rechinger [16], Fu [17, 18], Shan, Watson and Sheh [1, 2, 11, 19]. They thought conspicuous calyx teeth, separated bracteoles, and hairy mericarps easily distinguished *Libanotis* from *Seseli*. But the genus then has been suggested to merge into *Seseli* L. to establish broad sense *Seseli* genus by Drude [20], Ball [21], Kljuykov and Pimenov [1, 22–26], because they think the above diagnostic features are not sufficient to distinguish them. The views of the above taxonomists are all based on morphology, and in the Chinese taxa the taxonomists are equally sharply divided between these two schools of thought, and some taxonomists all agree that *Libanotis* should be retained rather than merged [1–3, 12, 17–19, 27]. By 2015, new *Libanotis* taxa (*L. laoshanensis* W.Zhou & Q.X.Liu) were still being published [10]. Pimenov set aside the retention of the taxonomic status of *Libanotis* for this species untreated in the 2017 treatment of the Chinese Apiaceae taxa [24]. The above indicates that a thorough phylogenetic analysis of *Libanotis* is necessary. Regrettably, there has been no prior phylogenetic investigation conducted concerning this contentious genus *Libanotis*. Furthermore, all phylogenetic analyses have consistently demonstrated that the *Seseli* genus, in its broader sense, is polyphyletic, owing to the complex and perplexing variations in mericarps and vegetative body morphology. [26, 28–37]. Recently, *Seseli* s.s. was established and several phylogenetic studies using molecular fragments (nrITS and nrETS) robustly supported that *L. sibirica* C. A. Mey. (type species of *Libanotis*, *L. montana* Crantz ≡ *Seseli libanotis* W.D.J.Koch = *L. sibirica*) did not cluster with *Seseli tortuosum* L. (type species of *Seseli*) into a monophyletic branch [32, 33]. Hence, we believe that the taxonomic status of *Libanotis* needs to be discussed again, especially in China. Nevertheless, the delimitation of *Libanotis* genus still faced severely challenge. All previous phylogenetic studies showed that *Libanotis* was not a monophyletic group and members of this genus scattered in the Selineae tribe [6, 7, 35, 37, 38]. Due to limited sample and molecular fragments contained few informative loci, these studies all generated the phylogenetic framework with weak support and low resolution, which was insufficient to aid to the taxonomic revision of *Libanotis* members. Hence, it is imperative to establish a more comprehensive phylogenetic framework for *Libanotis* to address the controversy surrounding its evolutionary relationships and taxonomic status.

**Fig. 1:**
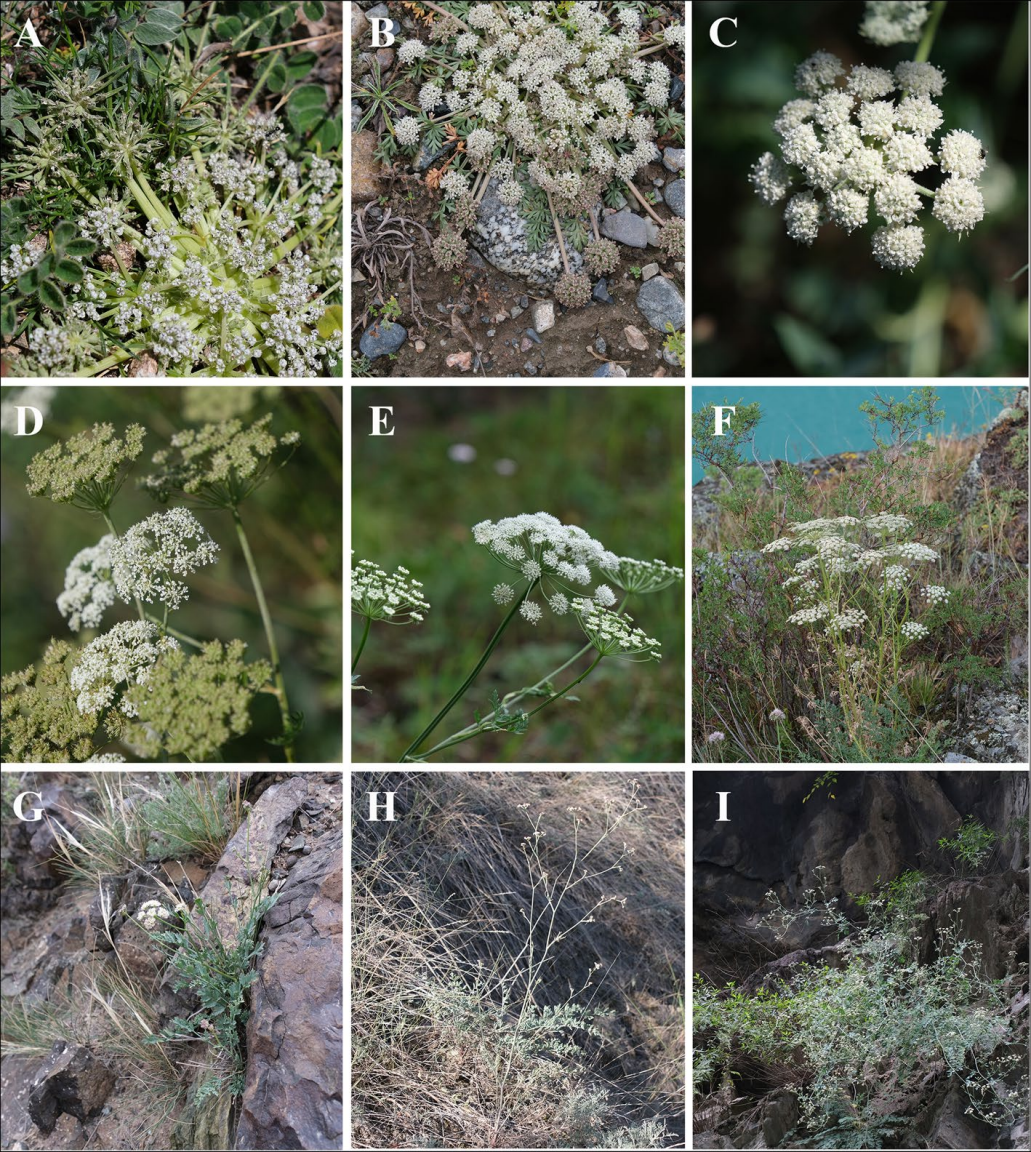
Some flowering *Libanotis* species with diverse morphology. **A, B-** Sect. *Pseudolibanotis* (**A**) *L. depressa* R.H.Shan & M.L.Sheh (**B**) *L. acaulis* R.H.Shan & M.L.Sheh **C, F, G, H, I**- Sect. *Eriotis* (**C**) *L. iliensis* (Lipsky) Korovin (**F**) *L. buchtormensis* DC. (**G**) *L. grubovii* (V.M.Vinogr. & Sanchir) M.L.Sheh & M.F.Watson (**H**) *L. lanzhouensis* K.T.Fu ex R.H.Shan & M.L.Sheh (**I**) *L. spodotrichoma* K.T.Fu. **D, E**- Sect. *Libanotis* (**D**) *L. seseloides* (Fisch. & C.A. Mey. ex Ledeb.) Turcz. (**E**) *L. sibirica* C.A.Mey. Photograph by Liu Li-Jia

Due to the large number of species under the genus *Libanotis*, many taxonomists have established sections under the genus. Among the opinions in favor of the independence of *Libanotis*, de Candolle [13] was the first to group them, arguing that *Libanotis* could be divided into two sections, Sect. *Eriotis* and Sect. *Eulibanotis*: in which Sect. *Eriotis* are “Petals covered with short fascicular hairs on the outside leaves coriaceous, thickish, shiny.”; and Sect. *Eulibanotis* are “Petals dorsally glabrous or with sparse simple short hairs, leaves not coriaceous, not shiny.” After a series of species transfers and the publication of new species, Schischkin [14] added two sections: Sect. *Pseudolibanotis* and Sect. *Schultziopsis*, Sect. *Pseudolibanotis* were described by “Main stem not developed, the root neck bearing slightly leafy, sometimes nearly leafless shoots which spread along ground or ascend.” And the trait of Sect. *Schultziopsis* are special, their subcapitate umbel surrounded by the rounded sheaths of terminal leaves. The four-sections system was widely accepted by taxonomists that supported the independence of *Libanotis* [5, 15]. *L. monstrosa* (Willd.) DC., the only species in Sect. *Schultziopsis*, has been used as the type species for the establishment of the new monotypic genus *Sajanella* Soják [39] and is therefore excluded from this study, while the other all three sections are included for phylogenetic analysis (Fig. 1, Table 1). Based on these section characteristics, the newly published species *L. jinanensis* [3] and the newly transferred species *L. grubovii* [19] could be included in Sect. *Eriotis* according to their descriptions (Table 1, Table S10).

**Table 1:** The taxonomic treatment of sections and part of important history of nomenclature of 13 *Libanotis* taxa in this study. Species scientific names in this study are based on the FOC treatment (Sheh and Watson, 2005), and "—" indicates that the species were not included in that treatment

Sheh and Watson (2005)	Schischkin (1950)-4 sections		Shan and Sheh (1985)-3 sections	Pimenov and Kljuykov (2005)	Pimenov (2017)
***L. depressa* R.H.Shan & M.L.Sheh**	Sect. *Pseudolibanotis*	—	Sect. *Pseudolibanotis*	*Seseli depressum* (R. H. Shan & M. L. Sheh) V. M. Vinogr	*Stenocoelium depressum* (Shan Renhwa et Sheh Menglan) Pimenov et Kljuykov
***L. acaulis* R.H.Shan & M.L.Sheh**		—		*Seseli acaule* (Shan Renhwa et Sheh Menglan) V. M. Vinogr	*Seseli acaule* (Shan Renhwa et Sheh Menglan) V. M. Vinogr
***L. spodotrichoma* K.T.Fu**	Sect. *Eriotis*	—	Sect. *Eriotis*	*Seseli spodotrichoma* (K.T.Fu) Pimenov	*Seseli spodotrichomum* (Fu Kuntsun) Pimenov
***L. iliensis* (Lipsky) Korovin**		—		*Seseli vaillantii* H. Boissieu	*Seseli vaillantii* H. Boissieu
***L. lanzhouensis* K.T.Fu ex R.H.Shan & M.L.Sheh**		—		*Seseli lanzhouense* (Fu Kuntsun) V. M. Vinogr	*Seseli lanzhouense* (Fu Kuntsun) V. M. Vinogr
***L. buchtormensis* (Fischer) de Candolle**		*L. buchtormensis* (Fischer) de Candolle		*Seseli buchtormense* (Spreng.) W. D. J. Koch	*Seseli buchtormense* (Spreng.) W. D. J. Koch
***L. incana* (Steph. ex Willd.) O. & B. Fedtsch**	Sect. *Eulibanotis*	—	Sect. *Libanotis*	*Seseli incanum* (Steph. ex Willd.) B. Fedtsch	*Seseli incanum* (Steph. ex Willd.) B. Fedtsch
***L. sibirica* (L.) C.A.Mey**		*L. sibirica* (L.) C.A.Mey		*Seseli libanotis* (L.) W. D. J. Koch	*Seseli libanotis* (L.) W. D. J. Koch
***L. schrenkiana* C.A.Mey. ex Schischk**		*L. schrenkiana* C.A.Mey. ex Schischk		*Seseli schrenkianum* (C. A. Mey. ex Schischk.) Pimenov & Sdobnina	*Seseli schrenkianum* (C. A. Mey. ex Schischk.) Pimenov et Sdobnina
***L. seseloides* (Fisch. & C.A.Mey. ex Ledeb.) Turcz**		*L. seseloides* (Fisch. & C.A.Mey. ex Ledeb.) Turcz		*Seseli seseloides* (Fischer & C. A. Meyer ex Turczaninow) M. Hiroe	*Seseli seseloides* (Turcz.) M. Hiroe
***L. condensata* (L.) Crantz**		*L. condensata* (L.) Crantz		*Seseli condensatum* (L.) H. G. Reichenbach	*Seseli condensatum* (L.) Rchb.f
	Sect. *Schultziopsis*	—			
***L. jinanensis* L.C.Xu & M.D.Xu**		—	—	*Seseli jinanense* (L.C.Xu & M.D.Xu) Pimenov	*Seseli jinanense* (Xu Lingchuan et Xu Mingde) Pimenov
***L. grubovii* (V.M.Vinogr. & Sanchir) M.L.Sheh & M.F.Watson**		—	—	*Seseli grubovii* V. M. Vinogr. et Sanchir	*Seseli grubovii* V. M. Vinogr. et Sanchir

Additionally, many plants of *Libanotis* have important medicinal value and are used as traditional Chinese medicinal materials. For example, six *Libanotis* taxa (*L. buchtormensis* (Fisch.) DC., *L. lancifolia* K.T.Fu., *L. spodotrichoma* K.T.Fu., *L. wannienchun* K.T.Fu., *L. lanzhouensis* K.T.Fu ex R.H.Shan & M.L.Sheh, and *L. sibirica*) are all known as the "Changchun Seven" in the Qinling Seven medicines, which is used to treat common cold, toothache, headache, traumatic injury, inflammation, swelling, rheumatism, respiratory diseases, as well as symptomatic coughs and dyspnea [40–42]. However, due to morphological feature exhibiting highly similar in inter-species and significant divergence in intra-species, the accurate identification of *Libanotis* species was extremely difficult [40]. Due to their morphological similarity, instances of homonym or synonym in common names exist in various regions and markets, making it challenging to distinguish them during collection, acquisition, and clinical usage. They are often mistakenly interchanged. For example, the above 'Changchun Seven' consists of six different species and is divided into sixteen varieties in herbal medicine, causing confusion in the herbal market [40]. Therefore, the selection of reliable molecular markers for ensuring the accurate identification of medicinal *Libanotis* species is of utmost importance.

The plastome was highly conserved in flowering plant and harbored sufficient variable loci [43, 44]. Hence, plastome data have been widely used in phylogenetic analyses and development of special DNA barcode in Apiaceae, Poaceae, Lamiaceae, Rosaceae, Liliaceae, *Allium*, *Artemisia*, and other plant taxa [44–52]. Regrettably, despite the presence of two *Libanotis* plastomes in GenBank, there has been a lack of plastid phylogenomic analysis conducted for this genus. In this study, we filled this gap by sequencing the plastid genomes of twelve taxa of *Libanotis*. Together with two plastomes previously reported, we conducted comprehensive analyses to (1) reveal the plastid characteristics and evolution of *Libanotis*; (2) identify suitable mutation hotspots from plastomes to use as candidate barcodes for species identification of *Libanotis*; (3) investigate the genus boundary of *Libanotis* and provide new sights into the phylogenetic position of this genus taxa distributed in China.

## Results

### Plastome features of *Libanotis* and repeat sequence analyses

**Fig. 2:**
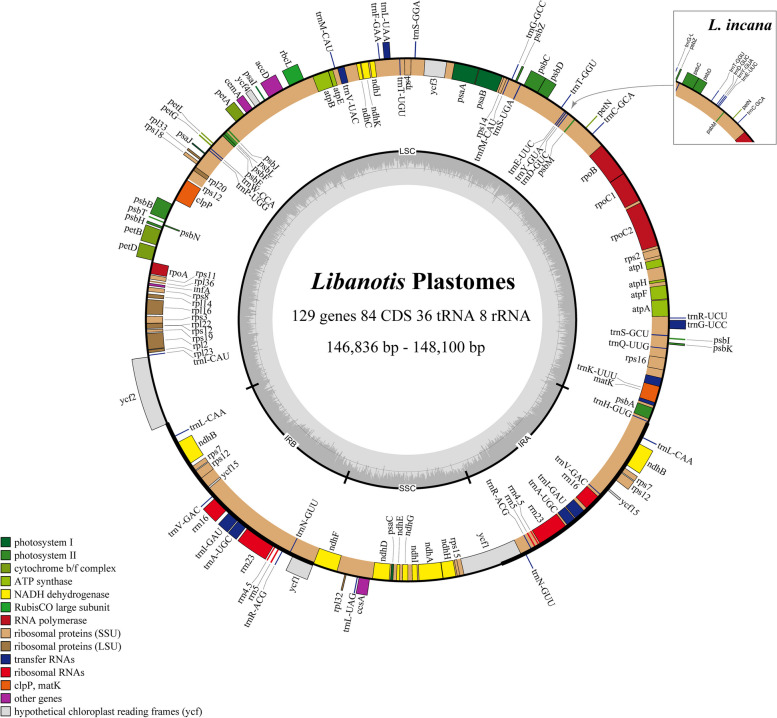
The gene map displays twelve newly sequenced *Libanotis* plastomes (*L. sibirica* was used as a representative). The inner circle's dark gray area indicates the GC content. Gene numbers and plastomes length are tagged inside. Genes outside the outer circle are transcribed clockwise, while inside are transcribed counterclockwise. Different gene functional groups are color-coded

**Fig. 3:**
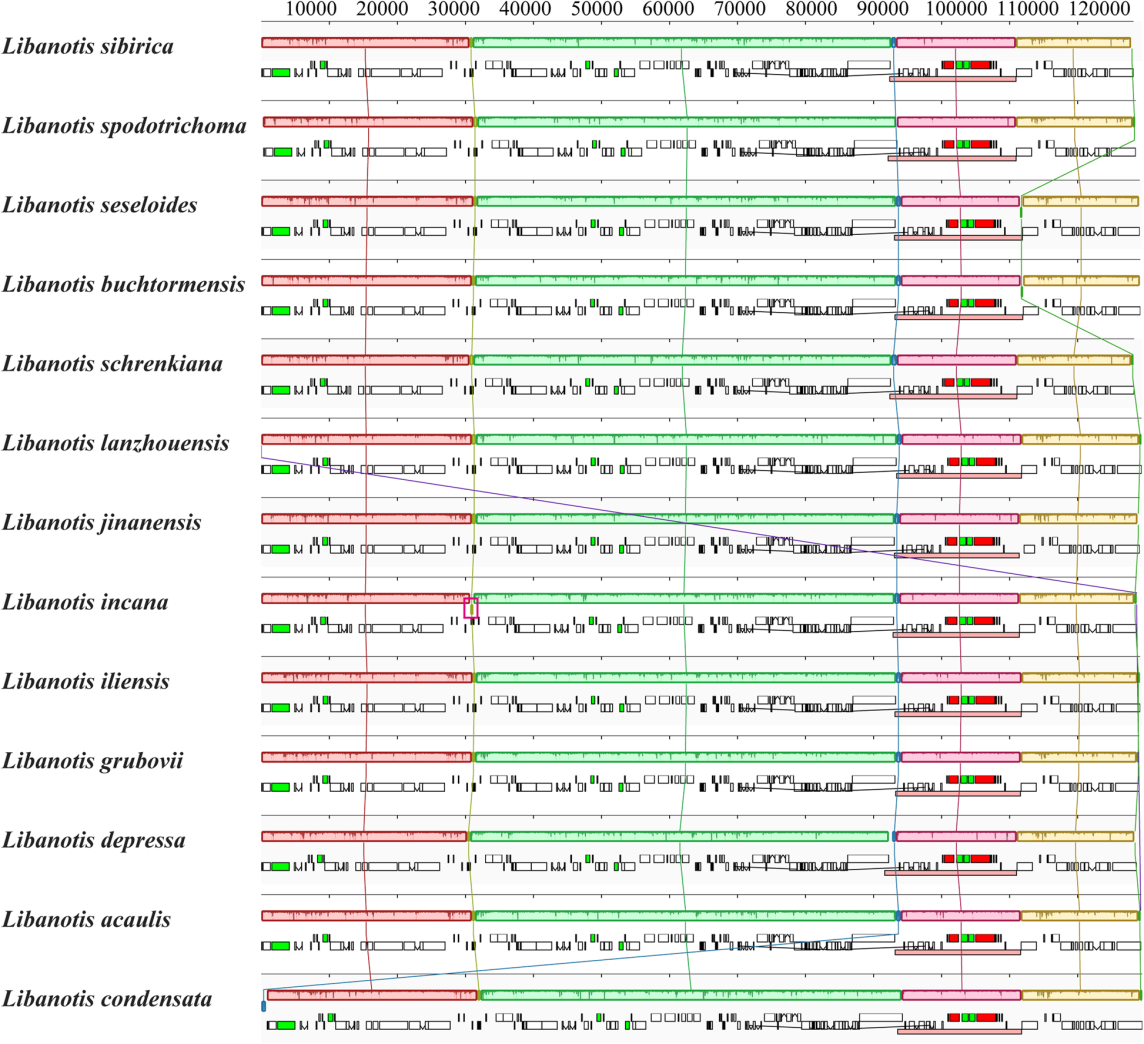
Mauve alignment of thirteen *Libanotis* plastomes, where blocks of the same color connected by lines indicate local collinear blocks within each alignment. The red boxes pick out are the inversion of the *trn*Y-*trn*D-*trn*E gene

The complete plastomes newly sequenced of 12 *Libanotis* species have been fully characterized, with sizes ranging from 146,836 bp (*L. sibirica*) to 148,100 bp (*L. depressa*) (Table 2, Fig. 2). Compared to other *Libanotis* taxa, *L. depressa* was particularly unique, with a significantly expanded IR region of length 19,437 and the GC content of only 43.7%. The analysis of the twelve *Libanotis* plastomes revealed a collective inventory of 129 genes, including 84 PCGs, 36 tRNA genes, 8 rRNA genes, and one pseudogene (Table 2, Table S3, and Fig. 2). Of particular interest, the inversion of the *trn*Y*-trn*D*-trn*E gene, previously observed in certain species of *Angelica* L. and *Peucedanum* L., was also detected in *L. incana*. [44, 53]. Additionally, these thirteen *Libanotis* plastomes exhibited no gene rearrangements or losses (Fig. 3). Additionally, a total of 1049 simple sequence repeats (SSRs) and 549 repeats belonging to four different types were identified (Fig. S1, Table S6, S7). Compared with other related taxa of Selineae, such as *Seseli* and *Peucedanum*, *Libanotis* was not much difference in the analysis of repeated sequences.

**Table 2:** Features of the twelve *Libanotis* plastomes newly sequenced

Taxa	Total length (bp)				GC content (%)				Gene numbers			
	Size	LSC	SSC	IR	Total	LSC	SSC	IR	Total	Protein-coding genes	tRNA	rRNA
***L. sibirica***	146,836	92,353	17,183	18,650	37.6	36.0	31.3	44.5	129	84	36	8
***L. seseloides***	147,950	93,067	17,187	18,848	37.6	36.0	31.0	44.4	129	84	36	8
***L. schrenkiana***	146,960	92,387	17,189	18,692	37.6	36.1	31.3	44.4	129	84	36	8
***L. buchtormensis***	148,048	93,128	17,204	18,858	37.6	36.0	31.1	44.4	129	84	36	8
***L. iliensis***	147,795	93,126	17,395	18,637	37.6	36.0	31.0	44.6	129	84	36	8
***L. grubovii***	147,471	93,234	17,401	18,418	37.6	36.0	31.0	44.8	129	84	36	8
***L. incana***	147,273	92,849	17,248	18,588	37.6	36.0	31.2	44.6	129	84	36	8
***L. depressa***	148,100	91,631	17,595	19,437	37.4	36.0	31.0	43.7	129	84	36	8
***L. lanzhouensis***	147,742	93,399	17,609	18,367	37.6	36.0	31.0	44.7	129	84	36	8
***L. jinanensis***	147,488	93,057	17,653	18,389	37.5	35.9	31.0	44.7	129	84	36	8
***L. condensata***	147,763	93,538	17,669	18,278	37.5	35.9	31.0	44.8	129	84	36	8
***L. acaulis***	147,829	93,157	17,670	18,501	37.5	35.9	30.9	44.6	129	84	36	8

### Nucleotide diversity analyses and potential DNA barcodes

**Fig. 4:**
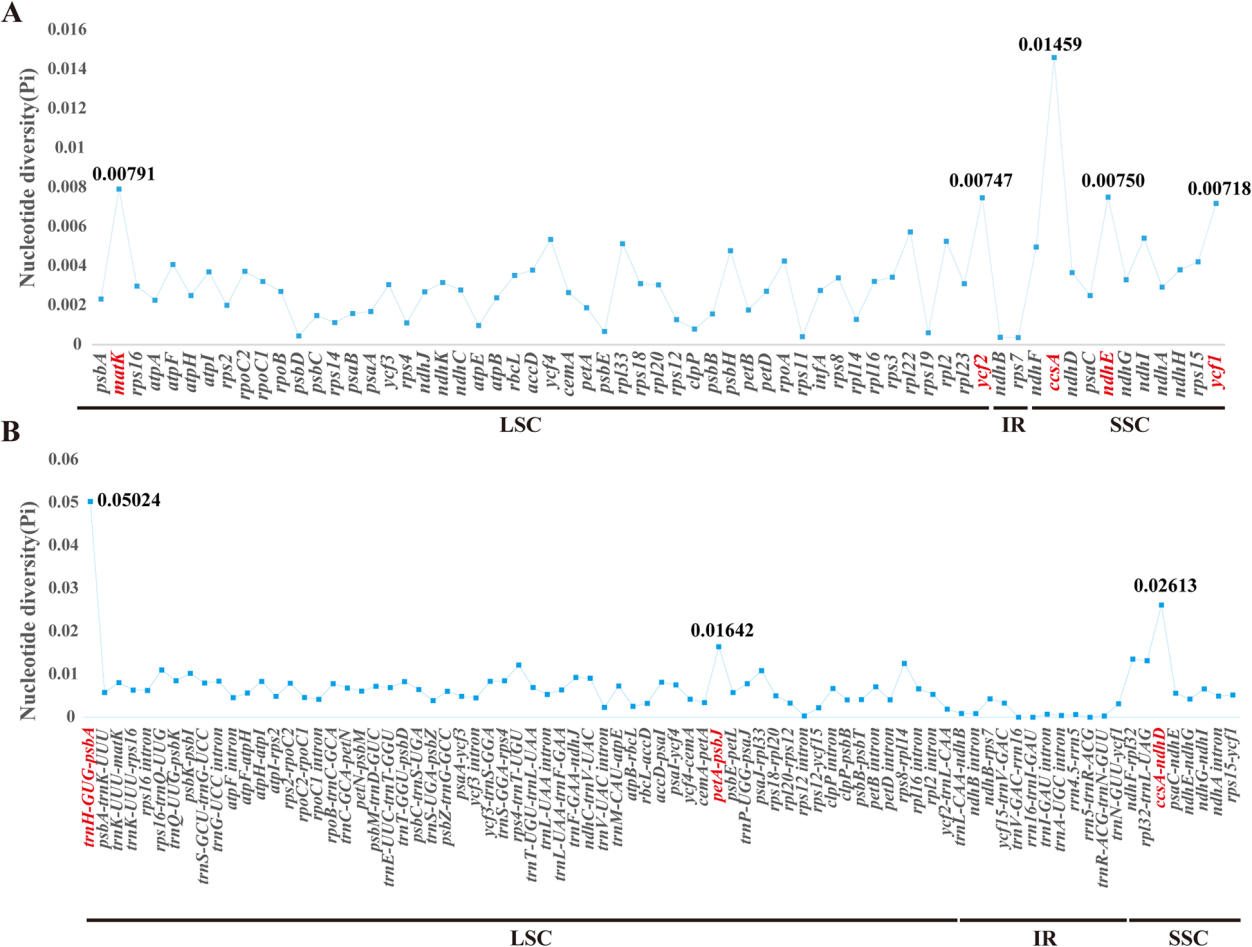
Assessing nucleotide diversity (Pi) across the thirteen *Libanotis* plastomes through comparative analysis: (**A**) protein-coding genes, (**B**) non-coding and intron regions

For these thirteen *Libanotis*, the nucleotide diversity (Pi) values for the protein-coding regions ranged from 3.58 × 10^–4^ (*rps*7 gene) to 0.01459 (*ccs*A gene), and the average value was 3.23 × 10^–3^ (Fig. 4, Table S8). The range of Pi values in non-coding regions and introns exhibits a considerable variation compared to coding regions. Among the protein-coding genes analyzed, only *ccs*A displayed a relatively high Pi value (> 0.01), whereas four other genes, namely *mat*K, *ycf*2, *ndh*E, and *ycf*1, exhibited moderate levels of nucleotide diversity (0.007 < Pi < 0.01), making them viable alternatives for further investigation (Fig. 4A, Table S8). Furthermore, three non-coding regions and introns with high nucleotide diversity (Pi > 0 0.015) were identified: *trn*H*-*GUG*-psb*A, *pet*A*-psb*J, and *ccs*A*-ndh*D (Fig. 4B, Table S8). These eight highly variable regions (*ccs*A, *mat*K, *ycf*2, *ndh*E, *ycf*1, *trn*H*-*GUG*-psb*A, *pet*A*-psb*J, and *ccs*A*-ndh*D) were selected as potential DNA barcodes.

### Phylogenetic analyses

**Fig. 5:**
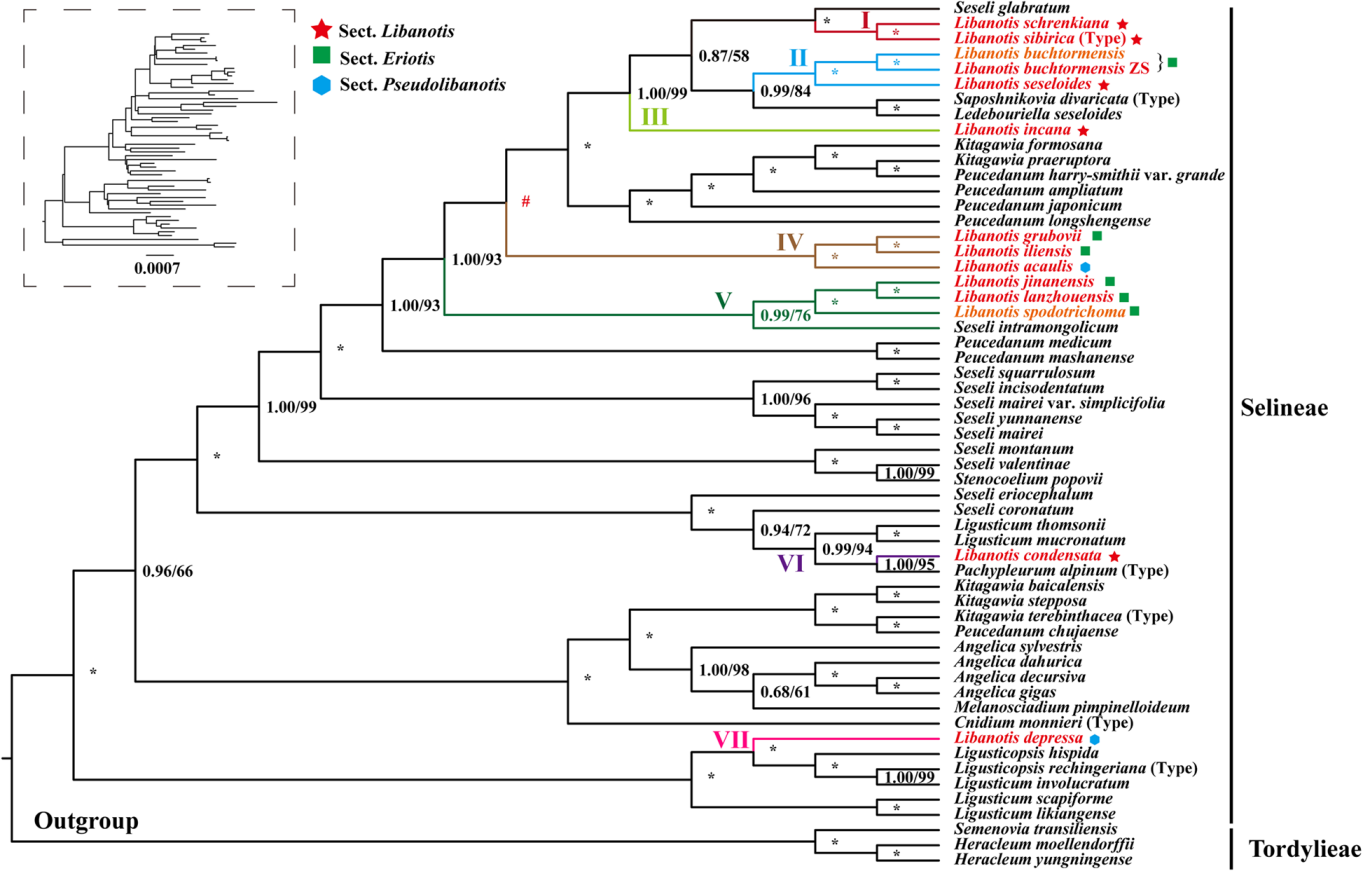
The plastome CDS-based phylogenetic tree constructed by Bayesian inference (BI) and maximum likelihood (ML) with the posterior probabilities (PP) of BI and the bootstrap values (BS) of ML above the branches. The topology of the tree is derived from the optimal tree of the maximum likelihood method, and the unaligned tree is labeled in the upper left corner. Respectively, (*) represents maximum support in both two analyses, (#) represents those nodes not occurring in the BI strict consensus tree. The red is the newly sequenced *Libanotis* in this study, and the orange is the *Libanotis* sequences downloaded from Genebank. Different subclades are colored differently. Details of the sections labeled with different symbols are shown in Table 1

**Fig. 6:**
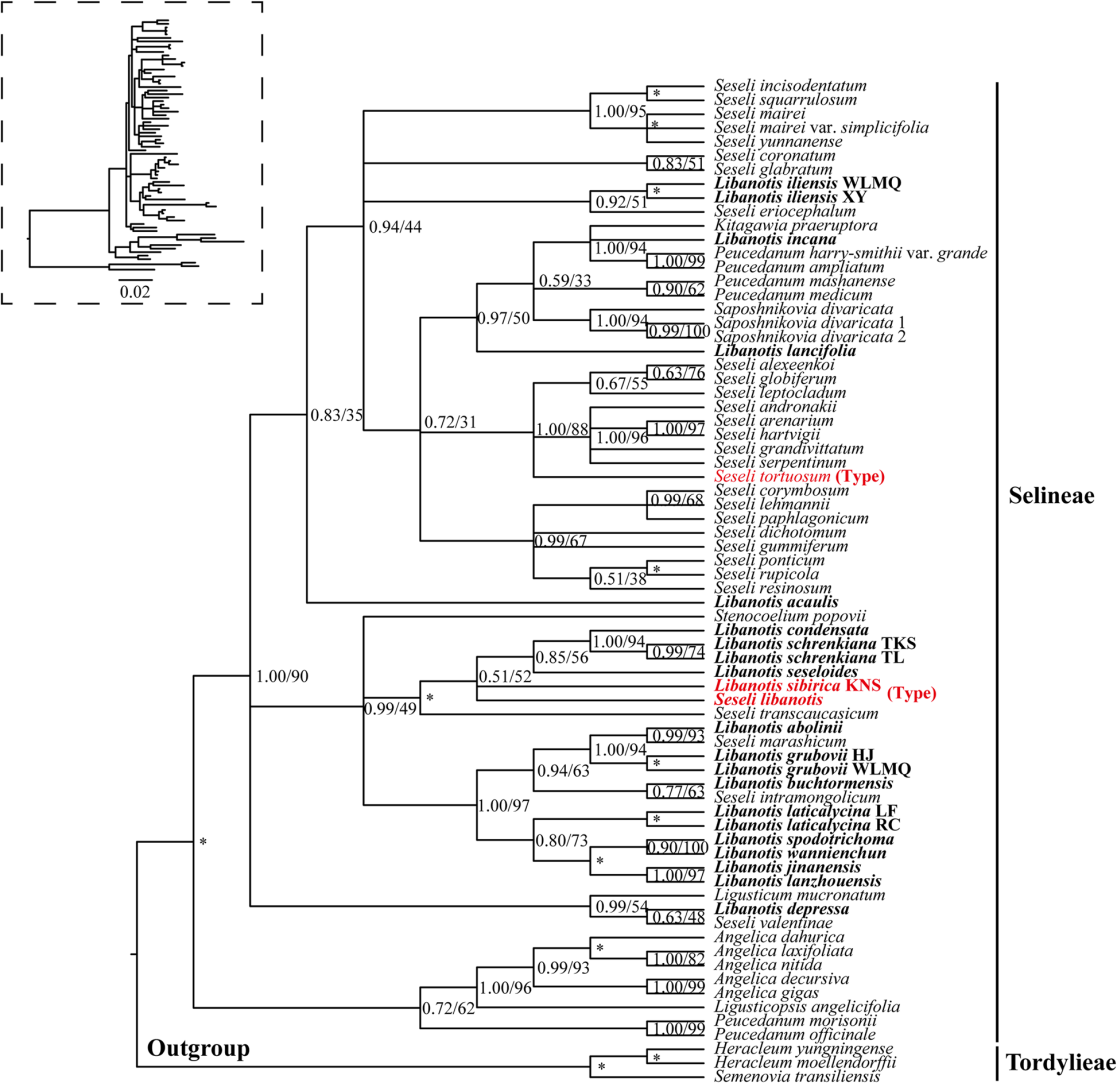
The plastome nrDNA-based (ITS + ETS) phylogenetic tree constructed by Bayesian inference (BI) and maximum likelihood (ML) with the posterior probabilities (PP) of BI and the bootstrap values (BS) of ML above the branches. The topology of the tree is derived from the optimal tree of the bayesian inference method, and the unaligned tree is labeled in the upper left corner. (*) represents maximum support in both two analyses. Bolded are the sequences newly sequenced in this study, and the type species of *Seseli* and *Libanotis* are highlighted in red in the figure

Seventy-nine single-copy plastome CDS from 57 plastomes were used to reconstruct the phylogeny of *Libanotis* (Fig. 5, Table S4). Our analyses robustly supported that the *Libanotis* taxa fell into one tribe (Selineae), and they were not clustered as a monophyletic group or divided into three sections but fell into seven groups (Subclades) (Fig. 5): (I) *L. sibirica* and *L. schrenkiana* clustered with *Seseli glabratum* Willd. ex Schult. (PP = 1.00, BS = 100); (II) *L. buchtormensis* and *L. seseloides* was sister to *Saposhnikovia divaricata* (Turcz.) Schischk. (PP = 0.99, BS = 84); (III) *L. incana* did not clustered with other *Libanotis*. However, within the phylogenetic analysis, *L. incana* and subclades I and II formed a robust clade with high support values (PP = 1.00, BS = 100). This clade indicated that *L. incana* diverged first from the rest of the taxa (PP = 1.00, BS = 99); (IV) *L. iliensis*, *L. grubovii* and *L. acaulis* formed a clade (PP = 1.00, BS = 100), clustered with I, II, III, and some *Peucedanum* species. (V) this clade contained *L. jinanensis*, *L. lanzhouensis*, *L. spodotrichoma*, and *Seseli intramongolicum* Y. C. Ma. (PP = 0.99, BS = 76); (VI) *L. condensata* was sister to *Pachypleurum alpinum* (PP = 1.00, BS = 95), and *P. alpinum* is type of *Pachypleurum*; (VII) *L. depressa*, along with other *Ligusticopsis* species, established a strong and clearly separated clade, displaying high support values (PP = 1.00, BS = 100), distinguishing it from the rest of the genus. In the nrDNA-based tree (Fig. 6, Table S5), that species in the subclades were clearly divergent except for Subclade V, which was better clustered into a single branch (PP = 1.00, BS = 100). *L. sibirica* and *S. libanotis* were clearly not clustered with *Seseli* s.s., *S. tortuosum* and some *Seseli* species were clustered with *Kitagawia*, *Peucedanum*, *L. incana*, and *L. lancifolia*, while *L. sibirica*, *S. libanotis* and some *Libanotis* were clustered with *Stenocoelium popovii*, and several *Seseli* species.

**Fig. 7:**
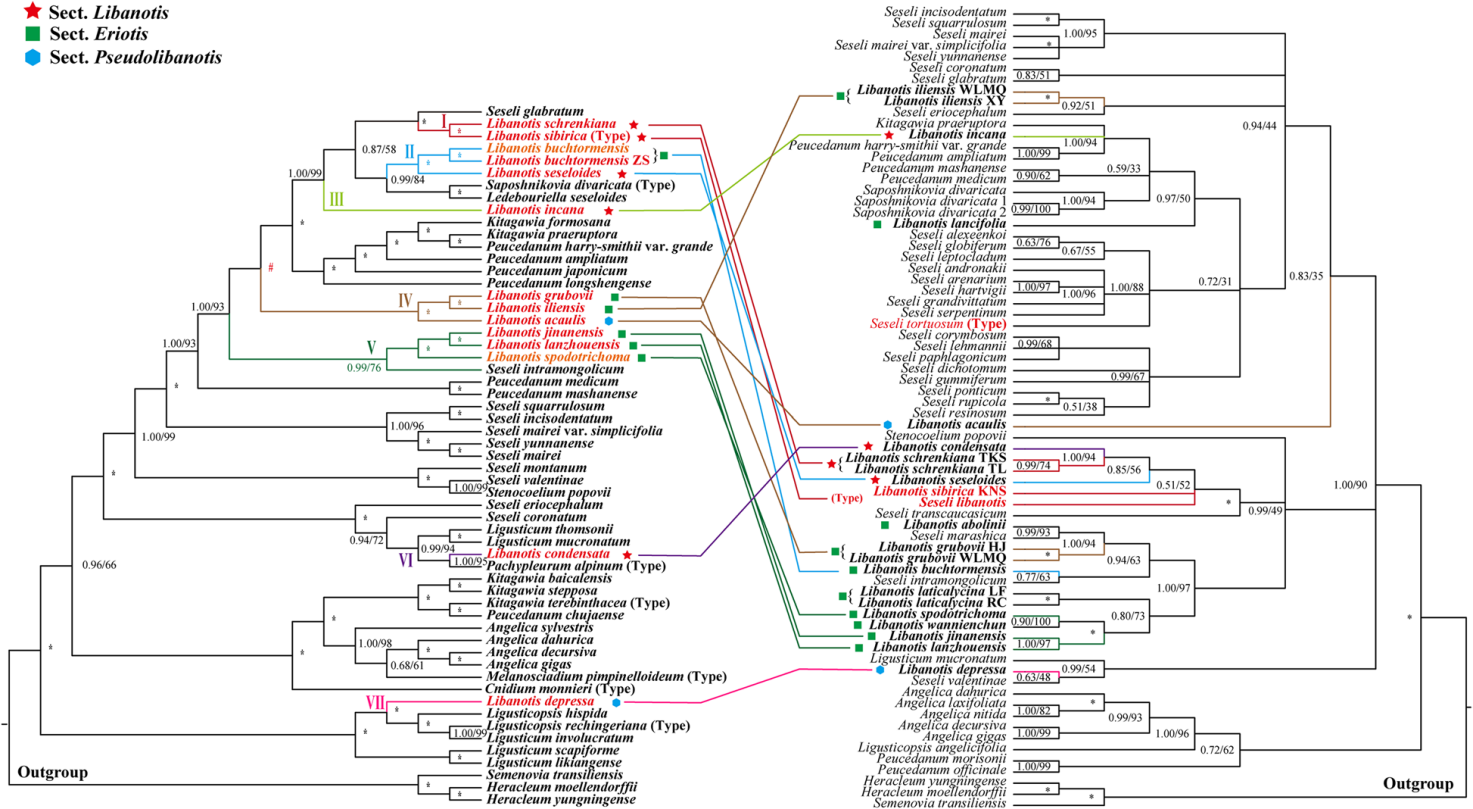
Comparison of two trees constructed based on different datasets. The left one is a CDS-based phylogenetic tree, and the right one is an nrDNA-based (ITS + ETS) phylogenetic tree. The same species with different subclades in the two trees are connected to each other by a line representing the color of that subclade. Details of the sections labeled with different symbols are shown in Table 1

In terms of morphological sections of CDS-based tree, only *L. sibirica* and *L. schrenkiana* (Subclade I) can be retained from the five species of the core group Sect. *Libanotis*, with the remaining three species each dispersed in three other branches (Subclade II, III, VI). The six species of Sect. *Eriotis* are also not monophyletic, with the exception of *L. buchtormensis* which is better concentrated in two branches (Subclade IV, V), and the two species of Sect. *Pseudolibanotis* are also separated, one clustered with Sect. *Eriotis* and one within the genus *Ligusticopsis* (Subclade IV, VII). On the nrDNA-based tree (Fig. 7), Sect. *Libanotis* except *L. incana* clustered together. It is noteworthy that these species of *Libanotis* sect. *Eulibanotis* included in Schischk [14] (Table 1) included in this tree (*L. montana* (≡ *S. libanotis*), *L. sibirica*, *L. schrenkiana*, *L. condensata*, *L. seseloides*, *L. transcaucasica* (≡ *S. transcaucasicum*)) clustered into a highly supported monophyletic clade (PP = 1.00, BS = 100). Sect. *Eriotis* apart from *L. iliensis* and *L. lancifolia* also clustered into a monophyletic clade (PP = 1.00, BS = 97), but within this clade were also included two narrowly-fielded *Seseli* species published in recent years. The two species of Sect. *Pseudolibanotis* are also separated.

### Comparative plastome analyses

The boundaries of these species were not too distinctly different or regular, either on the basis of phylogenetic subclades or on the basis of former taxonomic sections (Fig. S2). There is no doubt that the plastome structure of *Libanotis* is relatively conserved. The Relative Synonymous Codon Usage (RSCU) values across all codons exhibited a spectrum from 0.33 to 2.02, as depicted in Figure S3 and detailed in Table S9. Notably, *L. depressa* (Subclade VII) exhibited lower RSCU values for UGA termination codon (RSCU = 0.57), whereas *L. condensata* (Subclade VI) showed lower RSCU values for UAG termination codon (RSCU = 0.62) and higher values for UGA termination codon (RSCU = 0.74) compared to other subclades. The usage of specific codons within the remaining subclades, apart from the aforementioned individual subclades, shows no significant differences. (Fig. S3; Table S9).

**Fig. 8:**
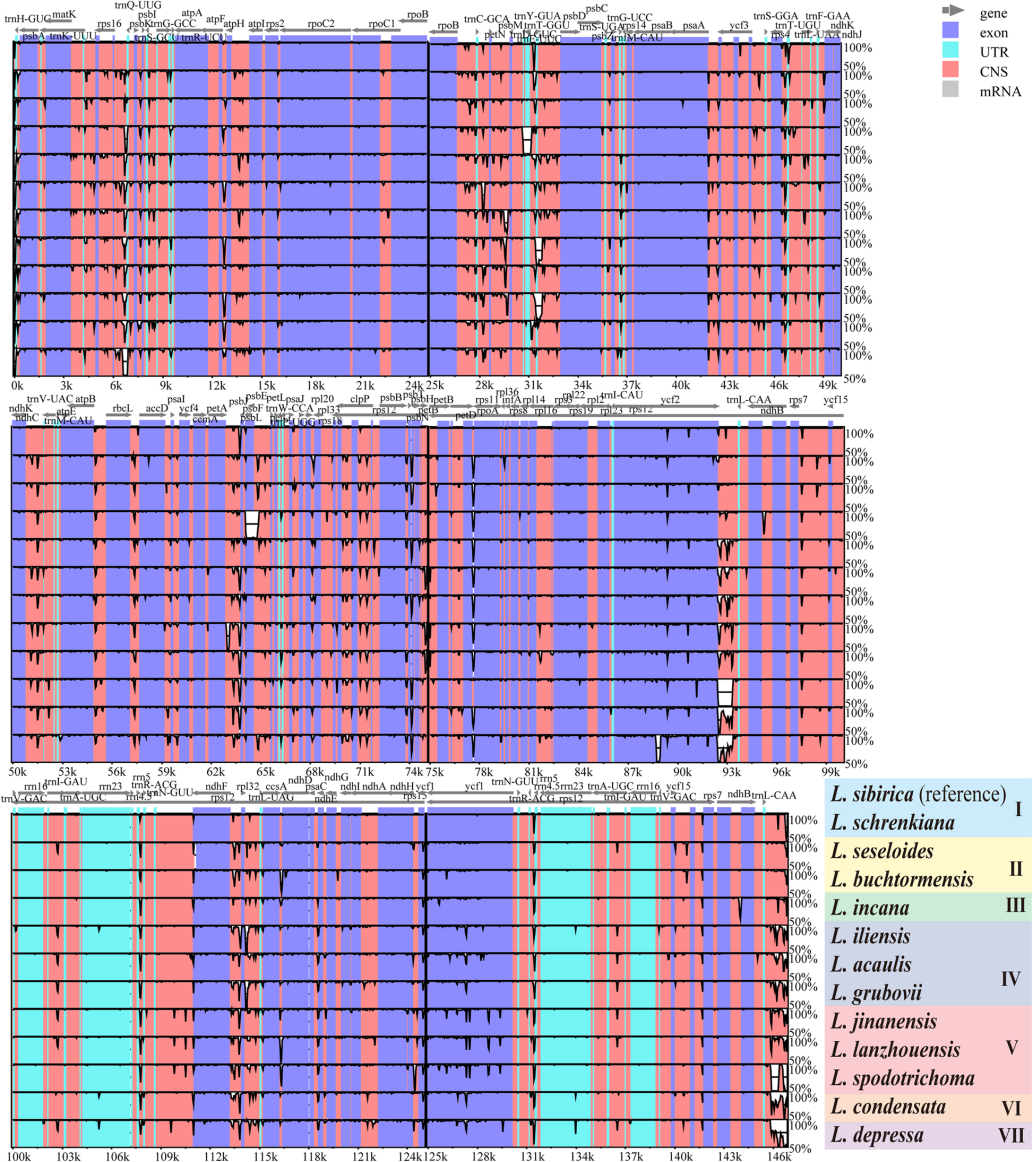
mVISTA-based sequence identity plots for the thirteen plastomes with *L. sibirica* as the reference. The different colors and Roman numerals correspond to the different subclades separated by the plastome CDS-based phylogenetic tree in Fig. 5

The divergence analysis of thirteen *Libanotis* plastomes revealed that the coding regions exhibited higher conservation compared to the non-coding regions (Fig. 8). Compared with other taxa, *L. schrenkiana* was highly similar to the reference *L. sibirica*. Furthermore, the plastid divergence between Subclades I and II is relatively low, as is the divergence between IV and V, while the remaining three separate subclades exhibit distinct differences (Fig. 8). Interestingly, some subclades exhibit a certain degree of conservation when compared to the rest of the subclades, while others show significant differences. For instance, *L. incana* (Subclades III) displays significant distinctions from the rest of the sequences in the region from *trn*D*-*GUC to *trn*E*-*UUC, likely due to gene inversion, which aligns with the above analysis. It also exhibits noticeable differences from other sequences in the region from *psb*L-*psb*F-*psb*E.

## Discussion

### Comparison of the *Libanotis* plastomes and Potential DNA barcodes

In this study, we sequenced and assembled twelve plastomes of *Libanotis* and performed comprehensive comparative analyses of these plastomes with one other published plastomes of this genus obtained from GeneBank. All *Libanotis* plastomes exhibited the typical quadripartite structure with various features displaying similarity. And the genome length (146,836 BP- 148,100 bp), IR/ SC borders and gene numbers and arrangements (129) of each Subclades formed by *Libanotis* species were not significantly different. These results suggested that *Libanotis* plastomes were highly conserved between different subclades, while the coding regions were more conserved than the non-coding regions, the IR regions were more conserved than the single copy regions. Nevertheless, we identified eight mutation hotspot regions, each spanning over 200 bp, with elevated Pi values. These regions, including the *mat*K gene, *ycf*2 gene, *ccs*A gene, *ndh*E gene, *ycf*1 gene, *trn*H*-*GUG*-psb*A, *pet*A*-psb*J, and *ccs*A*-ndh*D, were selected as potential DNA barcodes for the purposes of phylogenetic analysis and species identification within the *Libanotis* genus.

### Phylogeny analyses and taxonomic inference

We have reconstructed the phylogenetic relationships using 13 *Libanotis* species plastomes sample (Fig. 5). This work provides a solid and high-resolution phylogenetic tree of *Libanotis*, revealing inconsistencies between molecular systematics and traditional taxonomic studies. According to the current research results, the genus *Libanotis* is obviously polyphyletic, and *L. sibirica* (type species) and *L. schrenkiana* (Subclade I) formed a monophyletic clade with strong supports. Meanwhile, the clade could be recognized by leaf segments ovate-rhombic or lanceolate, surfaces glaucous on the back of the leaves and sparsely puberulent; bracts absent or few, subulate to linear, small, easy to loss; bracteoles several, linear; petals abaxially glabrous; calyx teeth conspicuous, triangular-lanceolate; fruit ovoid-ellipsoid, dorsally compressed, densely pubescent when young, becoming sparsely puberulent or glabrous; ribs subequal, shortly keeled; vittae 1 in each furrow, 2 on commissure [1, 2]. As Pimenov argues [22], *Libanotis* s.s. and *Seseli* s.s. do not differ in fruit morphology up to the genus level, and their main morphological differences are in less commonly used morphologies (characteristics of bracteoles, bracts and leaf segments, stem branching, stem and petiole pubescent etc.). But the results of the phylogeny suggest that we cannot simply merge them because the monophyly of the genera is not supported, and we cannot rule out the effects of homoplasy or reversals. Just like the concept of cryptic species, *Libanotis* is in a sense a cryptic genus. Therefore, we propose to accept this genus in narrow sense, namely *Libanotis* s.s., and identify only two members for the time being. According to the type specimens and literature records, other possible members of *Libanotis* s.s. are *Seseli junatovii* V. M. Vinogradova and *Seseli salsugineum* A.Duran & Lyskov. However, due to the limited sampling and the lack of sufficient reliable morphological information, we would not make taxonomic treatments for now. The results of the comparison between nrDNA-based tree and the CDS-based tree (Fig. 7) showed that there was nucleoplasmic conflict in *Libanotis*, these may be due to incomplete lineage sorting and introgression. In these conflicts, we found that species with similar leaf morphology tended to cluster more in the nrDNA-based tree: in the branch where *L. sibirica* located, the four *Libanotis* species (*L. sibirica*, *L. schrenkiana*, *L. seseloides*, and *L. condensata*) all have green, thin, papery leaf blades, and leaf abaxial surfaces sometimes gray-green; whereas the eight *Libanotis* species (*L. abolinii*, *L. grubovii*, *L. buchtormensis*, *L. laticalycina*, *L. spodotrichoma*, *L. wannienchun*, *L. jinanensis*, and *L. lanzhouensis*) in the branch beneath them have leathery to fleshy leaf blades, the leaf blades mostly blue-green or gray-green overall. The rest of the dispersed species have distinctive vegetative body morphology. Meanwhile, species with similar morphology of mericarps tended to cluster together (such as subclades I and V) in the CDS-based tree (Table S10).

When considering the outcomes of morphological sections within the phylogenetic tree, it becomes evident that the alignment is less than ideal. None of the three sections of taxa appear to be monophyletic. This situation is not unique among Apiaceae family. The Apiaceae, located on the upper echelons of angiosperms, signify a taxon along the path of divergence, and belong to the most complicated families of flowering plants, also in terms of species identification [54–56]. The reliability of diagnostic features between and within genera may be affected by homoplasy and reversals, and that traditional printed dichotomous keys in large “Floras” are far from satisfying. Recent times have witnessed a rapid reconfiguration of species within the tribe Selineae, marked by the revision of established genera and the independence of new ones [57–59]. In our assessment, we propose that all thirteen species in this study, except *L. sibirica* and *L. schrenkiana*, should be transferred and revised, but not transferred to *Seseli* to further confuse the polygenetic genus. It is worth noting that *Peucedanum*, *Saposhnikovia* and *Kitagawia*, which are close relatives of *Libanotis* and *Seseli*, also suffer from the problems mentioned above. Comprehensive sampling of *Seseli* and *Peucedanum*, two the world-wide complex genera with a mass of species, will be crucial to the taxonomic system of *Libanotis* and the entire tribe Selineae.

Except for them, the members of *Libanotis* were scattered among the branches, and the phylogenetic positions of *L. condensata* and *L. depressa* are particularly noteworthy. *L. condensata* (Subclade VI) and *Pachypleurum alpinum* Ledeb. (Type species of *Pachypleurum*) clustered together. Their morphology is similar in vegetative body which both have solitary stem with branched above or simple, hollow, glabrous, and striate, base densely clothed with fibrous leaf remains, and oblong leaf blade, but quite different in mericarps especially ribs all winged, subequal in *Pachypleurum*. Thus, *L. condensata* may be more closely related to *Pachypleurum* than *Seseli* s.s. or *Libanotis* s.s., but its transfer to *Pachypleurum* seems inappropriate unless the definition of *Pachypleurum* is reconstructed. Other molecular evidence [7] also supports the view that *L. condensata* does not belong to *Libanotis*. In nrDNA sequence (ITS) phylogenetic results [7], *L. condensata* is obviously separated from above genera, and is located in *Pilopleura* Schischk. While our nrDNA tree (ITS + ETS) showed that *L. condensata* was inserted into *Libanotis* s.s.. Unfortunately, due to the chloroplast genome and ETS sequence of *Pilopleura* absented, we could not confirm the relationship between *Pilopleura* and *L. condensata*. *L. depressa* clustered with *Ligusticopsis* species. However, we found *L. depressa* develops few bracts, lanceolate and very unequal bracteoles, and mericarps with few vittae in the furrow (1) and commissure (2), not strongly compressed and marginal ribs not winged, which are distinguishable from *Ligusticopsis*. Consequently, *L. depressa* should be treated as an independent taxon distinct from *Ligusticopsis* or *Libanotis*. Pimenov [24] argued that *L. depressa* should be transferred to *Stenocoelium*. Our results showed that *Stenocoelium popovii* clustered with some *Seseli* species and was far away from *L. depressa*. *L. depressa* also does not conform to the unique mericarp morphology of *Stenocoelium* that ribs are thick-obtuse, very prominent, irregularly denticulate especially along ribs and furrows are narrow. Due to conflicting and partial lack of morphological data sampling, we will detailedly discuss its taxonomic status in future research. The other species of *Libanotis* (Subclade II, III, IV, V) were clustered into some relatively single clades: *L. incana* (Subclade III) was alone; *L. seseloides* and *L. buchtormensis* were gathered in one branch (Subclade II) and then sister to *Saposhnikovia*, but the shape of mericarps and the numbers of vittae are quite different among them; *L. acaulis* and *L. grubovii*, *L. iliensis* clustered together (Subclade IV); *L. lanzhouensis*, *L. jinanensis*, and *L. spodotrichoma* formed a clade (Subclade V). Compared with *Libanotis* s.s., they belong to Sect. *Eriotis* or Sect. *Pseudolibanotis*, and petals are densely coated with soft hairs or stem not developed, which is easy to distinguish. In conclusion, our results showed that *Libanotis* s.s. has a need to be retained, but other eleven species that thought to be attributed to *Libanotis* should be transferred out *Libanotis* genus but their taxonomic status needs to be further studied by adding more species.

## Conclusion

This study marks the inaugural endeavor to conduct a comprehensive exploration of plastome characteristics and to deduce the phylogeny of the *Libanotis* genus, encompassing a total of thirteen *Libanotis* species. In the course of this investigation, we conducted the fresh sequencing, assembly, and annotation of complete plastomes for twelve *Libanotis* species along with two closely related taxa. These results suggested that *Libanotis* plastomes were conserved between different subclades, while the coding regions were more conserved than the non-coding regions, and the IR regions were more conserved than the single copy regions. Nevertheless, eight mutation hotspot regions (*mat*K gene, *ycf*2 gene, *ccs*A gene, *ndh*E gene, *ycf*1 gene, *trn*H*-*GUG -*psb*A, *pet*A*-psb*J, *ccs*A*-ndh*D) longer than 200 bp with high Pi values were chosen as potential DNA barcodes for the purpose of both phylogenetic investigation and species identification used in materia medica of *Libanotis*. 78 common single-copy CDS from fifty-seven plastomes sequences and 144 nrDNA (72 ETS + 72 ITS) sequences were used to perform the phylogenetic analysis of *Libanotis*. Plastid phylogenomic analyses confirmed the efficacy of plastome data in enhancing the support and resolution of *Libanotis* phylogeny, firmly showing that *Libanotis* belong to Selineae and not a monophyletic genus, and the species within the sections in the original morphological framework are also polyphyletic. We finished the delimitation of *Libanotis* by establishing *Libanotis* s.s. and provided some taxonomic suggestions for other species in the genus, especially *L. depressa* and *L. condensata*. In short, our study can provide new insights into the plastome evolution of *Libanotis* and promoted the improvement of taxonomic system for Aipaceae family.

## Methods

### Taxon sampling and DNA sequencing

A total of 57 plastomes from 56 taxa and 144 nrDNA sequences (72 ITS + 72 ETS) from 67 taxa were used in this study, of which 48 plastomes and 96 nrDNA originated from us (Table S4, S5). We collected fresh and fully developed leaves from twelve different *Libanotis* species, which included the type species *L. sibirica* (L.) C.A.Mey. and then dried with silica gel (Table S1). These sections of *Libanotis* species reference FRPS [2], including all three sections (one previous section has been used to create the new monotypic genus *Sajanella*) to establish a more complete phylogenetic framework (Table 1, Fig. 1). Additionally, we expanded our sampling efforts for *Pachypleurum alpinum* Ledeb. and *Stenocoelium popovii* V.M.Vinogr. & Fedor., based on prior experimentation and taxonomic studies [24, 60]. In addition to the 14 plastomes, we newly measured 24 ETSs as well as 24 ITSs containing 17 species of *Libanotis* and two closely related species (Table S2). The formal identifications of all collected samples were identified by Liu Li-Jia and Professor He Xing-Jin from Sichuan University. Specimens vouchering the mentioned taxa were stored in the herbarium of Sichuan University (SZ) and the herbarium of the Kunming Institute of Botany (KUN), and the details of these vouchers can be found in Table S1 and S2. In order to distinguish them from other genera, all the scientific names of *Libanotis* species and sections in this study were based on the taxonomic treatment of FOC and IPNI [1, 61], but the scientific names in Table 1 followed the authors' original records. The newly published species *L. jinanensis* and the newly transferred species *L. grubovii* are included in Sect. *Eriotis* according to their morphologic descriptions (Table 1, Table S10).

We began by extracting total DNA from approximately 20 ~ 30 mg of silica gel-dried leaves using the CTAB (Cetyl trimethylammonium bromide) method [62]. We conducted Polymerase chain reactions (PCRs) to amplify ITS (Internal Transcribed Spacer) and ETS (External Transcribed Spacer) sequences using the following primers: ITS-4, ITS-5 [63], 18S-ETS [64], and Umb-ETS [65]. Each PCR reaction had a 30 µL volume with 2 µL plant DNA, 1.5 µL forward primer,1.5 µL reverse primer, 15 µL of 2 × Taq MasterMix (cwbio, Beijing, China), and 10 µL of ddH2O. We used Geneious v2023.0.4 [66] for sequence editing and assembly. The newly acquired sequences have been officially submitted in GenBank (accession numbers in Table S2). For plastomes, we fragmented the genomic DNA into 150 bp fragments to create a pair-end library, adhering to the manufacturer's instructions provided by Illumina in San Diego, CA, USA. The sequencing of these libraries took place on the Illumina NovaSeq platform at Personalbio in Shanghai, China. We applied fastP v0.15.0 [67] to filter the raw data, and these high-quality reads were then assembled for the whole plastomes using GetOrganelle v1.7.7.0 [68].

### Genomic annotation and feature analyses

We utilized the Plastid Genome Annotator (PGA) [69] for the annotation of plastomes, employing *L. buchtormensis* (MZ707534) and *L. spodotrichoma* (MZ707535) as our reference sequences. Subsequently, we performed manual refinements using Geneious v2023.0.4 [66]. The newly acquired plastome sequences for the twelve *Libanotis* taxa, along with two additional sequences, have been officially submitted in GenBank (accession numbers in Table S1). To visualize the circular plastome maps for the twelve newly sequenced *Libanotis* taxa, we employed the online tool Organellar Genome DRAW (OGDRAW) [70]. Furthermore, we identified gene rearrangements among the thirteen *Libanotis* taxa including one previously published sequence, using Mauve Alignment [71] within Geneious v2023.0.4 [66].

### Repeat sequence and nucleotide diversity analyses

We employed the online REPuter program [72] to identify repeat sequences in the plastomes of the thirteen *Libanotis* taxa and the parameters used for this analysis referred to Cai et al. [33]. Furthermore, we utilized the Perl script MISA [73], available at http://pgrc.ipk-gatersleben.de/misa/sleben.de/misa/, to detect simple sequence repeats (SSRs) within the plastomes of the thirteen *Libanotis* taxa. For the assessment of nucleotide diversity (Pi) within protein-coding genes, noncoding regions, and introns, we turned to DnaSP version 6.12.03 [74]. This analysis aimed to pinpoint regions with elevated mutation rates, potentially serving as valuable molecular markers for future research. Regions meeting or exceeding a length of 200 base pairs were singled out for this purpose, as described previously [33].

### Sequences selection and alignment

In accordance with initial experiments and prior taxonomic assessments [24, 60], we carefully curated two dataset consisting of 57 complete plastomes derived from 56 taxa and 144 nrDNA (72 ITS + 72 ETS) from 67 taxa for the purpose of constructing phylogenetic trees. Notably, 13 plastomes of these sequences, which include 11 *Libanotis* species (including the type species *L. sibirica*) and two related taxa, *Stenocoelium popovii* and *Pachypleurum alpinum*, were being introduced for the first time into our analysis. And among these nrDNAs, all ETSs of 17 *Libanotis* and *Stenocoelium popovii* were sequenced for the first time. In recognition of the intricate relationship between *Seseli* and *Libanotis*, we incorporated *Seseli* into our study. To establish the root of our phylogenetic tree, we selected three species from the Tordylieae tribe: *Heracleum moellendorffii* Hance, *Heracleum yungningense* Hand.-Mazz., and *Semenovia transiliensis* Regel & Herder, as recommended by Wen et al. [75]. Our main clade designations were based on the contributions of Downie et al. [76] and Wen et al. [75]. We further assembled a dataset comprising 78 common single-copy coding sequences (CDSs) extracted from the 57 complete plastomes. This dataset was concatenated using PhyloSuite v1.2.2 [77]. To ensure accuracy, we aligned the sequences using MAFFT v7.221 [78] and performed inspection and manual refinements with the assistance of MEGA7 [79]. It's worth noting that all sequences data utilized in our phylogenetic analyses are readily accessible in GenBank (Table S4, S5).

### Phylogenetic analyses

To elucidate phylogenetic relationships, we employed both maximum likelihood (ML) and Bayesian inference (BI) methods. For ML analyses, we utilized RAxML v8.2.10 [80] with the GTRGAMMA model, accompanied by 1000 rapid bootstrap replicates to assess node support. In the case of BI analyses, we first determined the best-fitting substitution model using MrModeltest v2.4 [81]. Subsequently, we conducted Bayesian inference with MrBayes v3.2.7 [82], employing the selected GTR + I + G parameters. The parameter settings for the BI analysis refer to previous research about Apiaceae [33, 83, 84]. Finally, we visualized and edited the resulting phylogenetic trees using FigTree v1.4 [85].

### Comparative analyses of plastomes

We visualized the variations in size between the inverted repeat (IR) border regions in the plastomes of the thirteen *Libanotis* species using IRscope [86]. Any necessary manual adjustments were made to ensure accuracy. Subsequently, we conducted a sequence divergence analysis of these thirteen plastomes, using mVISTA [87] in Shuffle-LAGAN mode, with *L. sibirica* serving as the reference species. For codon usage analysis, we employed codonW [88]. To reduce the impact of sampling bias [63, 89], we selected 53 coding sequences (CDSs) from the thirteen plastomes, excluding CDSs shorter than 300 base pairs and repetitive sequences. These selected CDSs were then concatenated using PhyloSuite v1.2.2 [77]. To visualize the relative synonymous codon usage (RSCU) [90] values across the thirteen plastomes, we utilized TBtools [91].

### Supplementary Information

**Additional file 1: Fig. S1. **Analyses of repeats in the thirteen *Libanotis* plastomes. (A, B) Total number of SSRs,(C) Total number of four repeat types.See Table S6, S7 for specific values. **Fig. S2. **Comparing LSC, SSC, and IR region boundaries among the thirteen *Libanotis* plastomes, with gene positions indicated by different boxes. **Fig.S3. **The relative synonymous codon usage (RSCU) values of 53 CDSs for 13*Libanotis* plastomes. (*) to denote the terminator codons. See Table S9 for specific values. **Table S1. **The newly sequenced plastomes in the present study with taxa, source, voucher and GenBank accession numbers. **Table S2. **The newly sequenced nrDNA in the present study with taxa, source, voucher and GenBank accession numbers. **Table S3.** List of unique genes identified in plastomes of twelve *Libanotis* newly sequenced. **Table S4.** Plastomes included in phylogenetic analyses with GenBank accession and length. Bolded are newly sequenced sequences. (*) to denote the sequences from us. **Table S5.** nrDNA (ITS and ETS) included in phylogenetic analyses with GenBank accession. Bolded are newly sequenced sequences. (*) to denote the sequences from us. **Table S6.** Simple sequence repeats (SSRs) distribution in the thirteen *Libanotis* plastomes. These data were visualized in Figure S1. **Table S7.** The repeat sequences distribution in the thirteen *Libanotis* plastomes. These data were visualized in Figure S1. **Table S8.** Nucleotide diversity (Pi) values of thirteen *Libanotis*, while coding and non-coding regions were listed on the left and right, respectively. These data were visualized in Figure 4. **Table S9.** Codon usage and relative synonymous codon usage (RSCU) values of protein-coding genes of the thirteen plastomes. These data were visualized in Figure S3. **Table S10.** The morphological comparision of different *Libanotis* in this study. Data based on FOC (2005), JSTOR, CVH and sampled specimens.

## Data Availability

The fourteen newly sequenced plastomes have been submitted into NCBI with accession numbers: OR529367- OR529372, OR529374- OR529379, PP078851 and OQ685947, and details of the 48 newly sequenced ETS and ITS sequences are attached.

## Abbreviations

BI: Bayesian inference
bp: Base pair
BS: Bootstrap value
CDS: Coding sequences
CTAB: Cetyl trimethylammonium bromide
ETS: External transcribed Spacer
FRPS: Flora Republicae Popularis Sinicae
FOC: Flora of China
GC: Guanine-cytosine
IR: Inverted repeat
ITS: Internal transcribed spacer
KUN: Herbarium, Kunming Institute of Botany, Chinese Academy of Sciences
LSC: Large single copy
ML: Maximum Likelihood
PCGs: Protein-coding genes
PCR: Polymerase chain reaction
Pi: Nucleotide diversity
PP: Posterior probability
rRNA: Ribosomal RNA
RSCU: Relative synonymous codon usage
s.s.: Sensu stricto
SSC: Small single copy
SSR: Simple sequence repeat
SZ: Herbarium, College of Life Sciences, Sichuan University
tRNA: Transfer RNA
