# Supplementary material for: The complete plastomes of thirteen Libanotis (Apiaceae, Apioideae) plants: comparative and phylogenetic analyses provide insights into the plastome evolution and taxonomy of Libanotis
Source: BMC Plant Biol. 2024 Feb 12;24:106. doi: 10.1186/s12870-024-04784-4 (PMC10860227; doi:10.1186/s12870-024-04784-4)
Supplement: Supplementary file 1 — Additional file 1: Fig. S1. Analyses of repeats in the thirteen Libanotis plastomes. (A, B) Total number of SSRs,(C) Total number of four repeat types.See Table S6, S7 for specific values. Fig. S2. Comparing LSC, SSC, and IR region boundaries among the thirteen Libanotis plastomes, with gene positions indicated by different boxes. Fig.S3. The relative synonymous codon usage (RSCU) values of 53 CDSs for 13Libanotis plastomes. (*) to denote the terminator codons. See Table S9 for specific values. Table S1. The newly sequenced plastomes in the present study with taxa, source, voucher and GenBank accession numbers. Table S2. The newly sequenced nrDNA in the present study with taxa, source, voucher and GenBank accession numbers. Table S3. List of unique genes identified in plastomes of twelve Libanotis newly sequenced. Table S4. Plastomes included in phylogenetic analyses with GenBank accession and length. Bolded are newly sequenced sequences. (*) to denote the sequences from us. Table S5. nrDNA (ITS and ETS) included in phylogenetic analyses with GenBank accession. Bolded are newly sequenced sequences. (*) to denote the sequences from us. Table S6. Simple sequence repeats (SSRs) distribution in the thirteen Libanotis plastomes. These data were visualized in Figure S1. Table S7. The repeat sequences distribution in the thirteen Libanotis plastomes. These data were visualized in Figure S1. Table S8. Nucleotide diversity (Pi) values of thirteen Libanotis, while coding and non-coding regions were listed on the left and right, respectively. These data were visualized in Figure 4. Table S9. Codon usage and relative synonymous codon usage (RSCU) values of protein-coding genes of the thirteen plastomes. These data were visualized in Figure S3. Table S10. The morphological comparision of different Libanotis in this study. Data based on FOC (2005), JSTOR, CVH and sampled specimens. [file 12870_2024_4784_MOESM1_ESM.pdf]

Fig. S1 Analyses of repeats in the thirteen *Libanotis* plastomes. (A, B) Total number of SSRs, (C) Total number of four repeat types. See Table S6, S7 for specific values.

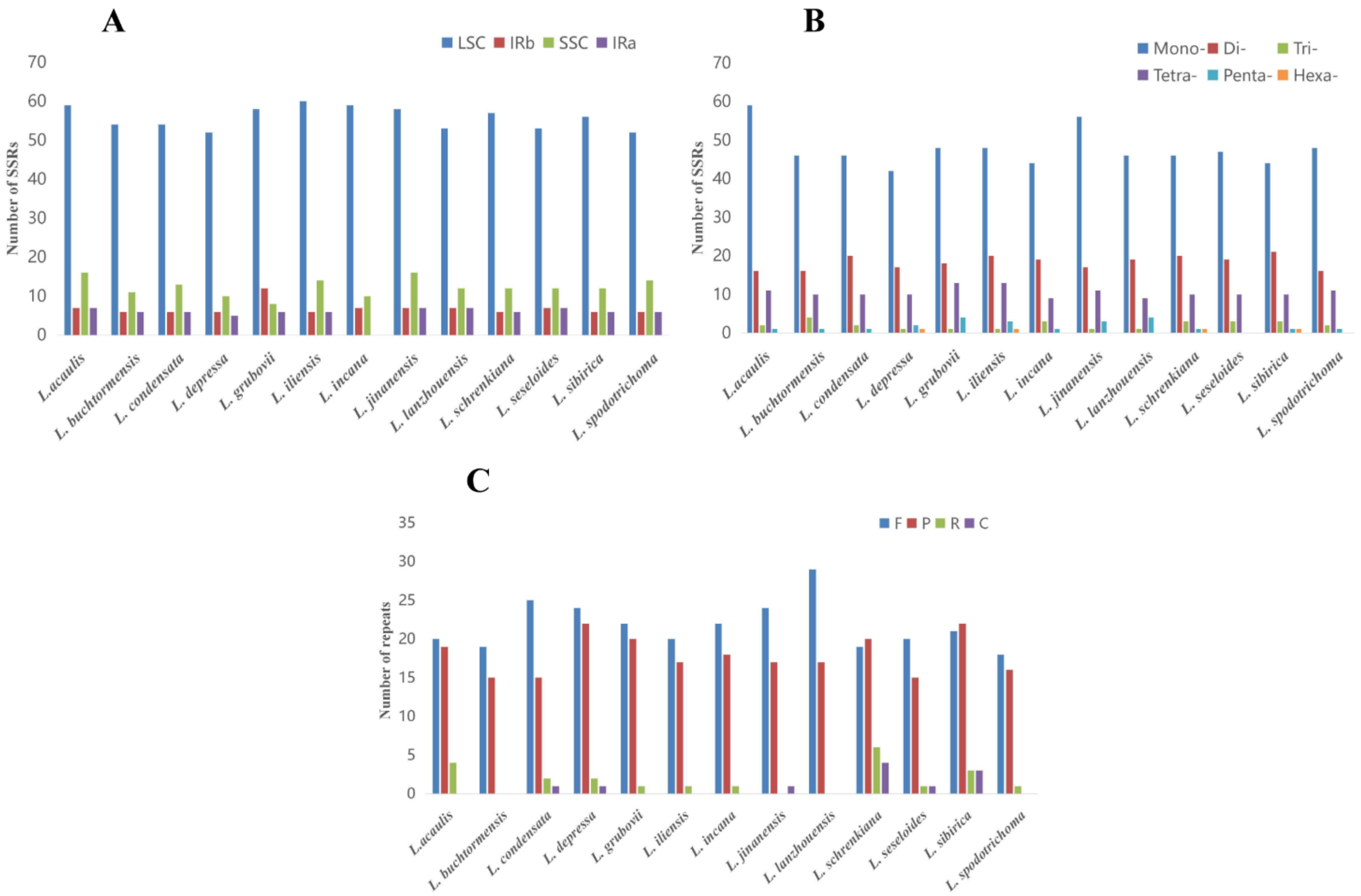

Fig. S2 Comparing LSC, SSC, and IR region boundaries among the thirteen *Libanotis* plastomes, with gene positions indicated by different boxes.

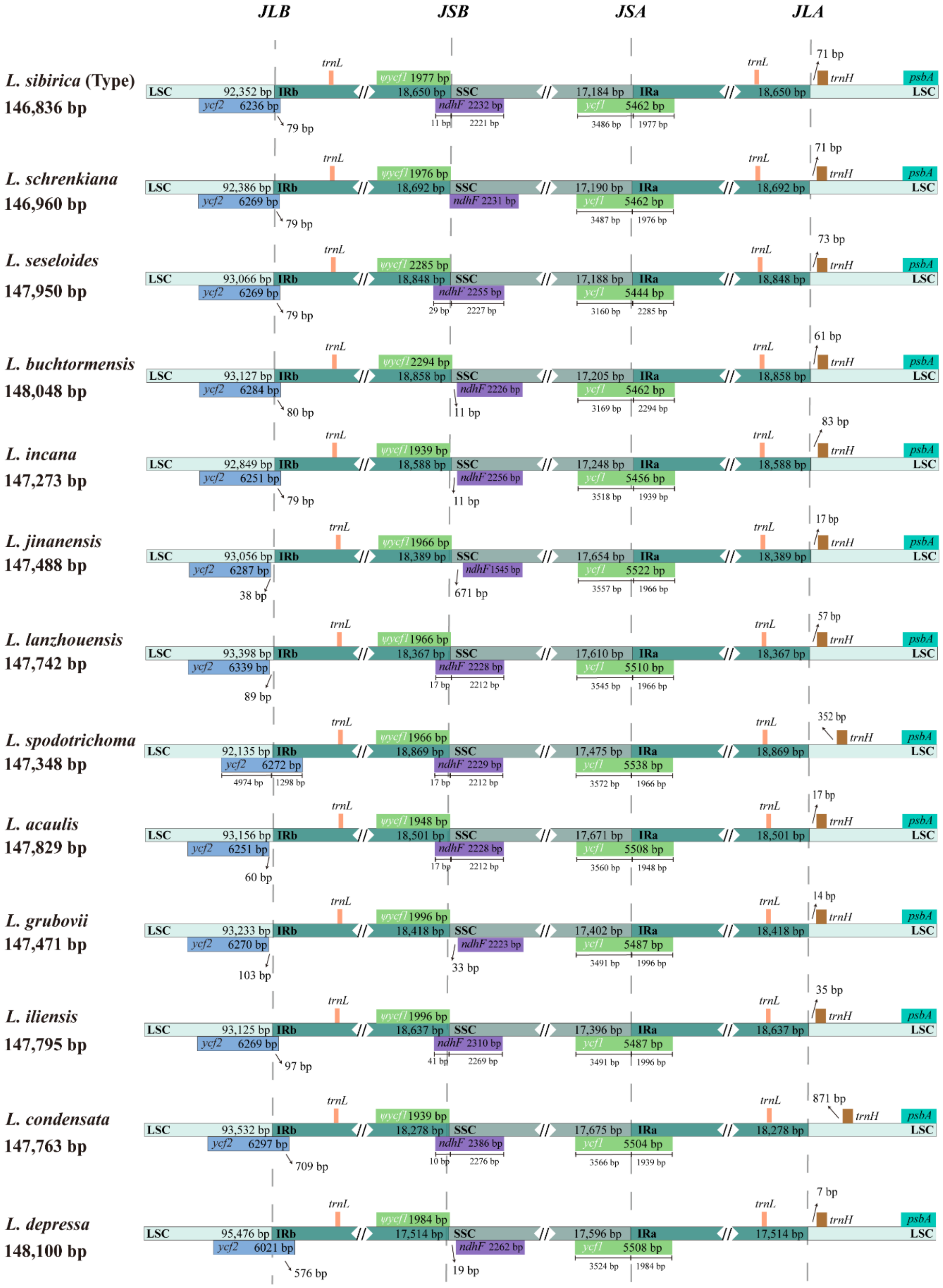

Fig. S3 The relative synonymous codon usage (RSCU) values of 53 CDSs for 13 *Libanotis* plastomes. (*) to denote the terminator codons. See Table S9 for specific values.

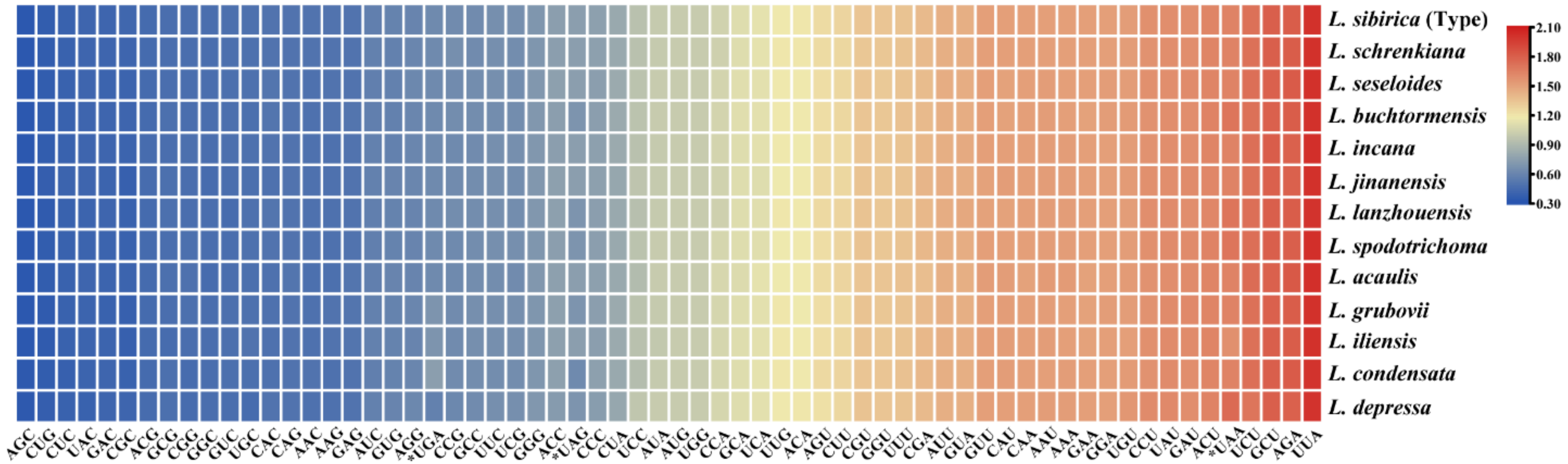

Table S1 The newly sequenced plastomes in the present study with taxa, source, voucher and GenBank accession numbers.

Taxa	Locality	Voucher information	Herbarium information	GenBank Accession
Libanotis acaulis	Hejing, Xinjiang, China	LLJ21070702-1	Herbarium, College of Life Sciences, Sichuan University (SZ)	**OR529367**
Libanotis buchtormensis	Zhaosu, Xinjiang, China	LLJ21070910-1	Herbarium, College of Life Sciences, Sichuan University (SZ)	**OR529368**
Libanotis condensata	Kanasi, Xinjiang, China	LLJ21071314-2	Herbarium, College of Life Sciences, Sichuan University (SZ)	**OR529369**
Libanotis depressa	Nangqian, Qinghai, China	PC201909249	Herbarium, College of Life Sciences, Sichuan University (SZ)	**OR529370**
Libanotis grubovii	Yiwu, Xinjiang, China	CJ20220607-1-2	Herbarium, College of Life Sciences, Sichuan University (SZ)	**OR529371**
Libanotis iliensis	Yili, Xinjiang, China	LLJ220800X	Herbarium, College of Life Sciences, Sichuan University (SZ)	**OR529372**
Libanotis incana	Tuoli, Xinjiang, China	CJ20220723-2-2	Herbarium, College of Life Sciences, Sichuan University (SZ)	**PP078851**
Libanotis jinanensis	Jinan, Shandong, China	LLJ221002-1	Herbarium, College of Life Sciences, Sichuan University (SZ)	**OR529374**
Libanotis lanzhouensis	Lanzhou, Gansu, China	LLJ21071918-2	Herbarium, College of Life Sciences, Sichuan University (SZ)	**OR529375**
Libanotis schrenkiana	Zhaosu, Xinjiang, China	LLJ21070909-5	Herbarium, College of Life Sciences, Sichuan University (SZ)	**OR529376**
Libanotis seseloides	Boli, Heilongjiang, China	LJQ211027006	Herbarium, College of Life Sciences, Sichuan University (SZ)	**OR529377**
Libanotis sibirica	Kanasi, Xinjiang, China	LLJ21071317-2	Herbarium, College of Life Sciences, Sichuan University (SZ)	**OR529378**
Pachypleurum alpinum	Fuyun, Xinjiang, China	17CS16477	Herbarium, Kunming Institute of Botany, Chinese Academy of Sciences (KUN)	**OQ685947**
Stenocoelium popovii	Houxia, Wulumuqi, Xinjiang, China	LLJ22080501	Herbarium, College of Life Sciences, Sichuan University (SZ)	**OR529379**

Table S2 The newly sequenced nrDNA in the present study with taxa, source, voucher and GenBank accession numbers.

Taxa	Locality	Voucher information	Herbarium information	GenBank Accession	
				ITS	ETS
Libanotis abolinii	Bayannaoer, Neimenggu, China	LLJ23082001	Herbarium, College of Life Sciences, Sichuan University (SZ)	**OR884086**	**OR901305**
Libanotis acaulis	Bayinbuluke, Hejing, Xinjiang, China	LLJ21070702_2	Herbarium, College of Life Sciences, Sichuan University (SZ)	**OR905805**	**OR901306**
Libanotis buchtormensis	Aletai, Xinjiang, China	LLJ21071112_2	Herbarium, College of Life Sciences, Sichuan University (SZ)	**OR905808**	**OR901307**
Libanotis condensata	Kanasi, Xinjiang, China	CJ21070702_1	Herbarium, College of Life Sciences, Sichuan University (SZ)	**OR905811**	**OR901308**
Libanotis depressa	Nangqian, Qinghai, China	LLJ23080901	Herbarium, College of Life Sciences, Sichuan University (SZ)	**OR905814**	**OR901309**
Libanotis grubovii HJ	Baluntai, Hejing, Xinjiang, China	LLJ23072701	Herbarium, College of Life Sciences, Sichuan University (SZ)	**OR905817**	**OR901311**
Libanotis grubovii WLMQ	Mt. Bogeda, Wulumuqi, Xinjiang, China	LLJ23072401	Herbarium, College of Life Sciences, Sichuan University (SZ)	**OR905816**	**OR901310**
Libanotis iliensis WLMQ	Wulumuqi, Xinjiang, China	LLJ23072201	Herbarium, College of Life Sciences, Sichuan University (SZ)	**OR905819**	**OR901312**
Libanotis iliensis XY	Xinyuan, Xinjiang, China	LLJ23072901	Herbarium, College of Life Sciences, Sichuan University (SZ)	**OR905820**	**OR901313**
Libanotis incana	Tuoli, Xinjiang, China	CJ22072302-01	Herbarium, College of Life Sciences, Sichuan University (SZ)	**OR905821**	**OR901314**
Libanotis jinanensis	Mt. Hu, Jinan, Shandong, China	LLJ22100101-1	Herbarium, College of Life Sciences, Sichuan University (SZ)	**OR905823**	**OR901315**
Libanotis lancifolia	Linfen, Shanxi, China	LLJ23082201	Herbarium, College of Life Sciences, Sichuan University (SZ)	**OR905825**	**OR901316**
Libanotis lanzhouensis	Mt. Lan, Lanzhou, Gansu, China	LLJ23080801	Herbarium, College of Life Sciences, Sichuan University (SZ)	**OR905826**	**OR901317**
Libanotis laticalycina LF	Linfen, Shanxi, China	LLJ23082202	Herbarium, College of Life Sciences, Sichuan University (SZ)	**OR905828**	**OR901318**
Libanotis laticalycina RC	Shuangmiao, RuiCheng, Shanxi, China	LLJ23082401	Herbarium, College of Life Sciences, Sichuan University (SZ)	**OR905829**	**OR901319**

Libanotis schrenkiana TL	Tuoli, Xinjiang, China	CJ220723-4	Herbarium, College of Life Sciences, Sichuan University (SZ)	**OR905830**	**OR901320**
Libanotis schrenkiana TKS	Tekesi, Xinjiang, China	CJ220721-3-1	Herbarium, College of Life Sciences, Sichuan University (SZ)	**OR905831**	**OR901322**
Libanotis seseloides	Eerguna, Neimenggu, China	LLJ23081601	Herbarium, College of Life Sciences, Sichuan University (SZ)	**OR905832**	**OR901321**
Libanotis sibirica	Kanasi, Xinjiang, China	LLJ21071317_2	Herbarium, College of Life Sciences, Sichuan University (SZ)	**OR905834**	**OR901323**
Libanotis spodotrichoma	Huyi, Xian, Shaanxi, China	LLJ23082501	Herbarium, College of Life Sciences, Sichuan University (SZ)	**OR905836**	**OR901324**
Libanotis wannienchun	Chengxian, Longnan, Gansu, China	LLJ23082701	Herbarium, College of Life Sciences, Sichuan University (SZ)	**OR905837**	**OR901325**
Saposhnikovia divaricata 1	Lindong, Chifeng, Neimenggu, China	0709FF	Herbarium, College of Life Sciences, Sichuan University (SZ)	**OR905838**	**OR901326**
Saposhnikovia divaricata 2	Lindong, Chifeng, Neimenggu, China	WZZ	Herbarium, College of Life Sciences, Sichuan University (SZ)	**OR905839**	**OR901327**
Stenocoelium popovii	Houxia, Wulumuqi, Xinjiang, China	LLJ22080501	Herbarium, College of Life Sciences, Sichuan University (SZ)	**OR905841**	**OR901328**

Table S3 List of unique genes identified in plastomes of twelve *Libanotis* newly sequenced.

**Category of Genes**	**Group of gene**	**Name of gene**
Self-replication	Ribosomal RNA genes	*rrn*4.5*, rrn*5*, rrn*16*, rrn*23
	Transfer RNA genes	*trn*C-GCA, *trn*D-GUC, *trn*E-UUC, *trn*F-GAA, *trn*G-GCC, *trn*G-UCC*, *trn*H-GUG, *trn*I-CAU, *trn*K-UUU*, *trn*L-CAA, *trn*L-UAA*, *trn*L-UAG, *trn*M-CAU, *trn*P-UGG, *trn*Q-UUG, *trn*R-UCU, *trn*S-GCU, *trn*S-GGA, *trn*S-UGA, *trn*T-UGU, *trn*T-GGU, *trn*V-GAC, *trn*V-UAC*, *trn*Y-GUA, *trn*W-CCA, *trnf*M-CAU, *trn*A-UGC*, *trn*I-GAU*, *trn*N-GUU, *trn*R-ACG
	Ribosomal protein (small subunit)	*rps*2, *rps*3, *rps*4, *rps*7, *rps*8, *rps*11, *rps*12**, *rps*14, *rps*15, *rps*16*, *rps*18, *rps*19
	Ribosomal protein (large subunit)	*rpl*2*, *rpl*14, *rpl*16*, *rpl*20, *rpl*22, *rpl*23, *rpl*32, *rpl*33, *rpl*36
	RNA polymerase	*rpo*A, *rpo*B, *rpo*C1*, *rpo*C2
	Translational initiation factor	*inf*A
Genes for photosynthesis	Subunits of photosystem I	*psa*A, *psa*B, *psa*C, *psa*I， *psa*J, *ycf*3**, *ycf*4
	Subunits of photosystem II	*psb*A, *psb*B, *psb*C, *psb*D, *psb*E, *psb*F, *psb*H, *psb*I, *psb*J, *psb*K, *psb*L, *psb*M, *psb*N, *psb*T, *psb*Z
	Subunits of cytochrome	*pet*A, *pet*B*, *pet*D*, *pet*G, *pet*L, *pet*N
	Subunits of ATP synthase	*atp*A, *atp*B, *atp*E, *atp*F*, *atp*H, *atp*I
	Large subunit of Rubisco	*rbc*L
	Subunits of NADH dehydrogenase	*ndh*A*, *ndh*B*, *ndh*C, *ndh*D, *ndh*E, *ndh*F, *ndh*G, *ndh*H, *ndh*I, *ndh*J, *ndh*K
Other genes	Maturase	*mat*K
	Envelope membrane protein	*cem*A
	Subunit of acetyl-CoA	*acc*D
	Synthesis gene	*ccs*A
	ATP-dependent protease	*clp*P**
	Component of TIC complex	*ycf*1#
Genes of unknown function	Conserved open reading frames	*ycf*2, *ycf*15

*: Gene with one intron

**: Gene with two introns.

#: This gene exists one pseudogene copy.

Table S4 Plastomes included in phylogenetic analyses with GenBank accession and length. Bolded are newly sequenced sequences. (*) to denote the sequences from us.

Taxa	GenBank Accession	Length (bp)
Angelica dahurica	MT921980*	147477
Angelica decursiva	KT781591	146719
Angelica gigas	KX118044	146918
Angelica sylvestris	NC051890*	147158
Cnidium monnieri	OL839918*	147371
Heracleum moellendorffii	MK210561*	149349
Heracleum yungningense	MN893285*	149223
Kitagawia baicalensis	NC070403*	147985
Kitagawia formosana	NC070399*	146999
Kitagawia praeruptora	MN016968	147197
Kitagawia stepposa	NC070402*	147965
Kitagawia terebinthacea	NC070400*	148327
Ledebouriella seseloides	KT153021	147880
Libanotis acaulis	**OR529367***	147829
Libanotis buchtormensis	MZ707534	147036
Libanotis buchtormensis ZS	**OR529368***	148048
Libanotis condensata	**OR529369***	147763
Libanotis depressa	**OR529370***	148100
Libanotis grubovii	**OR529371***	147471
Libanotis iliensis	**OR529372***	147795
Libanotis incana	**PP078851***	147273
Libanotis jinanensis	**OR529374***	147488
Libanotis lanzhouensis	**OR529375***	147742
Libanotis schrenkiana	**OR529376***	146960
Libanotis seseloides	**OR529377***	147950
Libanotis sibirica	**OR529378***	146836
Libanotis spodotrichoma	MZ707535	147348
Ligusticopsis hispida	MT409614*	147797
Ligusticopsis rechingeriana	NC065482*	148525
Ligusticum involucratum	MT409615*	147752
Ligusticum likiangense	MT409616*	148196
Ligusticum mucronatum	ON975067*	147384
Ligusticum scapiforme	MT409618*	148107
Ligusticum thomsonii	MT409619*	147462
Melanosciadium pimpinelloideum	MN810920*	164431
Pachypleurum alpinum	**OQ685947***	147655
Peucedanum ampliatum	OK336475*	147403
Peucedanum chujaense	MT233391	147839
Peucedanum harry-smithii var. *grande*	OK336476*	147046
Peucedanum japonicum	OK336477*	147592
Peucedanum longshengense	OK336479*	147967
Peucedanum mashanense	OK336478*	154230
Peucedanum medicum	OK336473*	152288
Saposhnikovia divaricata	NC050292	147834

Semenovia transiliensis	NC045182*	142143
Seseli coronatum	ON975066*	145937
Seseli eriocephalum	ON975064*	155617
Seseli glabratum	ON975063*	149039
Seseli incisodentatum	ON975062*	154590
Seseli intramongolicum	ON975061*	151526
Seseli mairei	ON975059*	145859
Seseli mairei var. *simplicifolia*	ON975060*	144957
Seseli montanum	KM035851	147823
Seseli squarrulosum	ON975058*	154502
Seseli valentinae	ON975057*	147460
Seseli yunnanense	ON975056*	145975
Stenocoelium popovii	**OR529379***	147519

Table S5 nrDNA (ITS and ETS) included in phylogenetic analyses with GenBank accession. Bolded are newly sequenced sequences. (*) to denote the sequences from us.

Taxa	GenBank Accession	
	ITS	ETS
Angelica dahurica	EU418374*	HM443672*
Angelica decursiva	AY548220	HM443674*
Angelica gigas	AJ131290	HM443678*
Angelica laxifoliata	DQ263586	JN107557*
Angelica nitida	DQ263592	FJ986017*
Heracleum moellendorffii	FJ986050*	FJ986032*
Heracleum yungningense	FJ812116*	FJ986039*
Kitagawia praeruptora	ON974239*	ON983960*
Libanotis abolinii	**OR884086***	**OR901305***
Libanotis acaulis	**OR905805***	**OR901306***
Libanotis buchtormensis	**OR905808***	**OR901307***
Libanotis condensata	**OR905811***	**OR901308***
Libanotis depressa	**OR905814***	**OR901309***
Libanotis grubovii HJ	**OR905817***	**OR901311***
Libanotis grubovii WLMQ	**OR905816***	**OR901310***
Libanotis iliensis WLMQ	**OR905819***	**OR901312***
Libanotis iliensis XY	**OR905820***	**OR901313***
Libanotis incana	**OR905821***	**OR901314***
Libanotis jinanensis	**OR905823***	**OR901315***
Libanotis lancifolia	**OR905825***	**OR901316***
Libanotis lanzhouensis	**OR905826***	**OR901317***
Libanotis laticalycina LF	**OR905828***	**OR901318***
Libanotis laticalycina RC	**OR905829***	**OR901319***
Libanotis schrenkiana	**OR905830***	**OR901320***
Libanotis seseloides	**OR905832***	**OR901321***
Libanotis sibirica KNS	**OR905834***	**OR901323***
Libanotis schrenkiana TL	**OR905831***	**OR901322***
Libanotis spodotrichoma	**OR905836***	**OR901324***
Libanotis wannienchun	**OR905837***	**OR901325***
Ligusticopsis angelicifolia	GU395174*	HM443702*
Ligusticum mucronatum	ON980812*	ON980799*
Peucedanum ampliatum	ON974235*	ON983956*
Peucedanum harry-smithii var. *grande*	ON974236*	ON983957*
Peucedanum mashanense	ON974237*	ON983958*
Peucedanum medicum	ON974238*	ON983959*
Peucedanum morisonii	AF077903	JF807531
Peucedanum officinale	KP682404	JF807530
Saposhnikovia divaricata	ON974234*	ON983955*
Saposhnikovia divaricata 1	**OR905838***	**OR901326***
Saposhnikovia divaricata 2	**OR905839***	**OR901327***
Semenovia transiliensis	MF803821*	MF803788*
Seseli alexeenkoi	MG697118	MG697160
Seseli andronakii	MG697135	MG697177
Seseli arenarium	MG697123	MG697166
Seseli coronatum	ON980808*	ON980795*

Seseli corymbosum	MG697129	MG697171
Seseli dichotomum	MG697116	MG697158
Seseli eriocephalum	ON980809*	ON980796*
Seseli glabratum	ON980805*	ON980792*
Seseli globiferum	MG697147	MG697179
Seseli grandivittatum	MG697120	MG697162
Seseli gummiferum	MG697127	MG697169
Seseli hartvigii	MG697138	MG697180
Seseli incisodentatum	ON980804*	ON980791*
Seseli intramongolicum	ON980806*	ON980793*
Seseli lehmannii	MG697114	MG697156
Seseli leptocladum	MG697117	MG697159
Seseli libanotis	MG697128	MG697170
Seseli mairei	ON980801*	ON980788*
Seseli mairei var. *simplicifolia*	ON980802*	ON980789*
Seseli marashicum	MG697143	MG697185
Seseli paphlagonicum	MG697130	MG697172
Seseli ponticum	MG697115	MG697161
Seseli resinosum	MG697132	MG697174
Seseli rupicola	MG697126	MG697168
Seseli serpentinum	MG697142	MG697184
Seseli squarrulosum	ON980803*	ON980790*
Seseli tortuosum	MG697151	MG697190
Seseli transcaucasicum	MG697121	MG697163
Seseli valentinae	ON980807*	ON980794*
Seseli yunnanense	ON980800*	ON980787*
Stenocoelium popovii	**OR905841***	**OR901328***

Table S6 Simple sequence repeats (SSRs) distribution in the thirteen *Libanotis* plastomes. These data were visualized in Figure S1.

Taxon	SSR nr.	SSR type	Motif	size (bp)	Start	End	Region
L.acaulis	1	p4	(TTTA)3	12	106	117	LSC
	2	p4	(TTTA)3	12	121	132	LSC
	3	p1	(A)11	11	139	149	LSC
	4	p1	(A)10	10	1518	1527	LSC
	5	p4	(AATA)3	12	3855	3866	LSC
	6	p1	(A)13	13	4194	4206	LSC
	7	p2	(TA)7	14	4780	4793	LSC
	8	p2	(AT)5	10	4811	4820	LSC
	9	p1	(A)13	13	8642	8654	LSC
	10	p1	(T)11	11	8914	8924	LSC
	11	p1	(T)10	10	9379	9388	LSC
	12	p2	(AT)5	10	9854	9863	LSC
	13	p2	(AT)5	10	10042	10051	LSC
	14	p2	(AT)8	16	13060	13075	LSC
	15	p4	(TTTA)3	12	13137	13148	LSC
	16	p1	(A)10	10	13543	13552	LSC
	17	p1	(A)11	11	16385	16395	LSC
	18	p1	(T)11	11	18615	18625	LSC
	19	p2	(AT)5	10	19985	19994	LSC
	20	p1	(T)10	10	22794	22803	LSC
	21	p4	(AAAT)3	12	22814	22825	LSC
	22	p1	(T)12	12	26354	26365	LSC
	23	p1	(A)10	10	27371	27380	LSC
	24	p1	(T)11	11	28005	28015	LSC
	25	p1	(T)10	10	29523	29532	LSC
	26	p2	(AT)6	12	30008	30019	LSC
	27	p1	(T)10	10	31959	31968	LSC
	28	p3	(TTA)4	12	32017	32028	LSC
	29	p1	(T)11	11	32666	32676	LSC
	30	p1	(A)10	10	33042	33051	LSC
	31	p1	(A)10	10	33216	33225	LSC
	32	p1	(C)11	11	36998	37008	LSC
	33	p1	(A)15	15	37076	37090	LSC
	34	p5	(TTTTA)3	15	37737	37751	LSC
	35	p4	(ATTT)3	12	42803	42814	LSC
	36	p1	(A)12	12	43358	43369	LSC
	37	p1	(T)10	10	45171	45180	LSC
	38	p1	(A)16	16	45552	45567	LSC
	39	p2	(TA)6	12	47984	47995	LSC
	40	p2	(AT)5	10	50201	50210	LSC
	41	p1	(T)11	11	52572	52582	LSC
	42	p1	(T)10	10	52750	52759	LSC
	43	p1	(T)10	10	55530	55539	LSC
	44	p3	(ATA)4	12	55584	55595	LSC
	45	p1	(A)12	12	56114	56125	LSC

	46	p1	(T)11	11	60398	60408	LSC
	47	p1	(T)15	15	60642	60656	LSC
	48	p1	(A)12	12	63972	63983	LSC
	49	p2	(TA)5	10	64015	64024	LSC
	50	p4	(TCCT)3	12	67924	67935	LSC
	51	p1	(A)10	10	68800	68809	LSC
	52	p1	(T)10	10	69950	69959	LSC
	53	p1	(A)11	11	70628	70638	LSC
	54	p1	(A)10	10	71733	71742	LSC
	55	p1	(T)12	12	72481	72492	LSC
	56	p1	(T)10	10	78599	78608	LSC
	57	p1	(T)12	12	82995	83006	LSC
	58	p2	(AT)5	10	84374	84383	LSC
	59	p1	(T)13	13	84694	84706	LSC
	60	p1	(G)12	12	94277	94288	IRb
	61	p1	(T)19	19	99333	99351	IRb
	62	p1	(T)10	10	103345	103354	IRb
	63	p1	(G)13	13	104566	104578	IRb
	64	p4	(AGGT)3	12	106656	106667	IRb
	65	p1	(T)10	10	108829	108838	IRb
	66	p1	(A)10	10	111171	111180	IRb
	67	p2	(TA)8	16	114842	114857	SSC
	68	p2	(AT)6	12	115472	115483	SSC
	69	p2	(AT)6	12	115486	115497	SSC
	70	p2	(AT)6	12	115506	115517	SSC
	71	p4	(TCTT)3	12	116538	116549	SSC
	72	p1	(A)14	14	116584	116597	SSC
	73	p1	(T)10	10	117467	117476	SSC
	74	p1	(A)11	11	120104	120114	SSC
	75	p1	(T)10	10	120899	120908	SSC
	76	p4	(CAAT)3	12	120972	120983	SSC
	77	p1	(A)10	10	122555	122564	SSC
	78	p2	(TA)5	10	127289	127298	SSC
	79	p1	(T)13	13	127828	127840	SSC
	80	p1	(T)10	10	128226	128235	SSC
	81	p1	(T)10	10	128495	128504	SSC
	82	p1	(T)11	11	129012	129022	SSC
	83	p1	(T)10	10	129806	129815	IRa
	84	p1	(A)10	10	132148	132157	IRa
	85	p4	(CTAC)3	12	134317	134328	IRa
	86	p1	(C)13	13	136408	136420	IRa
	87	p1	(A)10	10	137632	137641	IRa
	88	p1	(A)19	19	141635	141653	IRa
	89	p1	(C)12	12	146698	146709	IRa
L. buchtormensis	1	p4	(TTTA)3	12	150	161	LSC
	2	p4	(TTAT)3	12	176	187	LSC
	3	p1	(A)11	11	193	203	LSC
	4	p1	(A)10	10	1572	1581	LSC

5	p4	(AATA)3	12	3924	3935	LSC
6	p1	(A)14	14	4264	4277	LSC
7	p2	(TA)5	10	4845	4854	LSC
8	p3	(TTA)5	15	5453	5467	LSC
9	p1	(G)11	11	8807	8817	LSC
10	p1	(T)10	10	8943	8952	LSC
11	p1	(A)10	10	9425	9434	LSC
12	p2	(AT)5	10	9884	9893	LSC
13	p2	(AT)5	10	10072	10081	LSC
14	p2	(AT)6	12	13083	13094	LSC
15	p1	(A)15	15	16388	16402	LSC
16	p1	(T)11	11	18611	18621	LSC
17	p2	(AT)5	10	19975	19984	LSC
18	p4	(AAAT)3	12	22800	22811	LSC
19	p1	(T)12	12	26340	26351	LSC
20	p1	(A)10	10	27357	27366	LSC
21	p3	(AAT)4	12	28574	28585	LSC
22	p1	(T)11	11	29525	29535	LSC
23	p2	(AT)5	10	30016	30025	LSC
24	p1	(T)10	10	31959	31968	LSC
25	p1	(T)14	14	32653	32666	LSC
26	p1	(A)10	10	33031	33040	LSC
27	p3	(TTA)4	12	36637	36648	LSC
28	p1	(A)10	10	37424	37433	LSC
29	p4	(ATTT)3	12	42769	42780	LSC
30	p1	(A)10	10	43289	43298	LSC
31	p1	(A)19	19	43321	43339	LSC
32	p1	(A)17	17	45522	45538	LSC
33	p1	(T)10	10	49030	49039	LSC
34	p2	(AT)5	10	50150	50159	LSC
35	p2	(TA)5	10	51794	51803	LSC
36	p1	(T)12	12	52536	52547	LSC
37	p1	(T)10	10	52719	52728	LSC
38	p1	(T)10	10	55499	55508	LSC
39	p3	(ATA)4	12	55553	55564	LSC
40	p1	(A)14	14	56084	56097	LSC
41	p1	(T)10	10	57867	57876	LSC
42	p1	(T)12	12	60390	60401	LSC
43	p2	(TA)5	10	64015	64024	LSC
44	p5	(TATAT)3	15	66901	66915	LSC
45	p2	(TA)5	10	66911	66920	LSC
46	p4	(TCCT)3	12	67969	67980	LSC
47	p1	(A)10	10	71793	71802	LSC
48	p1	(T)11	11	72547	72557	LSC
49	p2	(AT)5	10	77657	77666	LSC
50	p1	(A)11	11	81676	81686	LSC
51	p1	(T)10	10	81761	81770	LSC
52	p1	(T)12	12	83068	83079	LSC
53	p2	(AT)5	10	84447	84456	LSC

	54	p1	(T)18	18	84767	84784	LSC
	55	p1	(T)11	11	99320	99330	IRb
	56	p1	(T)10	10	103304	103313	IRb
	57	p1	(G)11	11	104545	104555	IRb
	58	p4	(AGGT)3	12	106633	106644	IRb
	59	p1	(A)10	10	111153	111162	IRb
	60	p1	(A)11	11	111937	111947	IRb
	61	p2	(TA)7	14	115696	115709	SSC
	62	p2	(AT)5	10	115724	115733	SSC
	63	p4	(TCTT)3	12	116742	116753	SSC
	64	p1	(A)13	13	116789	116801	SSC
	65	p1	(A)11	11	120349	120359	SSC
	66	p2	(TA)5	10	120957	120966	SSC
	67	p1	(T)11	11	121167	121177	SSC
	68	p4	(CAAT)3	12	121241	121252	SSC
	69	p2	(TA)5	10	127560	127569	SSC
	70	p1	(T)15	15	128087	128101	SSC
	71	p1	(T)10	10	128712	128721	SSC
	72	p1	(T)11	11	129229	129239	IRa
	73	p1	(T)10	10	130014	130023	IRa
	74	p4	(CTAC)3	12	134530	134541	IRa
	75	p1	(C)11	11	136621	136631	IRa
	76	p1	(A)10	10	137863	137872	IRa
	77	p1	(A)11	11	141846	141856	IRa
L. condensata	1	p1	(T)17	17	315	331	LSC
	2	p4	(TTTA)3	12	960	971	LSC
	3	p1	(A)10	10	2609	2618	LSC
	4	p4	(AATA)3	12	4684	4695	LSC
	5	p1	(A)11	11	5024	5034	LSC
	6	p2	(TA)6	12	5614	5625	LSC
	7	p2	(TA)6	12	5638	5649	LSC
	8	p1	(T)11	11	8901	8911	LSC
	9	p1	(G)14	14	9572	9585	LSC
	10	p1	(T)11	11	9696	9706	LSC
	11	p2	(TA)8	16	10650	10665	LSC
	12	p2	(AT)5	10	10832	10841	LSC
	13	p2	(AT)7	14	13850	13863	LSC
	14	p4	(TTTA)4	16	13925	13940	LSC
	15	p1	(A)11	11	14333	14343	LSC
	16	p1	(T)11	11	19386	19396	LSC
	17	p2	(AT)5	10	20750	20759	LSC
	18	p4	(AAAT)3	12	23575	23586	LSC
	19	p1	(T)12	12	27115	27126	LSC
	20	p1	(T)10	10	28726	28735	LSC
	21	p1	(T)11	11	28954	28964	LSC
	22	p3	(TTA)4	12	29347	29358	LSC
	23	p1	(T)11	11	30335	30345	LSC
	24	p2	(AT)5	10	30818	30827	LSC

25	p1	(T)11	11	32795	32805	LSC
26	p1	(A)14	14	34045	34058	LSC
27	p1	(A)10	10	38288	38297	LSC
28	p5	(TTTTA)3	15	38578	38592	LSC
29	p4	(ATTT)3	12	43625	43636	LSC
30	p1	(A)15	15	44191	44205	LSC
31	p1	(T)10	10	46007	46016	LSC
32	p1	(A)10	10	46389	46398	LSC
33	p2	(TA)9	18	46628	46645	LSC
34	p2	(AT)5	10	51096	51105	LSC
35	p2	(TA)5	10	52744	52753	LSC
36	p1	(T)10	10	56470	56479	LSC
37	p3	(ATA)4	12	56524	56535	LSC
38	p1	(T)10	10	56558	56567	LSC
39	p1	(A)11	11	57065	57075	LSC
40	p1	(T)10	10	58845	58854	LSC
41	p1	(T)11	11	61403	61413	LSC
42	p2	(AT)6	12	65008	65019	LSC
43	p2	(AT)6	12	65066	65077	LSC
44	p2	(TA)5	10	67952	67961	LSC
45	p4	(TCCT)3	12	69006	69017	LSC
46	p1	(A)13	13	69889	69901	LSC
47	p1	(T)10	10	72015	72024	LSC
48	p1	(T)10	10	73585	73594	LSC
49	p2	(AT)5	10	78654	78663	LSC
50	p1	(A)10	10	82691	82700	LSC
51	p1	(T)10	10	83719	83728	LSC
52	p1	(T)10	10	84082	84091	LSC
53	p2	(AT)5	10	85459	85468	LSC
54	p1	(T)17	17	85779	85795	LSC
55	p1	(G)15	15	94432	94446	IRb
56	p1	(T)14	14	99477	99490	IRb
57	p1	(T)14	14	103470	103483	IRb
58	p1	(G)10	10	104715	104724	IRb
59	p4	(AGGT)3	12	106811	106822	IRb
60	p1	(A)10	10	111333	111342	IRb
61	p2	(TA)10	20	115009	115028	SSC
62	p2	(TA)5	10	115643	115652	SSC
63	p2	(AT)5	10	115686	115695	SSC
64	p2	(AT)5	10	115832	115841	SSC
65	p4	(TCTT)3	12	116710	116721	SSC
66	p1	(A)13	13	116757	116769	SSC
67	p1	(A)12	12	120282	120293	SSC
68	p1	(T)11	11	121078	121088	SSC
69	p4	(CAAT)3	12	121151	121162	SSC
70	p2	(TA)5	10	127440	127449	SSC
71	p1	(T)15	15	127967	127981	SSC
72	p1	(T)10	10	128652	128661	SSC
73	p1	(T)11	11	129169	129179	SSC

	74	p1	(T)10	10	129954	129963	IRa
	75	p4	(CTAC)3	12	134472	134483	IRa
	76	p1	(C)10	10	136572	136581	IRa
	77	p1	(A)14	14	137813	137826	IRa
	78	p1	(A)14	14	141806	141819	IRa
	79	p1	(C)15	15	146850	146864	IRa
L. depressa	1	p1	(T)10	10	89	98	LSC
	2	p4	(TTTA)3	12	96	107	LSC
	3	p1	(A)10	10	124	133	LSC
	4	p1	(A)12	12	1502	1513	LSC
	5	p1	(A)11	11	1759	1769	LSC
	6	p4	(AATA)3	12	3843	3854	LSC
	7	p2	(TA)5	10	4755	4764	LSC
	8	p1	(T)11	11	7334	7344	LSC
	9	p1	(G)10	10	7996	8005	LSC
	10	p1	(T)11	11	8131	8141	LSC
	11	p2	(AT)5	10	9243	9252	LSC
	12	p1	(A)10	10	10885	10894	LSC
	13	p1	(A)11	11	12195	12205	LSC
	14	p2	(AT)8	16	12258	12273	LSC
	15	p1	(T)10	10	13714	13723	LSC
	16	p1	(A)17	17	15607	15623	LSC
	17	p1	(T)11	11	17832	17842	LSC
	18	p2	(AT)5	10	19196	19205	LSC
	19	p1	(T)11	11	22011	22021	LSC
	20	p4	(AAAT)3	12	22030	22041	LSC
	21	p1	(T)12	12	25572	25583	LSC
	22	p6	(AGATAT)3	18	26807	26824	LSC
	23	p1	(T)10	10	28806	28815	LSC
	24	p2	(AT)5	10	29302	29311	LSC
	25	p2	(AT)5	10	30983	30992	LSC
	26	p1	(A)12	12	32484	32495	LSC
	27	p1	(A)11	11	36734	36744	LSC
	28	p5	(TTTTA)3	15	37024	37038	LSC
	29	p4	(ATTT)3	12	42071	42082	LSC
	30	p1	(A)13	13	42628	42640	LSC
	31	p1	(A)16	16	44828	44843	LSC
	32	p4	(AAAT)3	12	44845	44856	LSC
	33	p2	(AT)5	10	49469	49478	LSC
	34	p1	(T)11	11	50414	50424	LSC
	35	p2	(TA)5	10	51121	51130	LSC
	36	p1	(T)13	13	52873	52885	LSC
	37	p5	(AATCA)3	15	52935	52949	LSC
	38	p1	(T)10	10	54839	54848	LSC
	39	p3	(ATA)4	12	54893	54904	LSC
	40	p1	(T)10	10	54927	54936	LSC
	41	p1	(T)10	10	57202	57211	LSC
	42	p1	(T)10	10	59753	59762	LSC

	43	p1	(T)10	10	59982	59991	LSC
	44	p2	(TA)6	12	63357	63368	LSC
	45	p2	(TA)6	12	66240	66251	LSC
	46	p4	(TCCT)3	12	67291	67302	LSC
	47	p1	(T)13	13	70302	70314	LSC
	48	p1	(T)10	10	71864	71873	LSC
	49	p2	(AT)5	10	76921	76930	LSC
	50	p1	(T)10	10	82342	82351	LSC
	51	p2	(AT)5	10	83719	83728	LSC
	52	p1	(T)11	11	84039	84049	LSC
	53	p1	(A)10	10	93027	93036	IRb
	54	p1	(T)15	15	98671	98685	IRb
	55	p1	(G)11	11	103876	103886	IRb
	56	p4	(AGGT)3	12	105991	106002	IRb
	57	p1	(A)10	10	110569	110578	IRb
	58	p1	(A)10	10	111067	111076	IRb
	59	p2	(TA)5	10	111088	111097	SSC
	60	p2	(TA)6	12	114237	114248	SSC
	61	p2	(AT)6	12	114881	114892	SSC
	62	p4	(TCTT)3	12	115911	115932	SSC
	63	p1	(A)14	14	115957	115970	SSC
	64	p1	(A)10	10	119472	119481	SSC
	65	p2	(TA)5	10	120079	120088	SSC
	66	p4	(CAAT)3	12	120342	120353	SSC
	67	p2	(TA)5	10	126660	126669	SSC
	68	p1	(T)10	10	127851	127860	SSC
	69	p1	(T)10	10	129153	129162	IRa
	70	p4	(CTAC)3	12	133727	133738	IRa
	71	p1	(C)11	11	135845	135855	IRa
	72	p1	(A)15	15	141046	141060	IRa
	73	p1	(T)10	10	146689	146698	IRa
L. grubovii	1	p4	(TTTA)3	12	103	114	LSC
	2	p1	(A)10	10	1757	1766	LSC
	3	p4	(AATA)3	12	3853	3864	LSC
	4	p1	(A)11	11	4192	4202	LSC
	5	p5	(ACTTT)3	15	6191	6205	LSC
	6	p1	(T)10	10	8089	8098	LSC
	7	p3	(TTA)5	15	8804	8818	LSC
	8	p2	(AT)5	10	9845	9854	LSC
	9	p2	(TA)6	12	9859	9870	LSC
	10	p2	(AT)5	10	10037	10046	LSC
	11	p1	(T)10	10	12374	12383	LSC
	12	P1	(A)10	10	12991	13000	LSC
	13	P2	(AT)6	12	13049	13060	LSC
	14	p4	(TTTA)3	12	13133	13144	LSC
	15	p1	(A)17	17	16340	16356	LSC
	16	p1	(T)11	11	18565	18575	LSC
	17	p2	(AT)5	10	19935	19944	LSC

18	p4	(AAAT)3	12	22761	22772	LSC
19	p1	(T)12	12	26284	26295	LSC
20	p1	(A)11	11	27301	27311	LSC
21	p1	(T)11	11	29531	29541	LSC
22	p2	(AT)6	12	30037	30048	LSC
23	p1	(T)10	10	30862	30871	LSC
24	p2	(AT)5	10	31699	31708	LSC
25	p1	(T)16	16	32664	32679	LSC
26	p1	(A)11	11	33238	33248	LSC
27	p1	(A)10	10	37107	37116	LSC
28	p1	(A)12	12	37473	37484	LSC
29	p5	(TTTTA)3	15	37765	37779	LSC
30	p4	(ATTT)3	12	42812	42823	LSC
31	p1	(A)14	14	43370	43383	LSC
32	p5	(TATAT)3	15	43900	43914	LSC
33	p1	(T)12	12	45190	45201	LSC
34	p4	(AAAT)3	12	45576	45587	LSC
35	p2	(AT)5	10	45803	45812	LSC
36	p2	(AT)5	10	50166	50175	LSC
37	p1	(T)10	10	52729	52738	LSC
38	p1	(T)10	10	55509	55518	LSC
39	p1	(A)13	13	56099	56111	LSC
40	p1	(T)10	10	57881	57890	LSC
41	p4	(AAAT)3	12	58304	58315	LSC
42	p1	(T)11	11	60413	60423	LSC
43	p1	(T)14	14	60657	60670	LSC
44	P1	(A)11	11	64006	64016	LSC
45	P2	(TA)5	10	64040	64049	LSC
46	P2	(AT)5	10	64072	64081	LSC
47	p4	(TCCT)3	12	67950	67961	LSC
48	p1	(A)10	10	68832	68841	LSC
49	p1	(A)11	11	70679	70689	LSC
50	p1	(T)10	10	70965	70974	LSC
51	p1	(A)10	10	71705	71714	LSC
52	p1	(T)10	10	72522	72531	LSC
53	p2	(AT)5	10	77598	77607	LSC
54	p1	(T)10	10	81052	81061	LSC
55	p1	(A)13	13	81619	81631	LSC
56	p1	(T)12	12	83011	83022	LSC
57	p2	(AT)5	10	84390	84399	LSC
58	p1	(T)13	13	84710	84722	LSC
59	p1	(A)10	10	93309	93318	IRb
60	p1	(G)12	12	94252	94263	IRb
61	p1	(T)14	14	99299	99312	IRb
62	p1	(G)12	12	104514	104525	IRb
63	p4	(AGGT)3	12	106603	106614	IRb
64	p1	(A)10	10	111117	111126	IRb
65	p2	(TA)7	14	114788	114801	IRb
66	p2	(TA)6	12	115282	115293	IRb

	67	p2	(AT)5	10	115295	115304	IRb
	68	p2	(AT)5	10	115320	115329	IRb
	69	p4	(TCTT)3	12	116331	116342	IRb
	70	p1	(A)13	13	116378	116390	IRb
	71	p4	(TTGA)3	12	119456	119467	SSC
	72	p1	(A)14	14	119894	119907	SSC
	73	p4	(CAAT)3	12	120764	120775	SSC
	74	p5	(ATAGT)3	15	122867	122881	SSC
	75	p2	(TA)5	10	127083	127092	SSC
	76	p1	(T)13	13	127610	127622	SSC
	77	p1	(T)10	10	128277	128286	SSC
	78	p1	(T)11	11	128794	128804	SSC
	79	p1	(T)10	10	129579	129588	IRa
	80	p4	(CTAC)3	12	134089	134100	IRa
	81	p1	(C)12	12	136180	136191	IRa
	82	p1	(A)14	14	141393	141406	IRa
	83	p1	(C)12	12	146442	146453	IRa
	84	p1	(T)10	10	147387	147396	IRa
L. iliensis	1	p4	(AATA)3	12	3876	3887	LSC
	2	p1	(A)12	12	4215	4226	LSC
	3	p5	(ACTTT)3	15	6232	6246	LSC
	4	p1	(T)10	10	8130	8139	LSC
	5	p1	(G)12	12	8792	8803	LSC
	6	p3	(TTA)5	15	8849	8863	LSC
	7	p1	(T)11	11	8951	8961	LSC
	8	p2	(AT)5	10	9892	9901	LSC
	9	p2	(AT)5	10	9916	9925	LSC
	10	p2	(TA)6	12	9930	9941	LSC
	11	p2	(AT)5	10	10095	10104	LSC
	12	p1	(T)10	10	12432	12441	LSC
	13	p2	(AT)6	12	13105	13116	LSC
	14	p4	(TTTA)3	12	13189	13200	LSC
	15	p1	(A)14	14	16413	16426	LSC
	16	p1	(T)11	11	18635	18645	LSC
	17	p2	(AT)5	10	20005	20014	LSC
	18	p1	(T)10	10	22816	22825	LSC
	19	p4	(AAAT)3	12	22835	22846	LSC
	20	p1	(T)12	12	26374	26385	LSC
	21	p1	(A)11	11	27391	27401	LSC
	22	p6	(AGATAT)3	18	27612	27629	LSC
	23	p1	(T)10	10	28227	28236	LSC
	24	p1	(T)13	13	29628	29640	LSC
	25	p2	(AT)6	12	30136	30147	LSC
	26	p1	(T)10	10	30967	30976	LSC
	27	p2	(AT)5	10	31797	31806	LSC
	28	p1	(A)10	10	37196	37205	LSC
	29	p5	(TTTTA)3	15	37851	37865	LSC
	30	p4	(ATTT)3	12	42898	42909	LSC

31	p1	(A)10	10	43425	43434	LSC
32	p1	(A)10	10	43457	43466	LSC
33	p1	(T)14	14	45268	45281	LSC
34	p4	(AAAT)3	12	45656	45667	LSC
35	p2	(AT)5	10	45883	45892	LSC
36	p2	(AT)5	10	50285	50294	LSC
37	p1	(T)12	12	52666	52677	LSC
38	p1	(T)10	10	52849	52858	LSC
39	p1	(T)10	10	55629	55638	LSC
40	p1	(A)13	13	56219	56231	LSC
41	p1	(T)10	10	58009	58018	LSC
42	p4	(AAAT)3	12	58432	58443	LSC
43	p1	(T)14	14	60783	60796	LSC
44	p1	(A)11	11	64131	64141	LSC
45	p2	(TA)5	10	64154	64163	LSC
46	p2	(TA)5	10	64165	64174	LSC
47	p5	(TATTA)3	15	64183	64197	LSC
48	p2	(TA)6	12	64199	64210	LSC
49	p4	(GAAA)3	12	65694	65705	LSC
50	p4	(TCCT)3	12	68065	68076	LSC
51	p1	(A)10	10	68940	68949	LSC
52	p1	(A)10	10	70787	70796	LSC
53	p1	(A)12	12	71880	71891	LSC
54	p1	(T)10	10	72630	72639	LSC
55	p2	(AT)5	10	77689	77698	LSC
56	p1	(T)10	10	81144	81153	LSC
57	p1	(A)13	13	81711	81723	LSC
58	p1	(T)12	12	83103	83114	LSC
59	p2	(AT)5	10	84482	84491	LSC
60	p1	(T)13	13	84802	84814	LSC
61	p1	(A)10	10	93401	93410	IRb
62	p1	(G)13	13	94329	94341	IRb
63	p1	(T)15	15	99377	99391	IRb
64	p1	(G)11	11	104603	104613	IRb
65	p4	(AGGT)3	12	106700	106711	IRb
66	p1	(A)10	10	111228	111237	IRb
67	p2	(TA)7	14	114920	114933	SSC
68	p2	(TA)6	12	115407	115418	SSC
69	p2	(AT)5	10	115420	115429	SSC
70	p2	(AT)6	12	115445	115456	SSC
71	p4	(TCTT)3	12	116451	116462	SSC
72	p1	(A)13	13	116498	116510	SSC
73	p4	(TTGA)3	12	119576	119587	SSC
74	p1	(A)13	13	120014	120026	SSC
75	p1	(T)10	10	120811	120820	SSC
76	p4	(CAAT)3	12	120884	120895	SSC
77	p2	(TA)5	10	127188	127197	SSC
78	p1	(T)13	13	127715	127727	SSC
79	p1	(T)10	10	128382	128391	SSC

	80	p1	(T)11	11	128899	128909	SSC
	81	p1	(T)10	10	129684	129693	IRa
	82	p4	(CTAC)3	12	134208	134219	IRa
	83	p1	(C)11	11	136308	136318	IRa
	84	p1	(A)15	15	141530	141544	IRa
	85	p1	(C)13	13	146580	146592	IRa
	86	p1	(T)10	10	147511	147520	IRa
L. incana	1	p4	(TTTA)3	12	172	183	LSC
	2	p1	(A)12	12	201	212	LSC
	3	p1	(A)11	11	1581	1591	LSC
	4	p4	(AATA)3	12	3917	3928	LSC
	5	p1	(A)10	10	4256	4265	LSC
	6	p2	(TA)5	10	4845	4854	LSC
	7	p3	(TTA)4	12	5455	5466	LSC
	8	p1	(T)10	10	7799	7808	LSC
	9	p1	(A)10	10	8326	8335	LSC
	10	p1	(G)10	10	8462	8471	LSC
	11	p1	(T)11	11	8597	8607	LSC
	12	p1	(A)10	10	9080	9089	LSC
	13	p2	(AT)5	10	9519	9528	LSC
	14	p2	(AT)5	10	9545	9554	LSC
	15	p2	(AT)5	10	9740	9749	LSC
	16	p1	(T)10	10	12076	12085	LSC
	17	p2	(AT)7	14	12771	12784	LSC
	18	p2	(AT)7	14	12793	12806	LSC
	19	p4	(TTTA)4	16	12868	12883	LSC
	20	p1	(A)11	11	13278	13288	LSC
	21	p1	(A)12	12	16111	16122	LSC
	22	p1	(T)11	11	18331	18341	LSC
	23	p2	(AT)5	10	19695	19704	LSC
	24	p4	(AAAT)3	12	22521	22532	LSC
	25	p1	(T)12	12	26061	26072	LSC
	26	p1	(A)11	11	27086	27096	LSC
	27	p3	(AAT)4	12	28339	28350	LSC
	28	p1	(T)11	11	29290	29300	LSC
	29	p2	(AT)5	10	29777	29786	LSC
	30	p1	(A)10	10	30652	30661	LSC
	31	p1	(T)10	10	31706	31715	LSC
	32	p1	(A)12	12	32952	32963	LSC
	33	p4	(ATTT)3	12	42524	42535	LSC
	34	p1	(A)10	10	43079	43088	LSC
	35	p1	(T)11	11	44890	44900	LSC
	36	p1	(A)13	13	45280	45292	LSC
	37	p2	(TA)7	14	47106	47119	LSC
	38	p2	(AT)5	10	49977	49986	LSC
	39	p2	(TA)5	10	51625	51634	LSC
	40	p1	(T)12	12	52360	52371	LSC
	41	p1	(T)10	10	55322	55331	LSC

	42	p3	(ATA)4	12	55376	55387	LSC
	43	p1	(A)10	10	55907	55916	LSC
	44	p1	(T)10	10	57686	57695	LSC
	45	p1	(T)11	11	60194	60204	LSC
	46	p1	(A)10	10	63785	63794	LSC
	47	p2	(TA)5	10	63818	63827	LSC
	48	p5	(TATAT)3	15	66678	66692	LSC
	49	p2	(TA)5	10	66702	66711	LSC
	50	p4	(TCCT)3	12	67746	67757	LSC
	51	p1	(A)10	10	71575	71584	LSC
	52	p1	(T)12	12	72323	72334	LSC
	53	p2	(AT)5	10	77403	77412	LSC
	54	p1	(T)10	10	78416	78425	LSC
	55	p1	(A)12	12	81432	81443	LSC
	56	p1	(T)10	10	81518	81527	LSC
	57	p1	(T)11	11	82825	82835	LSC
	58	p2	(AT)5	10	84203	84212	LSC
	59	p1	(T)16	16	84523	84538	LSC
	60	p1	(G)13	13	94078	94090	IRb
	61	p1	(T)13	13	99135	99147	IRb
	62	p1	(T)10	10	103117	103126	IRb
	63	p1	(G)10	10	104358	104367	IRb
	64	p4	(AGGT)3	12	106445	106456	IRb
	65	p1	(T)10	10	108618	108627	IRb
	66	p1	(A)10	10	110960	110969	IRb
	67	p2	(TA)7	14	114879	114892	SSC
	68	p2	(AT)6	12	114909	114920	SSC
	69	p4	(TCTT)3	12	115922	115933	SSC
	70	p1	(A)13	13	115969	115981	SSC
	71	p1	(A)11	11	119518	119528	SSC
	72	p2	(TA)5	10	120126	120135	SSC
	73	p4	(CAAT)3	12	120387	120398	SSC
	74	p2	(TA)5	10	126700	126709	SSC
	75	p1	(T)15	15	127227	127241	SSC
	76	p1	(T)10	10	127852	127861	SSC
	77	p1	(T)11	11	128369	128379	SSC
	78	p1	(T)10	10	129154	129163	IRa
	79	p1	(A)10	10	131496	131505	IRa
	80	p4	(CTAC)3	12	133665	133676	IRa
	81	p1	(C)10	10	135756	135765	IRa
	82	p1	(A)10	10	136997	137006	IRa
	83	p1	(A)13	13	140976	140988	IRa
	84	p1	(C)13	13	146033	146045	IRa
L. jinanensis	1	p4	(TTTA)3	12	106	117	LSC
	2	p1	(A)13	13	1524	1536	LSC
	3	p1	(A)12	12	1782	1793	LSC
	4	p4	(AATA)3	12	3859	3870	LSC
	5	p2	(TA)6	12	4796	4807	LSC

6	p5	(ATTTA)3	15	6515	6529	LSC
7	p1	(T)11	11	8093	8103	LSC
8	p1	(A)11	11	8621	8631	LSC
9	p1	(G)10	10	8758	8767	LSC
10	p1	(T)13	13	8893	8905	LSC
11	p2	(AT)5	10	9843	9852	LSC
12	p2	(AT)5	10	10067	10076	LSC
13	p1	(A)10	10	11710	11719	LSC
14	p1	(A)10	10	13021	13030	LSC
15	p2	(AT)7	14	13070	13083	LSC
16	p4	(TTTA)4	12	13149	13160	LSC
17	p1	(A)14	14	16388	16401	LSC
18	p1	(T)11	11	18610	18620	LSC
19	p2	(AT)5	10	19974	19983	LSC
20	p1	(T)11	11	21053	21063	LSC
21	p4	(AAAT)3	12	22815	22826	LSC
22	p1	(T)12	12	26355	26366	LSC
23	p1	(A)10	10	27372	27381	LSC
24	p1	(T)11	11	29577	29587	LSC
25	p2	(AT)5	10	30087	30096	LSC
26	p1	(T)10	10	32722	32731	LSC
27	p1	(T)11	11	33067	33077	LSC
28	p1	(A)12	12	33271	33282	LSC
29	p5	(TTTTA)3	15	37809	37823	LSC
30	p4	(ATTT)3	12	42856	42867	LSC
31	p1	(A)11	11	43387	43397	LSC
32	p1	(A)10	10	43420	43429	LSC
33	p1	(T)10	10	45229	45238	LSC
34	p1	(A)11	11	45611	45621	LSC
35	p1	(T)12	12	49103	49114	LSC
36	p2	(AT)5	10	50235	50244	LSC
37	p2	(TA)5	10	51883	51892	LSC
38	p1	(T)13	13	52634	52646	LSC
39	p1	(T)10	10	55589	55598	LSC
40	p3	(ATA)4	12	55643	55654	LSC
41	p1	(A)12	12	56174	56185	LSC
42	p1	(T)12	12	57955	57966	LSC
43	p1	(T)10	10	60460	60469	LSC
44	p1	(T)13	13	60701	60713	LSC
45	p1	(A)10	10	63875	63884	LSC
46	p2	(TA)5	10	63904	63913	LSC
47	p5	(TATAT)3	15	66771	66785	LSC
48	p2	(TA)5	10	66781	66790	LSC
49	p4	(TCCT)3	12	67836	67847	LSC
50	p1	(A)12	12	68712	68723	LSC
51	p1	(T)12	12	70846	70857	LSC
52	p1	(T)12	12	72408	72419	LSC
53	p2	(AT)5	10	77486	77495	LSC
54	p1	(T)10	10	80940	80949	LSC

	55	p4	(TTTC)3	12	81034	81045	LSC
	56	p1	(A)10	10	81507	81516	LSC
	57	p2	(AT)5	10	84256	84265	LSC
	58	p1	(T)11	11	84575	84585	LSC
	59	p1	(G)13	13	94072	94084	IRb
	60	p1	(T)17	17	99119	99135	IRb
	61	p1	(T)10	10	103108	103117	IRb
	62	p1	(G)12	12	104329	104340	IRb
	63	p4	(AGGT)3	12	106418	106429	IRb
	64	p1	(T)10	10	108591	108600	IRb
	65	p1	(A)10	10	110959	110968	IRb
	66	p1	(A)11	11	112135	112145	SSC
	67	p1	(A)10	10	114531	114540	SSC
	68	p2	(TA)9	9	114610	114618	SSC
	69	p2	(TA)6	12	115224	115235	SSC
	70	p2	(AT)6	12	115258	115269	SSC
	71	p4	(TCTT)3	12	116280	116291	SSC
	72	p1	(A)12	12	116327	116338	SSC
	73	p1	(T)10	10	117228	117237	SSC
	74	p2	(TA)5	10	120482	120491	SSC
	75	p1	(T)12	12	120671	120682	SSC
	76	p4	(CAAT)3	12	120746	120757	SSC
	77	p2	(TA)5	10	127081	127090	SSC
	78	p1	(T)13	13	127601	127613	SSC
	79	p1	(A)10	10	127652	127661	SSC
	80	p1	(T)10	10	128272	128281	SSC
	81	p1	(T)11	11	128816	128826	SSC
	82	p1	(T)10	10	129577	129586	IRa
	83	p1	(A)10	10	131945	131954	IRa
	84	p4	(CTAC)3	12	134114	134125	IRa
	85	p1	(C)12	12	136205	136216	IRa
	86	p1	(A)10	10	137428	137437	IRa
	87	p1	(A)17	17	141410	141426	IRa
	88	p1	(C)13	13	146461	146473	IRa
L. lanzhouensis	1	p4	(AATA)3	12	3883	3894	LSC
	2	p1	(A)10	10	4222	4231	LSC
	3	p2	(TA)5	10	4814	4823	LSC
	4	p1	(T)10	10	5479	5488	LSC
	5	p5	(ATTTT)3	15	6320	6334	LSC
	6	p1	(T)10	10	8117	8126	LSC
	7	p1	(G)12	12	8780	8791	LSC
	8	p1	(T)11	11	8884	8894	LSC
	9	p1	(T)10	10	8917	8926	LSC
	10	p2	(AT)5	10	9852	9861	LSC
	11	p2	(AT)5	10	10054	10063	LSC
	12	p1	(A)10	10	13006	13015	LSC
	13	p2	(AT)9	18	13065	13082	LSC
	14	p4	(TTTA)4	16	13144	13159	LSC

15	p1	(A)10	10	13554	13563	LSC
16	p1	(A)15	15	16388	16402	LSC
17	p1	(T)11	11	18611	18621	LSC
18	p2	(AT)5	10	19975	19984	LSC
19	p1	(T)11	11	21054	21064	LSC
20	p4	(AAAT)3	12	22801	22812	LSC
21	p1	(T)12	12	26341	26352	LSC
22	p1	(C)10	10	29391	29400	LSC
23	p1	(T)12	12	29573	29584	LSC
24	p2	(AT)5	10	30069	30078	LSC
25	p2	(AT)7	14	31754	31767	LSC
26	p1	(T)11	11	32739	32749	LSC
27	p1	(A)10	10	33286	33295	LSC
28	p1	(C)10	10	37088	37097	LSC
29	p5	(TTTTA)3	15	37821	37835	LSC
30	p4	(ATTT)3	12	42868	42879	LSC
31	p1	(A)11	11	43430	43440	LSC
32	p1	(T)10	10	45242	45251	LSC
33	p1	(A)12	12	45621	45632	LSC
34	p2	(AT)5	10	50258	50267	LSC
35	p2	(TA)5	10	51900	51909	LSC
36	p2	(TA)5	10	51931	51940	LSC
37	p2	(TA)5	10	51962	51971	LSC
38	p2	(TA)5	10	51993	52002	LSC
39	p1	(T)10	10	55693	55702	LSC
40	p3	(ATA)4	12	55747	55758	LSC
41	p1	(T)10	10	55781	55790	LSC
42	p1	(A)13	13	56279	56291	LSC
43	p1	(T)11	11	58061	58071	LSC
44	p1	(T)13	13	60580	60592	LSC
45	p1	(T)10	10	60831	60840	LSC
46	p1	(A)14	14	64180	64193	LSC
47	p2	(TA)5	10	64213	64222	LSC
48	p4	(TCCT)3	12	68103	68114	LSC
49	p1	(T)12	12	72638	72649	LSC
50	p2	(AT)5	10	77695	77704	LSC
51	p1	(T)11	11	81150	81160	LSC
52	p1	(T)10	10	83121	83130	LSC
53	p2	(AT)5	10	84504	84513	LSC
54	p1	(G)11	11	94394	94404	IRb
55	p1	(T)14	14	99439	99452	IRb
56	p1	(A)10	10	103325	103334	IRb
57	p1	(T)11	11	103427	103437	IRb
58	p1	(G)15	15	104649	104663	IRb
59	p4	(AGGT)3	12	106741	106752	IRb
60	p1	(A)10	10	111279	111288	IRb
61	p2	(TA)7	14	114900	114913	SSC
62	p5	(TATAT)3	15	114941	114955	SSC
63	p2	(AT)7	14	115562	115575	SSC

	64	p4	(TCTT)3	12	116576	116587	SSC
	65	p1	(A)12	12	116623	116634	SSC
	66	p2	(TA)5	10	120756	120765	SSC
	67	p4	(CAAT)3	12	121016	121027	SSC
	68	p5	(ATAGT)3	15	123105	123119	SSC
	69	p2	(TA)5	10	127351	127360	SSC
	70	p1	(T)19	19	127871	127889	SSC
	71	p1	(T)10	10	128548	128557	SSC
	72	p1	(T)11	11	129092	129102	SSC
	73	p1	(T)10	10	129853	129862	IRa
	74	p4	(CTAC)3	12	134387	134398	IRa
	75	p1	(C)15	15	136478	136492	IRa
	76	p1	(A)11	11	137704	137714	IRa
	77	p1	(T)10	10	137807	137816	IRa
	78	p1	(A)14	14	141689	141702	IRa
	79	p1	(C)11	11	146737	146747	IRa
L. schrenkiana	1	p4	(TTTA)3	12	160	171	LSC
	2	p1	(A)10	10	195	204	LSC
	3	p1	(A)11	11	1573	1583	LSC
	4	p4	(AATA)3	12	3905	3916	LSC
	5	p1	(A)19	19	4244	4262	LSC
	6	p2	(TA)5	10	4842	4851	LSC
	7	p3	(TTA)5	15	5450	5464	LSC
	8	p1	(A)10	10	8260	8269	LSC
	9	p1	(T)11	11	8529	8539	LSC
	10	p1	(A)11	11	9012	9022	LSC
	11	p2	(AT)5	10	9472	9481	LSC
	12	p2	(AT)5	10	9667	9676	LSC
	13	p1	(T)10	10	12004	12013	LSC
	14	p1	(A)10	10	12621	12630	LSC
	15	p1	(C)11	11	12658	12668	LSC
	16	p2	(AT)7	14	12682	12695	LSC
	17	p4	(TTTA)3	12	12757	12768	LSC
	18	p5	(ATAGC)3	15	13660	13674	LSC
	19	p1	(A)11	11	16004	16014	LSC
	20	p1	(T)11	11	18223	18233	LSC
	21	p2	(AT)5	10	19587	19596	LSC
	22	p4	(AAAT)3	12	22412	22423	LSC
	23	p1	(T)12	12	25952	25963	LSC
	24	p1	(A)10	10	26969	26978	LSC
	25	p3	(AAT)4	12	28284	28295	LSC
	26	p1	(T)13	13	29240	29252	LSC
	27	p2	(AT)5	10	29733	29742	LSC
	28	p1	(A)13	13	32478	32490	LSC
	29	p1	(A)10	10	36694	36703	LSC
	30	p4	(ATTT)3	12	42007	42018	LSC
	31	p1	(A)11	11	42527	42537	LSC
	32	p2	(AT)5	10	42541	42550	LSC

33	p1	(T)10	10	44396	44405	LSC
34	p1	(A)14	14	44778	44791	LSC
35	p2	(TA)12	24	46609	46632	LSC
36	p2	(TA)5	10	46636	46645	LSC
37	p2	(TA)5	10	46662	46671	LSC
38	p2	(AT)5	10	49454	49463	LSC
39	p1	(T)10	10	51864	51873	LSC
40	p1	(T)10	10	54824	54833	LSC
41	p3	(ATA)4	12	54878	54889	LSC
42	p1	(A)12	12	55418	55429	LSC
43	p1	(T)10	10	57199	57208	LSC
44	p1	(T)11	11	59736	59746	LSC
45	p2	(TA)5	10	63355	63364	LSC
46	p1	(T)10	10	65527	65536	LSC
47	p2	(TA)5	10	66241	66250	LSC
48	p4	(TCCT)3	12	67285	67296	LSC
49	p1	(T)10	10	69301	69310	LSC
50	p2	(TA)5	10	70172	70181	LSC
51	p1	(A)10	10	71095	71104	LSC
52	p6	(ATATTA)3	18	71363	71380	LSC
53	p1	(T)11	11	71833	71843	LSC
54	p2	(AT)5	10	76923	76932	LSC
55	p1	(T)15	15	82340	82354	LSC
56	p2	(AT)5	10	83722	83731	LSC
57	p1	(T)16	16	84042	84057	LSC
58	p1	(G)10	10	93635	93644	IRb
59	p1	(T)14	14	98701	98714	IRb
60	p1	(T)10	10	102687	102696	IRb
61	p1	(G)11	11	103928	103938	IRb
62	p4	(AGGT)3	12	106016	106027	IRb
63	p1	(A)10	10	110564	110573	IRb
64	p2	(TA)15	30	114477	114506	SSC
65	p2	(AT)7	14	114521	114534	SSC
66	p4	(TCTT)3	12	115552	115563	SSC
67	p1	(A)13	13	115599	115611	SSC
68	p1	(A)12	12	119128	119139	SSC
69	p2	(TA)5	10	119737	119746	SSC
70	p1	(T)11	11	119926	119936	SSC
71	p4	(CAAT)3	12	120000	120011	SSC
72	p2	(TA)5	10	126320	126329	SSC
73	p1	(T)15	15	126847	126861	SSC
74	p1	(T)10	10	127472	127481	SSC
75	p1	(T)11	11	127989	127999	SSC
76	p1	(T)10	10	128774	128783	IRa
77	p4	(CTAC)3	12	133318	133329	IRa
78	p1	(C)11	11	135409	135419	IRa
79	p1	(A)10	10	136651	136660	IRa
80	p1	(A)14	14	140633	140646	IRa
81	p1	(C)10	10	145703	145712	IRa

L. seseloides	1	p4	(TAAA)3	12	186	197	LSC
	2	p4	(AATA)3	12	3895	3906	LSC
	3	p1	(A)16	16	4236	4251	LSC
	4	p2	(TA)5	10	4830	4839	LSC
	5	p1	(T)11	11	8147	8157	LSC
	6	p1	(T)10	10	8953	8962	LSC
	7	p1	(A)10	10	9435	9444	LSC
	8	p2	(AT)5	10	9907	9916	LSC
	9	p2	(AT)5	10	10114	10123	LSC
	10	p1	(T)10	10	12451	12460	LSC
	11	p2	(AT)5	10	13133	13142	LSC
	12	p4	(TTAT)3	12	13212	13223	LSC
	13	p1	(A)12	12	16520	16531	LSC
	14	p1	(T)11	11	18740	18750	LSC
	15	p2	(AT)5	10	20104	20113	LSC
	16	p1	(A)10	10	22786	22795	LSC
	17	p1	(T)11	11	22917	22927	LSC
	18	p4	(AAAT)3	12	22937	22948	LSC
	19	p1	(T)12	12	26477	26488	LSC
	20	p3	(AAT)4	12	28768	28779	LSC
	21	p1	(C)10	10	29540	29549	LSC
	22	p1	(T)12	12	29722	29733	LSC
	23	p2	(AT)5	10	30220	30229	LSC
	24	p1	(A)10	10	31095	31104	LSC
	25	p1	(T)12	12	32790	32801	LSC
	26	p1	(A)10	10	33348	33357	LSC
	27	p3	(TTA)4	12	36770	36781	LSC
	28	p4	(ATTT)3	12	42888	42899	LSC
	29	p1	(A)11	11	43439	43449	LSC
	30	p1	(T)10	10	45267	45276	LSC
	31	p1	(A)13	13	45649	45661	LSC
	32	p2	(AT)5	10	50218	50227	LSC
	33	p2	(TA)5	10	51862	51871	LSC
	34	p1	(T)10	10	52621	52630	LSC
	35	p1	(T)10	10	55581	55590	LSC
	36	p3	(ATA)4	12	55635	55646	LSC
	37	p1	(A)12	12	56166	56177	LSC
	38	p1	(T)10	10	57947	57956	LSC
	39	p2	(AT)5	10	60259	60268	LSC
	40	p1	(T)14	14	60454	60467	LSC
	41	p1	(A)11	11	64050	64060	LSC
	42	p2	(TA)5	10	64084	64093	LSC
	43	p2	(TA)5	10	66945	66954	LSC
	44	p4	(TCCT)3	12	67982	67993	LSC
	45	p1	(A)10	10	71782	71791	LSC
	46	p1	(T)11	11	72530	72540	LSC
	47	p2	(AT)5	10	77621	77630	LSC
	48	p1	(T)12	12	81056	81067	LSC

	49	p1	(A)11	11	81625	81635	LSC
	50	p1	(T)10	10	81710	81719	LSC
	51	p1	(T)14	14	83016	83029	LSC
	52	p2	(AT)5	10	84397	84406	LSC
	53	p1	(T)21	21	84717	84737	LSC
	54	p1	(G)12	12	94239	94250	IRb
	55	p1	(T)12	12	99261	99272	IRb
	56	p1	(T)10	10	103261	103270	IRb
	57	p1	(G)12	12	104482	104493	IRb
	58	p4	(AGGT)3	12	106571	106582	IRb
	59	p1	(A)10	10	111091	111100	IRb
	60	p1	(A)11	11	111875	111885	IRb
	61	p2	(AT)10	20	115452	115471	SSC
	62	p2	(TA)7	14	115652	115665	SSC
	63	p2	(AT)5	10	115680	115689	SSC
	64	p2	(TA)6	12	115690	115701	SSC
	65	p4	(TCTT)3	12	116704	116715	SSC
	66	p1	(A)13	13	116751	116763	SSC
	67	p1	(A)15	15	120277	120291	SSC
	68	p2	(TA)5	10	120889	120898	SSC
	69	p4	(CAAT)3	12	121149	121160	SSC
	70	p2	(TA)5	10	127463	127472	SSC
	71	p1	(T)15	15	127990	128004	SSC
	72	p1	(T)10	10	128615	128624	SSC
	73	p1	(T)11	11	129132	129142	IRa
	74	p1	(T)10	10	129917	129926	IRa
	75	p4	(CTAC)3	12	134433	134444	IRa
	76	p1	(C)12	12	136524	136535	IRa
	77	p1	(A)10	10	137747	137756	IRa
	78	p1	(A)12	12	141745	141756	IRa
	79	p1	(C)12	12	146767	146778	IRa
L. sibirica	1	p4	(TTTA)3	12	160	171	LSC
	2	p1	(A)11	11	195	205	LSC
	3	p4	(AATA)3	12	3905	3916	LSC
	4	p1	(A)19	19	4244	4262	LSC
	5	p2	(TA)5	10	4842	4851	LSC
	6	p3	(TTA)5	15	5450	5464	LSC
	7	p1	(A)10	10	8260	8269	LSC
	8	p1	(T)12	12	8529	8540	LSC
	9	p1	(A)11	11	9013	9023	LSC
	10	p2	(AT)5	10	9473	9482	LSC
	11	p2	(AT)5	10	9668	9677	LSC
	12	p1	(T)10	10	12005	12014	LSC
	13	p1	(A)10	10	12622	12631	LSC
	14	p2	(AT)7	14	12680	12693	LSC
	15	p4	(TTTA)3	12	12755	12766	LSC
	16	p5	(ATAGC)3	15	13658	13672	LSC
	17	p1	(A)10	10	16002	16011	LSC

18	p1	(T)11	11	18220	18230	LSC
19	p2	(AT)5	10	19584	19593	LSC
20	p4	(AAAT)3	12	22409	22420	LSC
21	p1	(T)12	12	25949	25960	LSC
22	p1	(A)10	10	26966	26975	LSC
23	p3	(AAT)4	12	28256	28267	LSC
24	p1	(T)13	13	29212	29224	LSC
25	p2	(AT)5	10	29699	29708	LSC
26	p1	(A)12	12	32512	32523	LSC
27	p1	(A)10	10	36727	36736	LSC
28	p4	(ATTT)3	12	42040	42051	LSC
29	p1	(A)11	11	42560	42570	LSC
30	p2	(AT)5	10	42574	42583	LSC
31	p1	(A)10	10	42595	42604	LSC
32	p1	(T)10	10	44408	44417	LSC
33	p1	(A)17	17	44790	44806	LSC
34	p2	(TA)12	24	46632	46665	LSC
35	p2	(TA)5	10	46659	46668	LSC
36	p2	(AT)5	10	49444	49453	LSC
37	p2	(TA)5	10	51093	51102	LSC
38	p1	(T)11	11	51853	51863	LSC
39	p1	(T)10	10	54814	54823	LSC
40	p3	(ATA)4	12	54868	54879	LSC
41	p1	(A)11	11	55416	55426	LSC
42	p1	(T)10	10	57196	57205	LSC
43	p1	(T)11	11	59718	59728	LSC
44	p2	(TA)5	10	63337	63346	LSC
45	p2	(TA)6	12	63348	63359	LSC
46	p1	(T)11	11	65525	65535	LSC
47	p2	(TA)5	10	66240	66249	LSC
48	p4	(TCCT)3	12	67276	67287	LSC
49	p1	(T)10	10	69292	69301	LSC
50	p2	(TA)5	10	70163	70172	LSC
51	p6	(ATATTA)3	18	71361	71378	LSC
52	p1	(T)11	11	71831	71841	LSC
53	p2	(AT)5	10	76921	76930	LSC
54	p1	(T)14	14	82339	82352	LSC
55	p2	(AT)5	10	83720	83729	LSC
56	p1	(T)17	17	84040	84056	LSC
57	p1	(G)10	10	93536	93545	IRb
58	p1	(T)14	14	98623	98636	IRb
59	p1	(T)10	10	102609	102618	IRb
60	p1	(G)12	12	103850	103861	IRb
61	p4	(AGGT)3	12	105939	105950	IRb
62	p1	(A)10	10	110487	110496	IRb
63	p2	(TA)11	22	114404	114425	SSC
64	p2	(AT)7	14	114440	114453	SSC
65	p4	(TCTT)3	12	115471	115482	SSC
66	p1	(A)13	13	115518	115530	SSC

	67	p1	(A)12	12	119047	119058	SSC
	68	p2	(TA)5	10	119656	119665	SSC
	69	p1	(T)11	11	119845	119855	SSC
	70	p4	(CAAT)3	12	119919	119930	SSC
	71	p2	(TA)5	10	126239	126248	SSC
	72	p1	(T)15	15	126766	126780	SSC
	73	p1	(T)10	10	127391	127400	SSC
	74	p1	(T)11	11	127908	127918	SSC
	75	p1	(T)10	10	128693	128702	IRa
	76	p4	(CTAC)3	12	133237	133248	IRa
	77	p1	(C)12	12	135328	135339	IRa
	78	p1	(A)10	10	136571	136580	IRa
	79	p1	(A)14	14	140553	140566	IRa
	80	p1	(C)10	10	145644	145653	IRa
L. spodotrichoma	1	p4	(TTTA)3	12	439	450	LSC
	2	p4	(TTTA)3	12	466	477	LSC
	3	p1	(A)10	10	1874	1883	LSC
	4	p1	(A)10	10	2154	2163	LSC
	5	p4	(AATA)3	12	4229	4240	LSC
	6	p1	(A)17	17	4568	4584	LSC
	7	p2	(TA)5	10	5170	5179	LSC
	8	p4	(TATT)3	12	5216	5227	LSC
	9	p1	(T)10	10	7169	7178	LSC
	10	p1	(T)11	11	8301	8311	LSC
	11	p1	(G)11	11	8965	8975	LSC
	12	p1	(T)11	11	9101	9111	LSC
	13	p2	(AT)5	10	10037	10046	LSC
	14	p2	(AT)5	10	10244	10253	LSC
	15	p2	(AT)7	14	13262	13275	LSC
	16	p4	(TTAT)3	12	13345	13356	LSC
	17	p1	(A)11	11	13377	13387	LSC
	18	p1	(A)14	14	16610	16623	LSC
	19	p3	(ATT)4	12	16733	16744	LSC
	20	p1	(T)11	11	18838	18848	LSC
	21	p2	(AT)5	10	20202	20211	LSC
	22	p1	(T)11	11	21281	21291	LSC
	23	p1	(T)12	12	26566	26577	LSC
	24	p1	(T)12	12	29781	29792	LSC
	25	p2	(AT)5	10	30278	30287	LSC
	26	p1	(T)10	10	32217	32226	LSC
	27	p1	(T)11	11	32925	32935	LSC
	28	p1	(A)10	10	33472	33481	LSC
	29	p5	(TTTTA)3	15	37983	37997	LSC
	30	p4	(ATTT)3	12	43030	43041	LSC
	31	p1	(A)16	16	43594	43609	LSC
	32	p1	(T)10	10	45411	45420	LSC
	33	p2	(AT)5	10	50464	50473	LSC
	34	p2	(TA)10	20	52112	52131	LSC

35	p1	(T)12	12	52859	52870	LSC
36	p1	(T)12	12	53042	53053	LSC
37	p1	(T)10	10	55845	55854	LSC
38	p3	(ATA)4	12	55899	55910	LSC
39	p1	(A)11	11	56439	56449	LSC
40	p1	(T)10	10	58219	58228	LSC
41	p1	(T)11	11	60717	60727	LSC
42	p1	(T)12	12	60959	60970	LSC
43	p1	(A)12	12	64310	64321	LSC
44	p2	(TA)6	12	64339	64350	LSC
45	p4	(TCCT)3	12	68235	68246	LSC
46	p1	(T)11	11	71266	71276	LSC
47	p1	(T)10	10	72827	72836	LSC
48	p1	(A)12	12	76139	76150	LSC
49	p2	(AT)5	10	77898	77907	LSC
50	p1	(T)10	10	81359	81368	LSC
51	p1	(T)11	11	83314	83324	LSC
52	p2	(AT)5	10	84692	84701	LSC
53	p1	(G)14	14	93622	93635	IRb
54	p1	(T)12	12	98670	98681	IRb
55	p1	(T)10	10	102655	102664	IRb
56	p1	(G)11	11	103896	103906	IRb
57	p4	(AGGT)3	12	105984	105995	IRb
58	p1	(A)10	10	110516	110525	IRb
59	p1	(A)10	10	114047	114056	SSC
60	p2	(TA)5	10	114110	114119	SSC
61	p1	(T)10	10	114120	114129	SSC
62	p2	(TA)7	14	114731	114744	SSC
63	p2	(AT)5	10	114759	114768	SSC
64	p4	(TCTT)3	12	115778	115789	SSC
65	p1	(A)13	13	115825	115837	SSC
66	p2	(TA)5	10	119941	119950	SSC
67	p1	(T)11	11	120130	120140	SSC
68	p4	(CAAT)3	12	120204	120215	SSC
69	p2	(TA)5	10	126453	126462	SSC
70	p1	(T)16	16	126976	126991	SSC
71	p1	(T)10	10	127650	127659	SSC
72	p1	(T)11	11	128194	128204	SSC
73	p1	(T)10	10	128955	128964	IRa
74	p4	(CTAC)3	12	133483	133494	IRa
75	p1	(C)11	11	135574	135584	IRa
76	p1	(A)10	10	136816	136825	IRa
77	p1	(A)12	12	140799	140810	IRa
78	p1	(C)14	14	145845	145858	IRa

Table S7 The repeat sequences distribution in the thirteen *Libanotis* plastomes. These data were visualized in Figure S1.

**Taxa**	Length of the 1st repeat	Starting site of the 1st repeat	Repeat type	Length of the 2nd repeat	Starting site of the 2nd repeat	Mismatch
L.acaulis	62	70199	P	62	93242	-1
	62	70199	F	62	147681	-1
	48	91284	F	48	91302	-1
	38	70223	P	38	93242	0
	38	70223	F	38	147705	0
	43	86532	F	43	93308	-2
	43	86532	P	43	147634	-2
	45	74759	P	45	74759	-3
	39	30937	P	39	31466	-1
	41	98961	F	41	122250	-2
	41	122250	P	41	141983	-2
	34	29996	P	34	29996	0
	39	44581	F	39	98963	-2
	39	44581	F	39	122252	-2
	39	44581	P	39	141983	-2
	31	115462	R	31	115462	0
	37	115466	R	37	115466	-2
	34	108064	F	34	108096	-1
	34	108064	P	34	132855	-1
	34	108096	P	34	132887	-1
	34	132855	F	34	132887	-1
	30	8401	P	30	46265	0
	36	114888	P	36	114888	-2
	33	98969	F	33	122258	-1
	33	122258	P	33	141983	-1
	36	115462	F	36	115476	-3
	35	20758	F	35	20807	-3
	35	44584	F	35	95894	-3
	35	44584	P	35	145056	-3
	34	91284	F	34	91320	-3
	31	65467	P	31	65500	-2
	31	114832	R	31	114832	-2
	31	115486	R	31	115486	-2
	30	8398	F	30	36335	-2
	31	22791	P	31	22791	-3
	31	108417	P	31	108417	-3
	31	108417	F	31	132537	-3
	31	132537	P	31	132537	-3
	30	36338	P	30	46265	-3
	30	39562	F	30	41786	-3
	30	88854	F	30	88896	-3
	30	90024	F	30	90039	-3
	30	91309	F	30	91327	-3

L. buchtormensis	66	91377	F	66	91395	-1
	48	91377	F	48	91413	-1
	44	90102	F	44	90117	-2
	45	74790	P	45	74790	-3
	39	30944	P	39	31473	-1
	41	98970	F	41	122519	-2
	41	122519	P	41	142164	-2
	37	117824	F	37	117854	-1
	39	44551	F	39	98972	-2
	39	44551	F	39	122521	-2
	39	44551	P	39	142164	-2
	32	30004	P	32	30004	0
	34	108041	F	34	108073	-1
	34	108041	P	34	133068	-1
	34	108073	P	34	133100	-1
	34	133068	F	34	133100	-1
	30	8432	P	30	46236	0
	33	98978	F	33	122527	-1
	33	122527	P	33	142164	-1
	34	91377	F	34	91431	-2
	35	20748	F	35	20797	-3
	35	44554	F	35	95903	-3
	35	44554	P	35	145237	-3
	30	8429	F	30	36335	-2
	31	65485	P	31	65518	-3
	31	108394	P	31	108394	-3
	31	108394	F	31	132750	-3
	31	132750	P	31	132750	-3
	30	9823	F	30	37303	-3
	30	36338	P	30	46236	-3
	30	39519	F	30	41752	-3
	30	45517	P	30	128074	-3
	30	64029	F	30	64048	-3
	30	88932	F	30	88974	-3
L. condensata	66	92390	F	66	92408	-1
	46	48211	F	46	48232	0
	46	64998	F	46	65056	0
	52	92390	F	52	92426	-3
	41	92415	F	41	92433	0
	45	75828	P	45	75828	-3
	39	31745	P	39	32280	-1
	41	99106	F	41	122430	-2
	41	122430	P	41	142148	-2
	39	45417	F	39	99108	-2
	39	45417	F	39	122432	-2
	39	45417	P	39	142148	-2
	32	30806	P	32	30806	0
	34	108219	F	34	108251	-1

	34	108219	P	34	133010	-1
	34	108251	P	34	133042	-1
	34	133010	F	34	133042	-1
	39	66514	P	39	66539	-3
	30	9187	P	30	47123	0
	33	99114	F	33	122438	-1
	33	122438	P	33	142148	-1
	35	114999	R	35	114999	-2
	30	61044	F	30	61070	-1
	35	5600	F	35	5624	-3
	35	21523	F	35	21572	-3
	35	45420	F	35	96040	-3
	35	45420	P	35	145220	-3
	34	92390	F	34	92444	-3
	30	9184	F	30	37173	-2
	30	10630	R	30	10630	-2
	32	115008	F	32	115010	-3
	31	108572	P	31	108572	-3
	31	108572	F	31	132692	-3
	31	132692	P	31	132692	-3
	30	305	F	30	127955	-3
	30	37176	P	30	47123	-3
	30	40384	F	30	42608	-3
	30	44183	P	30	127958	-3
	30	48206	F	30	48248	-3
	30	50558	C	30	65025	-3
	30	89960	F	30	90002	-3
	30	91130	F	30	91145	-3
	30	92415	F	30	92451	-3
L. depressa	1352	92195	P	1352	146177	0
	565	91630	P	565	147535	0
	102	92314	F	102	92533	-3
	102	92314	P	102	147089	-3
	102	92533	P	102	147308	-3
	102	147089	F	102	147308	-3
	93	147098	F	93	147317	-1
	87	92411	F	87	92516	0
	87	92411	P	87	147121	0
	87	92516	P	87	147226	0
	87	147121	F	87	147226	0
	80	92327	F	80	92546	0
	80	92327	P	80	147098	0
	80	92546	P	80	147317	0
	75	92408	F	75	92629	-1
	75	92408	P	75	147020	-1
	75	92629	P	75	147241	-1
	75	147020	F	75	147241	-1
	72	92516	F	72	92632	-1

	72	92516	P	72	147020	-1
	72	92632	P	72	147136	-1
	72	147020	F	72	147136	-1
	70	92314	F	70	92428	-1
	70	92314	P	70	147226	-1
	70	92428	P	70	147340	-1
	70	147226	F	70	147340	-1
	68	109129	F	68	109633	-1
	68	109129	P	68	130029	-1
	68	109633	P	68	130533	-1
	68	130029	F	68	130533	-1
	57	92327	F	57	92441	0
	57	92327	P	57	147226	0
	57	92441	P	57	147340	0
	51	130046	F	51	130550	0
	59	90403	F	59	90421	-3
	55	92314	F	55	92649	-2
	55	92314	P	55	147020	-2
	55	92649	P	55	147355	-2
	55	147020	F	55	147355	-2
	46	147049	F	46	147270	0
	43	147049	F	43	147165	0
	49	90417	F	49	90435	-3
	42	92327	F	42	92662	-1
	42	92327	P	42	147020	-1
	42	92662	P	42	147355	-1
	37	105250	R	37	105250	0
	37	105250	C	37	134443	0
	37	134443	R	37	134443	0
	40	92255	F	40	92489	-1
L. grubovii	66	91300	F	66	91318	-1
	52	91300	F	52	91336	-3
	41	91325	F	41	91343	0
	39	30957	P	39	31486	0
	45	64038	P	45	64038	-3
	45	74771	P	45	74771	-3
	42	86549	F	42	93256	-2
	42	86549	P	42	147406	-2
	41	98924	F	41	122043	-2
	41	122043	P	41	141739	-2
	34	30025	P	34	30025	0
	34	93787	F	34	93802	0
	34	93787	P	34	146868	0
	34	93802	P	34	146883	0
	34	146868	F	34	146883	0
	39	44600	F	39	98926	-2
	39	44600	F	39	122045	-2
	39	44600	P	39	141739	-2

	34	108011	F	34	108043	-1
	34	108011	P	34	132627	-1
	34	108043	P	34	132659	-1
	34	132627	F	34	132659	-1
	30	8375	P	30	46272	0
	33	98932	F	33	122051	-1
	33	122051	P	33	141739	-1
	35	111655	R	35	111655	-2
	37	32353	P	37	32353	-3
	35	20708	F	35	20757	-3
	35	44603	F	35	95857	-3
	35	44603	P	35	144812	-3
	34	91300	F	34	91354	-3
	30	8372	F	30	36364	-2
	30	65483	P	30	65515	-2
	31	32345	P	31	32443	-3
	31	91325	F	31	91361	-3
	31	108364	P	31	108364	-3
	31	108364	F	31	132309	-3
	31	132309	P	31	132309	-3
	30	32652	F	30	127598	-3
	30	36367	P	30	46272	-3
	30	39571	F	30	41795	-3
	30	88870	F	30	88912	-3
	30	90040	F	30	90055	-3
L. iliensis	66	91392	F	66	91410	-1
	52	91392	F	52	91428	-3
	41	91417	F	41	91435	0
	39	31062	P	39	31591	0
	45	74862	P	45	74862	-3
	42	86641	F	42	93348	-2
	42	86641	P	42	147530	-2
	41	99002	F	41	122163	-2
	41	122163	P	41	141877	-2
	34	30124	P	34	30124	0
	39	44678	F	39	99004	-2
	39	44678	F	39	122165	-2
	39	44678	P	39	141877	-2
	34	108108	F	34	108140	-1
	34	108108	P	34	132746	-1
	34	108140	P	34	132778	-1
	34	132746	F	34	132778	-1
	30	8416	P	30	46352	0
	33	99010	F	33	122171	-1
	33	122171	P	33	141877	-1
	37	32451	P	37	32451	-3
	35	20778	F	35	20827	-3
	35	44681	F	35	95935	-3

	35	44681	P	35	144950	-3
	34	91392	F	34	91446	-3
	31	65610	P	31	65643	-2
	30	8413	F	30	36453	-2
	30	64175	R	30	64175	-2
	31	32443	P	31	32541	-3
	31	108461	P	31	108461	-3
	31	108461	F	31	132428	-3
	31	132428	P	31	132428	-3
	30	9877	F	30	9901	-3
	30	36456	P	30	46352	-3
	30	39657	F	30	41881	-3
	30	88962	F	30	89004	-3
	30	90132	F	30	90147	-3
	30	91417	F	30	91453	-3
L. incana	52	91116	F	52	91134	-3
	42	93376	F	42	93415	0
	42	93376	P	42	146665	0
	42	93415	P	42	146704	0
	42	146665	F	42	146704	0
	39	30705	P	39	31234	0
	45	74568	P	45	74568	-3
	41	98760	F	41	121665	-2
	41	121665	P	41	141321	-2
	40	91128	F	40	91146	-2
	33	66675	R	33	66675	0
	39	44300	F	39	98762	-2
	39	44300	F	39	121667	-2
	39	44300	P	39	141321	-2
	32	29765	P	32	29765	0
	34	107853	F	34	107885	-1
	34	107853	P	34	132203	-1
	34	107885	P	34	132235	-1
	34	132203	F	34	132235	-1
	30	8085	P	30	45997	0
	33	98768	F	33	121673	-1
	33	121673	P	33	141321	-1
	35	20468	F	35	20517	-3
	35	44303	F	35	95684	-3
	35	44303	P	35	144403	-3
	31	65262	P	31	65295	-2
	30	8082	F	30	36100	-2
	30	118979	F	30	118991	-2
	32	43033	P	32	45835	-3
	31	63823	P	31	63823	-3
	31	108206	P	31	108206	-3
	31	108206	F	31	131885	-3
	31	113930	F	31	113962	-3

	31	131885	P	31	131885	-3
	30	9478	F	30	37070	-3
	30	36103	P	30	45997	-3
	30	39283	F	30	41507	-3
	30	88686	F	30	88728	-3
	30	89856	F	30	89871	-3
	30	91116	F	30	91152	-3
	30	91141	F	30	91159	-3
L. jinanensis	84	91170	F	84	91188	-3
	66	91170	F	66	91206	-1
	59	91195	F	59	91213	-1
	52	91170	F	52	91224	-3
	41	91213	F	41	91231	0
	45	74661	P	45	74661	-3
	41	98744	F	41	122017	-2
	41	122017	P	41	141759	-2
	39	31013	P	39	31542	-2
	39	44639	F	39	98746	-2
	39	44639	F	39	122019	-2
	39	44639	P	39	141759	-2
	32	30075	P	32	30075	0
	34	107826	F	34	107858	-1
	34	107826	P	34	132652	-1
	34	107858	P	34	132684	-1
	34	132652	F	34	132684	-1
	30	8380	P	30	46319	0
	33	98752	F	33	122025	-1
	33	122025	P	33	141759	-1
	34	91170	F	34	91242	-2
	35	20747	F	35	20796	-3
	35	44642	F	35	95678	-3
	35	44642	P	35	144831	-3
	34	103	P	34	109	-3
	31	65348	P	31	65381	-2
	30	8377	F	30	36392	-2
	30	128389	F	30	128416	-2
	32	29639	C	32	31722	-3
	31	108179	P	31	108179	-3
	31	108179	F	31	132334	-3
	31	110247	F	31	110265	-3
	31	110247	P	31	130248	-3
	31	110265	P	31	130266	-3
	31	130248	F	31	130266	-3
	31	132334	P	31	132334	-3
	30	36395	P	30	46319	-3
	30	39615	F	30	41839	-3
	30	44648	F	30	82043	-3
	30	88740	F	30	88782	-3

	30	89910	F	30	89925	-3
	30	91213	F	30	91249	-3
L. lanzhouensis	120	91425	F	120	91443	-1
	102	91425	F	102	91461	-1
	94	51887	F	94	51918	0
	84	91425	F	84	91479	-1
	63	51887	F	63	51949	0
	66	91425	F	66	91497	-1
	48	91425	F	48	91515	-1
	44	90150	F	44	90165	-2
	45	74878	P	45	74878	-3
	39	30996	P	39	31525	-1
	41	99064	F	41	122294	-2
	41	122294	P	41	142035	-2
	39	44652	F	39	99066	-2
	39	44652	F	39	122296	-2
	39	44652	P	39	142035	-2
	32	30057	P	32	30057	0
	32	51887	F	32	51980	0
	34	108149	F	34	108181	-1
	34	108149	P	34	132925	-1
	34	108181	P	34	132957	-1
	34	132925	F	34	132957	-1
	30	8403	P	30	46348	0
	33	99072	F	33	122302	-1
	33	122302	P	33	142035	-1
	34	91425	F	34	91533	-2
	35	20748	F	35	20797	-3
	35	44655	F	35	95998	-3
	35	44655	P	35	145107	-3
	32	114931	P	32	114931	-2
	30	6103	F	30	6117	-2
	30	8400	F	30	36405	-2
	31	7	F	31	27	-3
	31	65657	P	31	65690	-3
	31	108502	P	31	108502	-3
	31	108502	F	31	132607	-3
	31	110567	F	31	110585	-3
	31	110567	P	31	130524	-3
	31	110585	P	31	130542	-3
	31	130524	F	31	130542	-3
	31	132607	P	31	132607	-3
	30	36408	P	30	46348	-3
	30	39627	F	30	41851	-3
	30	44661	F	30	82263	-3
	30	88980	F	30	89022	-3
	30	90150	F	30	90180	-3
	30	128665	F	30	128692	-3

L. schrenkiana	66	90635	F	66	90653	-1
	52	90635	F	52	90671	-3
	42	92933	F	42	92972	0
	42	92933	P	42	146332	0
	42	92972	P	42	146371	0
	42	146332	F	42	146371	0
	41	90660	F	41	90678	0
	49	46622	F	49	46648	-3
	45	74093	P	45	74093	-3
	41	98326	F	41	121278	-2
	41	121278	P	41	140979	-2
	34	107781	F	34	107815	0
	34	107781	P	34	131497	0
	34	107815	P	34	131531	0
	34	131497	F	34	131531	0
	39	30660	P	39	31189	-2
	39	43782	F	39	98328	-2
	39	43782	F	39	121280	-2
	39	43782	P	39	140979	-2
	32	29721	P	32	29721	0
	34	107424	F	34	107456	-1
	34	107424	P	34	131856	-1
	34	107456	P	34	131888	-1
	34	131856	F	34	131888	-1
	30	8006	P	30	45489	0
	30	114476	P	30	114476	0
	36	46597	R	36	46608	-2
	36	114472	P	36	114472	-2
	33	98334	F	33	121286	-1
	33	121286	P	33	140979	-1
	32	92456	F	32	92482	-1
	32	92456	P	32	146832	-1
	32	92482	P	32	146858	-1
	32	146832	F	32	146858	-1
	37	46608	C	37	46609	-3
	37	46608	C	37	114481	-3
	34	46597	R	34	46608	-2
	34	114470	F	34	114472	-2
	31	114476	C	31	114479	-1
	36	46597	P	36	46609	-3
	36	46597	P	36	114481	-3
	36	46597	R	36	114482	-3
	36	46608	F	36	46610	-3
	30	46603	F	30	114471	-1
	30	114475	C	30	114476	-1
	30	114475	R	30	114476	-1
	30	114476	R	30	114477	-1
	30	114476	P	30	114478	-1

	30	114476	R	30	114479	-1
L. seseloides	66	13822	F	66	13843	0
	45	13822	F	45	13864	0
	48	91315	F	48	91333	-1
	45	74773	P	45	74773	-3
	39	31148	P	39	31677	-1
	41	98911	F	41	122427	-2
	41	122427	P	41	142064	-2
	39	44669	F	39	98913	-2
	39	44669	F	39	122429	-2
	39	44669	P	39	142064	-2
	32	30208	P	32	30208	0
	34	107979	F	34	108011	-1
	34	107979	P	34	132971	-1
	34	108011	P	34	133003	-1
	34	132971	F	34	133003	-1
	30	8433	P	30	46326	0
	30	47515	P	30	47515	0
	33	98919	F	33	122435	-1
	33	122435	P	33	142064	-1
	35	20877	F	35	20926	-3
	35	44672	F	35	95844	-3
	35	44672	P	35	145137	-3
	34	91315	F	34	91351	-3
	31	65524	P	31	65557	-2
	31	115448	R	31	115448	-2
	30	8430	F	30	36467	-2
	31	9897	F	31	115670	-3
	31	108332	P	31	108332	-3
	31	108332	F	31	132653	-3
	31	115439	C	31	115440	-3
	31	132653	P	31	132653	-3
	30	9846	F	30	37434	-3
	30	36470	P	30	46326	-3
	30	39647	F	30	41871	-3
	30	88885	F	30	88927	-3
	30	90055	F	30	90070	-3
	30	91340	F	30	91358	-3
L. sibirica	48	90619	F	48	90637	-1
	43	97480	F	43	97501	0
	43	97480	P	43	141644	0
	43	97501	P	43	141665	0
	43	141644	F	43	141665	0
	45	74091	P	45	74091	-3
	41	98248	F	41	121197	-2
	41	121197	P	41	140899	-2
	34	107704	F	34	107738	0

	34	107704	P	34	131416	0
	34	107738	P	34	131450	0
	34	131416	F	34	131450	0
	39	30626	P	39	31155	-2
	39	43817	F	39	98250	-2
	39	43817	F	39	121199	-2
	39	43817	P	39	140899	-2
	32	29687	P	32	29687	0
	34	107347	F	34	107379	-1
	34	107347	P	34	131775	-1
	34	107379	P	34	131807	-1
	34	131775	F	34	131807	-1
	30	8006	P	30	45504	0
	36	46620	R	36	46631	-2
	33	98256	F	33	121205	-1
	33	121205	P	33	140899	-1
	37	46628	F	37	114400	-3
	37	46631	C	37	46632	-3
	34	46620	R	34	46631	-2
	36	46620	P	36	46632	-3
	36	46631	F	36	46633	-3
	35	20357	F	35	20406	-3
	35	43820	F	35	95139	-3
	35	43820	P	35	144014	-3
	35	46620	C	35	46621	-3
	35	46620	P	35	46631	-3
	32	46645	F	32	46658	-2
	32	51451	F	32	51477	-2
	34	46620	F	34	46622	-3
	34	46620	R	34	114403	-3
	34	46634	C	34	114403	-3
	34	90619	F	34	90655	-3
	31	64802	P	31	64835	-2
	33	46620	P	33	46631	-3
	33	46620	P	33	114403	-3
	30	8003	F	30	35644	-2
	31	256	P	31	289	-3
	31	63355	P	31	63355	-3
	31	107700	P	31	107700	-3
	31	107700	F	31	131457	-3
L. spodotrichoma	48	91598	F	48	91616	-1
	45	75077	P	45	75077	-3
	39	31213	P	39	31742	-1
	41	98295	F	41	121482	-2
	41	121482	P	41	141143	-2
	39	44821	F	39	98297	-2
	39	44821	F	39	121484	-2
	39	44821	P	39	141143	-2

32	30266	P	32	30266	0
34	107392	F	34	107424	-1
34	107392	P	34	132021	-1
34	107424	P	34	132053	-1
34	132021	F	34	132053	-1
30	8588	P	30	46500	0
33	98303	F	33	121490	-1
33	121490	P	33	141143	-1
34	91598	F	34	91634	-2
35	20975	F	35	21024	-3
35	44824	F	35	95229	-3
35	44824	P	35	144215	-3
31	52106	R	31	52106	-2
31	65757	P	31	65790	-2
30	8585	F	30	36586	-2
31	107745	P	31	107745	-3
31	107745	F	31	131703	-3
31	109804	F	31	109822	-3
31	109804	P	31	129626	-3
31	109822	P	31	129644	-3
31	129626	F	31	129644	-3
31	131703	P	31	131703	-3
30	36589	P	30	46500	-3
30	39789	F	30	42013	-3
30	44830	F	30	82461	-3
30	90338	F	30	90353	-3
30	127767	F	30	127794	-3

Table S8 Nucleotide diversity (Pi) values of thirteen *Libanotis*, while coding and non-coding regions were listed on the left and right, respectively. These data were visualized in Figure 4.

Coding region	regions	Pi	Non-coding region	regions	Pi
psbA	LSC	0.00232	*trnH-GUG-psbA*	LSC	0.05024
matK	LSC	0.00791	*psbA-trnK-UUU*	LSC	0.00577
rps16	LSC	0.00298	*trnK-UUU-matK*	LSC	0.00807
atpA	LSC	0.00226	*trnK-UUU-rps16*	LSC	0.00632
atpF	LSC	0.00407	*rps16 intron*	LSC	0.00624
atpH	LSC	0.00249	*rps16-trnQ-UUG*	LSC	0.01102
atpI	LSC	0.00370	*trnQ-UUG-psbK*	LSC	0.00849
rps2	LSC	0.00200	*psbK-psbI*	LSC	0.01023
rpoC2	LSC	0.00373	*trnS-GCU-trnG-UCC*	LSC	0.00799
rpoC1	LSC	0.00321	*trnG-UCC intron*	LSC	0.00840
rpoB	LSC	0.00271	*atpF intron*	LSC	0.00457
psbD	LSC	0.00044	*atpF-atpH*	LSC	0.00565
psbC	LSC	0.00148	*atpH-atpI*	LSC	0.00835
rps14	LSC	0.00112	*atpI-rps2*	LSC	0.00488
psaB	LSC	0.00158	*rps2-rpoC2*	LSC	0.00792
psaA	LSC	0.00168	*rpoC2-rpoC1*	LSC	0.00460
ycf3	LSC	0.00305	*rpoC1 intron*	LSC	0.00417
rps4	LSC	0.00111	*rpoB-trnC-GCA*	LSC	0.00781
ndhJ	LSC	0.00269	*trnC-GCA-petN*	LSC	0.00681
ndhK	LSC	0.00316	*petN-psbM*	LSC	0.00609
ndhC	LSC	0.00278	*psbM-trnD-GUC*	LSC	0.00722
atpE	LSC	0.00097	*trnE-UUC-trnT-GGU*	LSC	0.00692
atpB	LSC	0.00238	*trnT-GGU-psbD*	LSC	0.00830
rbcL	LSC	0.00352	*psbC-trnS-UGA*	LSC	0.00647
accD	LSC	0.00379	*trnS-UGA-psbZ*	LSC	0.00389
ycf4	LSC	0.00535	*psbZ-trnG-GCC*	LSC	0.00605
cemA	LSC	0.00265	*psaA-ycf3*	LSC	0.00487
petA	LSC	0.00188	*ycf3 intron*	LSC	0.00450
psbE	LSC	0.00067	*ycf3-trnS-GGA*	LSC	0.00837
rpl33	LSC	0.00513	*trnS-GGA-rps4*	LSC	0.00850
rps18	LSC	0.00310	*rps4-trnT-UGU*	LSC	0.01217
rpl20	LSC	0.00304	*trnT-UGU-trnL-UAA*	LSC	0.00695
rps12	LSC	0.00127	*trnL-UAA intron*	LSC	0.00532
clpP	LSC	0.00080	*trnL-UAA-trnF-GAA*	LSC	0.00636
psbB	LSC	0.00156	*trnF-GAA-ndhJ*	LSC	0.00927
psbH	LSC	0.00477	*ndhC-trnV-UAC*	LSC	0.00909
petB	LSC	0.00176	*trnV-UAC intron*	LSC	0.00229
petD	LSC	0.00272	*trnM-CAU-atpE*	LSC	0.00729
rpoA	LSC	0.00425	*atpB-rbcL*	LSC	0.00255
rps11	LSC	0.00040	*rbcL-accD*	LSC	0.00323
infA	LSC	0.00276	*accD-psaI*	LSC	0.00816
rps8	LSC	0.00339	*psaI-ycf4*	LSC	0.00754
rpl14	LSC	0.00128	*ycf4-cemA*	LSC	0.00421
rpl16	LSC	0.00322	*cemA-petA*	LSC	0.00342
rps3	LSC	0.00343	*petA-psbJ*	LSC	0.01642
rpl22	LSC	0.00573	*psbE-petL*	LSC	0.00577

rps19	LSC	0.00060	*trnP-UGG-psaJ*	LSC	0.00782
rpl2	LSC	0.00525	*psaJ-rpl33*	LSC	0.01086
rpl23	LSC	0.00310	*rps18-rpl20*	LSC	0.00500
ycf2	LSC	0.00747	*rpl20-rps12*	LSC	0.00329
ndhB	IR	0.00038	*rps12 intron*	LSC	0.00031
rps7	IR	0.00036	*rps12-ycf15*	LSC	0.00221
ndhF	SSC	0.00496	*clpP intron*	LSC	0.00670
ccsA	SSC	0.01460	*clpP-psbB*	LSC	0.00404
ndhD	SSC	0.00366	*psbB-psbT*	LSC	0.00412
psaC	SSC	0.00249	*petB intron*	LSC	0.00709
ndhE	SSC	0.00750	*petD intron*	LSC	0.00407
ndhG	SSC	0.00330	*rps8-rpl14*	LSC	0.01254
ndhI	SSC	0.00541	*rpl16 intron*	LSC	0.00663
ndhA	SSC	0.00292	*rpl2 intron*	LSC	0.00533
ndhH	SSC	0.00380	*ycf2-trnL-CAA*	IR	0.00189
rps15	SSC	0.00421	*trnL-CAA-ndhB*	IR	0.00087
ycf1	SSC	0.00718	*ndhB intron*	IR	0.00084
			ndhB-rps7	IR	0.00429
			ycf15-trnV-GAC	IR	0.00329
			trnV-GAC-rrn16	IR	0.00000
			rrn16-trnI-GAU	IR	0.00000
			trnI-GAU intron	IR	0.00070
			trnA-UGC intron	IR	0.00041
			rrn4.5-rrn5	IR	0.00064
			rrn5-trnR-ACG	IR	0.00000
			trnR-ACG-trnN-GUU	IR	0.00029
			trnN-GUU-ycf1	IR	0.00315
			ndhF-rpl32	SSC	0.01354
			rpl32-trnL-UAG	SSC	0.01317
			ccsA-ndhD	SSC	0.02613
			psaC-ndhE	SSC	0.00560
			ndhE-ndhG	SSC	0.00423
			ndhG-ndhI	SSC	0.00660
			ndhA intron	SSC	0.00493
			rps15-ycf1	SSC	0.00518

Table S9 Codon usage and relative synonymous codon usage (RSCU) values of protein-coding genes of the thirteen plastomes. These data were visualized in Figure S3.

		L. sibirica		*L. schrenkiana*		*L. seseloides*		*L. buchtormensis*		*L. incana*		*L. jinanensis*		*L. lanzhouensis*	
Amino Acid	Codon	Number	RSCU	Number	RSCU	Number	RSCU	Number	RSCU	Number	RSCU	Number	RSCU	Number	RSCU
Phe	UUU	809	1.35	809	1.35	810	1.35	810	1.36	810	1.35	790	1.35	804	1.35
	UUC	387	0.65	387	0.65	387	0.65	385	0.64	388	0.65	378	0.65	386	0.65
Leu	UUA	746	2.01	746	2.01	749	2.01	750	2.01	751	2.02	731	2.01	743	2
	UUG	439	1.18	441	1.19	440	1.18	442	1.18	439	1.18	428	1.17	443	1.19
	CUU	470	1.27	470	1.26	471	1.26	471	1.26	467	1.25	461	1.26	471	1.27
	CUC	144	0.39	144	0.39	146	0.39	144	0.39	145	0.39	144	0.4	144	0.39
	CUA	296	0.8	297	0.8	301	0.81	301	0.81	300	0.81	289	0.79	296	0.8
	CUG	134	0.36	134	0.36	134	0.36	134	0.36	134	0.36	134	0.37	131	0.35
Ile	AUU	868	1.44	868	1.44	870	1.45	869	1.44	864	1.44	854	1.45	866	1.45
	AUC	339	0.56	340	0.57	337	0.56	339	0.56	340	0.57	334	0.57	335	0.56
	AUA	598	0.99	597	0.99	599	1	600	1	596	0.99	583	0.99	594	0.99
Met	AUG	486	1	485	1	485	1	487	1	485	1	481	1	484	1
Val	GUU	443	1.51	443	1.51	442	1.51	443	1.51	441	1.51	434	1.5	440	1.5
	GUC	139	0.47	139	0.47	138	0.47	139	0.47	138	0.47	134	0.46	132	0.45
	GUA	424	1.44	425	1.45	426	1.45	425	1.45	426	1.46	421	1.45	429	1.47
	GUG	169	0.58	168	0.57	168	0.57	169	0.57	166	0.57	170	0.59	170	0.58
Ser	UCU	449	1.72	449	1.72	450	1.73	449	1.72	451	1.73	441	1.72	449	1.73
	UCC	245	0.94	245	0.94	246	0.95	247	0.95	244	0.94	245	0.96	242	0.93
	UCA	294	1.13	294	1.13	292	1.12	292	1.12	291	1.12	281	1.1	287	1.11
	UCG	169	0.65	167	0.64	167	0.64	167	0.64	170	0.65	164	0.64	165	0.64
Pro	CCU	348	1.58	348	1.57	351	1.58	349	1.57	345	1.57	345	1.58	351	1.59
	CCC	166	0.75	166	0.75	165	0.74	165	0.74	164	0.75	165	0.75	164	0.74
	CCA	229	1.04	228	1.03	233	1.05	233	1.05	231	1.05	227	1.04	228	1.03
	CCG	140	0.63	143	0.65	139	0.63	141	0.64	137	0.62	139	0.63	141	0.64
Thr	ACU	450	1.63	450	1.63	451	1.63	451	1.64	448	1.63	439	1.62	446	1.62
	ACC	205	0.74	205	0.74	206	0.75	203	0.74	204	0.74	205	0.75	207	0.75
	ACA	327	1.19	327	1.19	326	1.18	325	1.18	322	1.17	322	1.18	324	1.18
	ACG	121	0.44	121	0.44	122	0.44	122	0.44	123	0.45	121	0.45	122	0.44
Ala	GCU	528	1.8	529	1.8	526	1.79	527	1.79	528	1.81	522	1.79	529	1.8

	GCC	185	0.63	186	0.63	187	0.64	187	0.64	185	0.63	186	0.64	188	0.64
	GCA	322	1.1	323	1.1	325	1.11	326	1.11	321	1.1	319	1.1	322	1.1
	GCG	136	0.46	136	0.46	136	0.46	137	0.47	136	0.46	138	0.47	137	0.47
Tyr	UAU	641	1.59	640	1.59	644	1.59	641	1.59	644	1.59	634	1.59	645	1.6
	UAC	166	0.41	166	0.41	164	0.41	164	0.41	167	0.41	162	0.41	162	0.4
TER	UAA	29	1.64	29	1.64	29	1.64	30	1.7	29	1.64	29	1.64	30	1.7
	UAG	13	0.74	13	0.74	13	0.74	12	0.68	13	0.74	13	0.74	12	0.68
His	CAU	389	1.51	390	1.51	389	1.5	389	1.5	389	1.51	388	1.52	388	1.5
	CAC	125	0.49	127	0.49	128	0.5	129	0.5	126	0.49	124	0.48	129	0.5
Gln	CAA	579	1.51	579	1.51	573	1.51	574	1.51	571	1.51	567	1.51	572	1.51
	CAG	187	0.49	187	0.49	187	0.49	187	0.49	185	0.49	186	0.49	187	0.49
Asn	AAU	753	1.51	753	1.51	754	1.52	758	1.52	757	1.52	747	1.52	754	1.52
	AAC	243	0.49	242	0.49	237	0.48	240	0.48	240	0.48	237	0.48	241	0.48
Lys	AAA	837	1.51	837	1.51	841	1.51	839	1.51	842	1.52	842	1.52	849	1.52
	AAG	273	0.49	273	0.49	272	0.49	269	0.49	269	0.48	265	0.48	268	0.48
Asp	GAU	682	1.59	684	1.59	686	1.6	688	1.59	681	1.59	681	1.6	691	1.59
	GAC	175	0.41	175	0.41	173	0.4	176	0.41	174	0.41	170	0.4	176	0.41
Glu	GAA	824	1.51	824	1.51	822	1.51	821	1.5	823	1.51	821	1.51	825	1.51
	GAG	267	0.49	267	0.49	267	0.49	272	0.5	269	0.49	267	0.49	266	0.49
Cys	UGU	165	1.52	165	1.52	164	1.52	165	1.53	162	1.52	162	1.53	166	1.52
	UGC	52	0.48	52	0.48	52	0.48	51	0.47	51	0.48	50	0.47	52	0.48
TER	UGA	11	0.62	11	0.62	11	0.62	11	0.62	11	0.62	11	0.62	11	0.62
Trp	UGG	381	1	381	1	379	1	379	1	380	1	375	1	375	1
Arg	CGU	277	1.34	277	1.33	278	1.34	277	1.34	278	1.34	280	1.35	281	1.35
	CGC	86	0.42	86	0.41	83	0.4	84	0.41	84	0.4	79	0.38	81	0.39
	CGA	287	1.39	288	1.39	287	1.38	288	1.39	291	1.4	292	1.41	291	1.4
	CGG	95	0.46	95	0.46	95	0.46	95	0.46	97	0.47	95	0.46	95	0.46
Ser	AGU	323	1.24	324	1.24	319	1.23	321	1.23	324	1.24	321	1.25	325	1.25
	AGC	86	0.33	86	0.33	86	0.33	86	0.33	85	0.33	85	0.33	86	0.33
Arg	AGA	375	1.81	375	1.81	377	1.82	375	1.81	371	1.79	370	1.79	376	1.81
	AGG	123	0.59	124	0.6	126	0.61	124	0.6	124	0.6	124	0.6	122	0.59
Gly	GGU	498	1.34	497	1.33	491	1.32	496	1.33	496	1.33	490	1.33	496	1.33
	GGC	170	0.46	171	0.46	173	0.46	170	0.46	170	0.46	167	0.45	171	0.46

	GGA	565	1.51	565	1.51	565	1.52	566	1.52	562	1.51	556	1.51	563	1.51
	GGG	259	0.69	259	0.69	260	0.7	262	0.7	259	0.7	258	0.7	260	0.7
Total		21141		21152		21150		21168		21114		20873		21116	

cont'd

		L. spodotrichoma		*L. acaulis*		*L. grubovii*		*L. iliensis*		*L. condensata*		*L. depressa*	
Amino Acid	Codon	Number	RSCU	Number	RSCU	Number	RSCU	Number	RSCU	Number	RSCU	Number	RSCU
Phe	UUU	812	1.35	813	1.36	807	1.35	809	1.35	814	1.36	803	1.35
	UUC	387	0.65	386	0.64	390	0.65	389	0.65	385	0.64	383	0.65
Leu	UUA	751	2.02	752	2.02	749	2.01	753	2.01	748	2	745	2
	UUG	436	1.17	436	1.17	443	1.19	444	1.19	444	1.19	439	1.18
	CUU	473	1.27	473	1.27	468	1.25	472	1.26	475	1.27	471	1.26
	CUC	145	0.39	146	0.39	145	0.39	145	0.39	143	0.38	146	0.39
	CUA	297	0.8	298	0.8	302	0.81	304	0.81	301	0.8	295	0.79
	CUG	133	0.36	134	0.36	131	0.35	130	0.35	135	0.36	139	0.37
Ile	AUU	867	1.44	866	1.44	867	1.44	871	1.44	876	1.44	868	1.44
	AUC	337	0.56	339	0.56	345	0.57	346	0.57	342	0.56	341	0.57
	AUA	601	1	601	1	594	0.99	594	0.98	604	0.99	600	1
Met	AUG	485	1	485	1	485	1	487	1	487	1	477	1
Val	GUU	445	1.51	448	1.52	445	1.51	445	1.51	445	1.51	445	1.51
	GUC	135	0.46	135	0.46	138	0.47	138	0.47	138	0.47	135	0.46
	GUA	428	1.45	426	1.44	425	1.44	427	1.45	423	1.44	424	1.44
	GUG	172	0.58	173	0.59	171	0.58	172	0.58	170	0.58	172	0.59
Ser	UCU	450	1.73	454	1.74	448	1.72	450	1.72	452	1.73	440	1.7
	UCC	246	0.95	238	0.91	248	0.95	246	0.94	240	0.92	251	0.97
	UCA	289	1.11	295	1.13	293	1.12	293	1.12	297	1.13	289	1.12
	UCG	168	0.65	164	0.63	169	0.65	170	0.65	167	0.64	169	0.65
Pro	CCU	350	1.58	349	1.57	349	1.57	350	1.58	349	1.57	355	1.6
	CCC	164	0.74	166	0.75	165	0.74	164	0.74	166	0.75	161	0.73
	CCA	235	1.06	237	1.07	235	1.06	235	1.06	235	1.06	235	1.06
	CCG	137	0.62	137	0.62	139	0.63	139	0.63	139	0.63	136	0.61
Thr	ACU	453	1.64	449	1.63	450	1.63	450	1.63	449	1.63	453	1.64

	ACC	205	0.74	206	0.75	208	0.75	207	0.75	207	0.75	198	0.72
	ACA	326	1.18	324	1.18	324	1.17	325	1.18	326	1.18	329	1.19
	ACG	123	0.44	122	0.44	122	0.44	121	0.44	121	0.44	123	0.45
Ala	GCU	525	1.8	526	1.79	527	1.79	528	1.8	529	1.8	525	1.8
	GCC	188	0.64	188	0.64	190	0.65	190	0.65	188	0.64	184	0.63
	GCA	321	1.1	321	1.09	324	1.1	324	1.1	322	1.1	322	1.1
	GCG	135	0.46	138	0.47	134	0.46	133	0.45	137	0.47	138	0.47
Tyr	UAU	651	1.6	648	1.59	645	1.6	646	1.6	650	1.6	656	1.61
	UAC	163	0.4	168	0.41	160	0.4	160	0.4	163	0.4	161	0.39
TER	UAA	30	1.7	29	1.64	29	1.64	28	1.58	29	1.64	31	1.75
	UAG	12	0.68	13	0.74	12	0.68	13	0.74	11	0.62	12	0.68
His	CAU	391	1.51	392	1.52	392	1.52	392	1.52	389	1.52	388	1.51
	CAC	128	0.49	124	0.48	124	0.48	125	0.48	124	0.48	125	0.49
Gln	CAA	571	1.51	571	1.5	577	1.51	577	1.51	575	1.51	572	1.51
	CAG	186	0.49	188	0.5	188	0.49	187	0.49	187	0.49	185	0.49
Asn	AAU	761	1.52	762	1.53	761	1.52	763	1.52	753	1.51	754	1.52
	AAC	243	0.48	237	0.47	239	0.48	240	0.48	242	0.49	237	0.48
Lys	AAA	851	1.52	857	1.53	847	1.51	845	1.51	852	1.52	841	1.52
	AAG	271	0.48	263	0.47	272	0.49	271	0.49	271	0.48	263	0.48
Asp	GAU	685	1.6	681	1.6	687	1.61	686	1.6	684	1.59	677	1.59
	GAC	172	0.4	170	0.4	169	0.39	171	0.4	174	0.41	173	0.41
Glu	GAA	829	1.51	823	1.5	818	1.5	818	1.5	824	1.51	829	1.51
	GAG	268	0.49	271	0.5	273	0.5	274	0.5	270	0.49	270	0.49
Cys	UGU	166	1.52	167	1.53	165	1.52	164	1.51	168	1.53	164	1.55
	UGC	52	0.48	52	0.47	52	0.48	53	0.49	51	0.47	47	0.45
TER	UGA	11	0.62	11	0.62	12	0.68	12	0.68	13	0.74	10	0.57
Trp	UGG	380	1	379	1	378	1	378	1	382	1	379	1
Arg	CGU	281	1.35	277	1.34	280	1.35	281	1.35	280	1.34	279	1.35
	CGC	81	0.39	82	0.4	80	0.38	80	0.38	82	0.39	84	0.41
	CGA	292	1.4	282	1.37	290	1.39	292	1.4	293	1.4	287	1.39
	CGG	97	0.47	98	0.48	97	0.47	96	0.46	93	0.45	94	0.45
Ser	AGU	321	1.23	324	1.24	321	1.23	322	1.23	327	1.25	316	1.22
	AGC	87	0.33	87	0.33	86	0.33	86	0.33	88	0.34	89	0.34

Arg	AGA	376	1.8	377	1.83	377	1.81	378	1.81	380	1.82	370	1.79
	AGG	123	0.59	121	0.59	125	0.6	123	0.59	125	0.6	127	0.61
Gly	GGU	497	1.33	498	1.34	497	1.33	497	1.33	498	1.34	498	1.34
	GGC	173	0.46	168	0.45	171	0.46	172	0.46	172	0.46	169	0.46
	GGA	564	1.51	564	1.51	566	1.52	565	1.51	562	1.51	559	1.51
	GGG	259	0.69	262	0.7	257	0.69	258	0.69	259	0.69	258	0.7
Total		21191		21171		21177		21204		21225		21095	

Table S10 The morphological comparision of different *Libanotis* in this study. Data based on FOC (2005), JSTOR, CVH and sampled specimens.

sections			Sect. *Libanotis*			Sect. *Pseudolibanotis*	
taxa	***L. sibirica***	***L. schrenkiana***	***L. seseloides***	***L. incana***	***L. condensata***	***L. depressa***	***L. acaulis***
Stem (development)	Well developed	Well developed	Well developed	Well developed	Well developed	Not developed	Not developed
Stem (height in cm)	30–100(–120)	40–110(–130)	30-130	(25-)30-60(-90)	20-90	2-5	4-8
ultimate segments	ovate or rhombic	linear-lanceolate	linear-lanceolate	linear	linear-lanceolate	linear-lanceolate	linear-elliptic
leaf blade	green, abaxially sometimes gray-green	green, abaxially sometimes gray-green	green, abaxially sometimes gray-green	green, gray-white pubescent on both surfaces	green, abaxially sometimes gray-green	green	green
rays	(20–)35–50	15-25(-40)	8-20	(15-)20-35	15-25	6-10	7-10
bracts	absent or few, linear, very small	absent or 3-9, subulate to linear	absent, occasionally 1-5, subulate or linear	absent or few, linear	6-10, linear	1-2,, usually obscured by leaf rosette	1-2
bracteoles	12–15, linear	10-12, linear	8-14, linear	12-15, ovate lanceolate	several, linear, exceeding flowers, villous	7-11, acicular, very unequal	10-20, lanceolate, unequal
calyx teeth	triangular-lanceolate	triangular-lanceolate	triangular or lanceolate	lanceolate	subulate	lanceolate, very conspicuous	triangular-lanceolate
petals (abaxially pubescence)	glabrous	glabrous	puberulent	puberulent	glabrous or sparsely pubescent	glabrous	glabrous
fruits (shape)	ovoid-ellipsoid	ellipsoid	oblong-ovoid	ovate or ellipsoid	ellipsoid	oblong or suborbicular	oblong
mericarps (pubescence)	glabrous or slightly puberulent	glabrous or slightly puberulent	glabrous or slightly puberulent	densely pubescent	densely villous	densely scaly-hispid	densely scaly-tomentose
mericarps (size, mm)	3–4 × 1.5–2	2-3.5 × 0.7-2	2.5-3.5 × ca. 1.5	3.2-4.7×2-3.5	3-4×2-3	2-2.5 × 1.3-1.5	2-2.5 × 1.5-1.8

ribs	subequal, shortly keeled	subequal, shortly keeled	unequal, lateral ribs slightly broader than the dorsal	unequal, slightly flattened dorsally	unequal, dorsal ribs keeled, acute, marginal ribs winged	subequal, filiform, prominent	unequal, dorsal ribs keeled, acute, marginal ribs shortly winged
vittae	1 in each furrow, 2 on commissure	2 in each furrow, 2 on commissure	3-4 in each furrow, 6 on commissure	1 in each furrow, 2 on commissure	2-4 in each furrow, 4 on commissure	1 in each furrow, 2 on commissure	2-3 in each furrow, 4-6 on commissure

cont'd

sections	Sect. *Eriotis*					
taxa	***L. spodotrichoma***	***L. iliensis***	***L. lanzhouensis***	***L. jinanensis***	***L. grubovii***	***L. buchtormensis***
Stem (development)	Well developed	Well developed	Well developed	Well developed	Well developed	Well developed
Stem (height in cm)	(25-)40-80	100-200	30-90	25-50	(20-)30-50(-80)	20-80
ultimate segments	obovate-cuneate or ovate	linear	gray-green, linear or rhombic	obovate-cuneate	oblong to linear-lanceolate	oblong-ovate or lanceolate
leaf blade	blue-green or gray-green	green	blue-green	gray-green	blue-green	green
rays	5-12	10-15(-20)	2-4	4-9	6-10(-14)	30-50
bracts	absent	5-10, white, ovate-lanceolate	absent or occasionally 1 (like uppermost leaf)	absent, occasionally 1-2	8-10, linear-lanceolate	absent, occasionally few, linear, small
bracteoles	7-10, lanceolate-linear	5-10, ovate-lanceolate	5-7, linear-lanceolate	10-12, narrowly triangular	8-10, similar to bracts	8-15(-20)
calyx teeth	narrowly triangular or lanceolate-subulate	short-triangular or subulate	subulate	triangular-lanceolate	lanceolate triangular	linear-subulate to lanceolate
petals (abaxially pubescence)	villous	pubescent	puberulous	densely puberulent	pubescent	sparsely pubescent

fruits (shape)	obovate-oblong	oblong or ellipsoid	ellipsoid	oblong-ovoid	ovoid or ellipsoid	ellipsoid
mericarps (pubescence)	grayish-villous	densely pubescent	densely villous	densely white pubescent	shortly hairy	densely tomentose or squamose-scaly
mericarps (size, mm)	3-4(-6) × 1-1.5	2.8-4 × 0.6-0.7	2.8-3.2 × 1.4-1.5	3-4 × 1.5-1.8	4.8-5.5 × 2.7-4	2-4.5 × 1.5-2.5
ribs	slightly prominent, filiform	filiform	equal, filiform, slightly prominent	equal, shortly keeled	unequal, dorsal ribs keeled, acute, marginal ribs shortly winged	equal, prominent and acute keeled
vittae	1 in each furrow, 2 on commissure	1 in each furrow, 2 on commissure	1(-2) in each furrow, 2 on commissure	1 in each furrow, 2 on commissure	(1-)2-3 in each furrow, (2-)4-6 on commissure	1 in each furrow, 2 on commissure
